# Multidrug resistance in bacteria causing community-acquired urinary tract infections among adult outpatients attending primary healthcare facilities in Mwanza, Tanzania

**DOI:** 10.1371/journal.pgph.0005638

**Published:** 2026-10-08

**Authors:** Eunice G. Emmanuel, Ashura Khamis, Elikana Michael, Dorina Muhizi, James Thomas, Bernard Okamo, Farida I. Mkassy, Eveline T. Konje, Vitus Silago, Martha F. Mushi, Stephen E. Mshana

**Affiliations:** 1 Weil Bugando School of Medicine, Catholic University of Health and Allied Sciences, Mwanza, Tanzania; 2 Pharmacy Department, Igoma Health Centre, Mwanza, Tanzania; 3 Department of Biochemistry and Molecular Biology Catholic University of Health and Allied Sciences, Mwanza, Tanzania; 4 Department of Epidemiology, Biostatistics and Behavioural science, School of Public Health, Catholic University of Health and Allied Sciences, Mwanza, Tanzania; 5 Department of Microbiology and immunology, Weil Bugando School of Medicine, Catholic University of Health and Allied Sciences, Mwanza, Tanzania; University of St Andrews, UNITED KINGDOM OF GREAT BRITAIN AND NORTHERN IRELAND

## Abstract

Urinary tract infections (UTIs) are a common indication for antibiotic use among adult outpatients in low- and middle-income countries, yet data on community-acquired UTIs (CA-UTIs) and antimicrobial resistance at primary healthcare facilities remain limited. A facility-based cross-sectional study was conducted from May to August 2024 at Igoma and Buzuruga Health Centres in Mwanza, Tanzania. Symptomatic adults without hospitalization within the preceding 30 days or urinary catheterization were enrolled. Clinical and demographic data were collected, and midstream urine specimens were cultured, followed by antimicrobial susceptibility testing using the disc diffusion method. Of 1,005 participants, the median age was 32 years [IQR: 23–49], 727 (72.3%) were female, and 64.3% reported to have been diagnosed with UTI within the previous six months. The median symptom duration before presentation was 8 days [IQR: 5–15]. Culture-confirmed UTI was detected in 221 participants (22.0%; 95% CI: 19.5–24.7), with *Escherichia coli* (32.9%) and *Staphylococcus aureus* (24.7%) being the most frequently isolated pathogens. Only 35.7% of culture-confirmed cases are presented with classic urinary symptoms of dysuria, frequency, or urgency. *E. coli* showed high resistance to ampicillin (93.4%), ciprofloxacin (62.3%), trimethoprim-sulfamethoxazole (60.7%), amoxicillin-clavulanic acid (57.4%), and tetracycline (55.7%); 82.8% were multidrug-resistant and 20.3% exhibited extended-spectrum beta-lactamase phenotypes. Among *S. aureus* isolates, 64.6% were cefoxitin-resistant, indicating methicillin resistance. CA-UTIs therefore constitutes a substantial burden among adult primary-care outpatients, accompanied by high antimicrobial resistance and frequent atypical clinical presentations. Improved access to UTI diagnostics, strengthened antimicrobial stewardship, and locally informed treatment guidelines are needed, particularly given the high resistance to commonly used empirical antibiotics.

## Background

Urinary tract infections (UTIs) are a prevalent health concern among patients visiting outpatient clinics in low- and middle-income countries (LMICs). In these regions, UTIs significantly contribute to morbidity, elevated treatment expenses, and mortality, especially among those seeking care at primary healthcare facilities [1,2]. Limited diagnostic resources coupled with insufficient microbiological evidence often compromise effective patient management. However, UTI remains one of the most common reasons for antibiotic prescription and the most frequent reason for hospital attendance, surpassed only by malaria [3,4]. In Tanzania, the prevalence of UTI is reported to be different among populations, ranging from 11-27.4% [5–9].

Enterobacterales such as *Escherichia coli* and *Klebsiella pneumoniae* are the leading causes of urinary tract infections (UTIs) in both community and healthcare settings [1,9]. Many of these Gram-negative isolates produce extended-spectrum beta-lactamases (ESBLs), a major mechanism of antimicrobial resistance [10,11]. Among Gram-positive bacteria, *Staphylococcus aureus* is commonly reported as a leading uropathogens [12]. In Tanzania, previous studies have shown high rates of multidrug resistance among UTI pathogens, ranging from 22.4%–60.3% in Gram-positive bacteria and 45.4%–52.2% in Gram-negative bacteria [4,9,13,14].

Several factors increase the risk of community-acquired urinary tract infections (CA-UTIs). Women, older adults, and pregnant women are particularly vulnerable due to anatomical, physiological, and hormonal factors [15]. Behavioural factors such as poor hygiene, frequent sexual activity, and the use of spermicides or diaphragms also increase susceptibility. Medical conditions including diabetes mellitus, urinary tract obstruction, kidney stones, congenital abnormalities, and enlarged prostate further predispose individuals to infection by promoting bacterial growth and impairing urine flow. In addition, antibiotic use and catheterization can disrupt normal flora and facilitate bacterial colonization [4]. Since antimicrobial resistance patterns vary across regions and over time, local surveillance is important for guiding appropriate empirical treatment.

Previous studies in Tanzania have mainly focused on tertiary hospitals or specific groups, such as pregnant women, children, and people living with HIV [4–7,9,13,14], leaving limited evidence on community-acquired UTIs among general adult outpatients attending primary healthcare facilities. In addition, Tanzania’s standard treatment guidelines recommend empirical treatment with ciprofloxacin, amoxicillin-clavulanic acid, nitrofurantoin and trimethoprim-sulfamethoxazole in lower health facility [16], which are commonly prescribed in primary healthcare settings and may also be accessible without prescription in some community settings. However, increasing resistance among community uropathogens may compromise the effectiveness of these agents, particularly at primary healthcare facilities where treatment is often initiated empirically because urine culture and antimicrobial susceptibility testing are not routinely available.Understanding the prevalence, causative organisms, and resistance patterns in this setting is essential for informing targeted interventions, updating treatment guidelines, and strengthening antimicrobial stewardship programs.

This study aimed to determine the prevalence of CA-UTIs, identify the causative bacterial pathogens, and characterize their antimicrobial susceptibility profiles among adult outpatients attending primary healthcare facilities in Mwanza, Tanzania. By focusing on the primary care setting where most patients first present, this research provides critical insights into the current state of AMR in community settings and informs public health strategies to combat the rising threat of multidrug-resistant infections.

## Methodology

### Study design and settings

This prospective cross-sectional study was conducted at two primary healthcare facilities, Igoma and Buzuruga Health Centres in Mwanza, Tanzania, from 15th May to 30th August 2024. The study was implemented using predefined objectives, standardized enrolment criteria, and systematic microbiological procedures. Buzuruga Health Centre serves approximately 2,266 patients per month, while Igoma Health Centre attends about 1,500 outpatients monthly.

### Sample size and study population

The study population included adult outpatients (≥18 years) presenting with symptoms suggestive of urinary tract infection (UTI). In accordance with established clinical guidelines [17] and the Tanzanian Standard Treatment Guidelines, participants were enrolled after clinical assessment by outpatient clinicians and were required to present with at least two suggestive symptoms, including dysuria, urinary frequency, urinary urgency, suprapubic pain, or lower abdominal pain.

The minimum sample size was obtained by the Kish-Leslie formula [18] using a 24.7% prevalence of CA-UTIs previously reported by Silago et *al*.[9]. To account for potential non-response and ensure adequate power for subgroup analyses, the sample was increased, resulting in a target enrollment of 1,005 participants. Pregnant women, patients with a history of admission within 30 days on the day of sample collection, and those with an indwelling urinary catheter at the time of study were excluded.

### Data and sample collections

Demographic and clinical data were collected using Epicollect5, a mobile data-gathering platform developed by the CGPS Team of Oxford BDI and publicly available at https://five.epicollect.net. Information on prior antibiotic use was collected for the 14 days preceding enrolment. This recall period was selected to improve the accuracy of self-reported exposure and to capture recent antibiotic use that could suppress bacterial growth and influence urine culture results. However, it may not capture earlier antibiotic exposure relevant to the selection of resistant organisms. About 3–5 ml of clean catch midstream urine was collected by the patients after receiving instructions from the research assistant, using a sterile, wide-mouthed, labelled container. The urine sample was transported to the microbiology research laboratory of CUHAS for processing within 2 hours of collection. For women, urine samples were tested for HCG to exclude pregnancy [19]. Urine was divided to two portions for microscopy/dipstick analysis and culture. All laboratory data were documented on the laboratory logbook.

### Urine culture

Urine samples were inoculated onto agar media (Blood Agar, MacConkey Agar and CLED) and incubated at 37^o^c for 18–24 hours. Growth estimate of ≥1000 colonies from 10µl inoculating loop for both men and women was considered as significant bacteriuria and thus UTI [20]. In-house-prepared conventional biochemical identification tests including: catalase, coagulase, oxidase, indole, citrate, urease, and sugar fermentation tests were used for the preliminary identification of bacteria isolates to their possible species levels as previously described [21]. Gram-positive and Gram-negative bacteria were identified using morphological and biochemical tests as previously described [22]. Unidentified isolates were further analysed using Matrix-Assisted Laser Desorption Ionization–Time of Flight (MALDI-TOF) mass spectrometry at the National Public Health Laboratory (NPHL) [23]. Antimicrobial susceptibility testing was performed as per CLSI guidelines [24].

Screening for extended-spectrum beta-lactamase (ESBL) production among Enterobacterales isolates was initially performed using ceftriaxone (30 µg) and ceftazidime (30 µg) discs during routine antimicrobial susceptibility testing. Isolates showing reduced susceptibility to either antibiotic were subjected to ESBL confirmation using the combination disc method according to CLSI guidelines [24].Confirmation of ESBL production was defined by an increase of ≥5 mm in the inhibition zone diameter for ceftazidime-clavulanic acid compared to ceftazidime alone.

For Gram-positive isolates, erythromycin and clindamycin discs were additionally used to screen for inducible macrolide-lincosamide-streptogramin B (iMLSB) resistance using the D-test according to CLSI guidelines [24]. Isolates showing resistance to erythromycin and susceptibility to clindamycin were subjected to the D-test by placing erythromycin and clindamycin discs 15–20 mm apart on Mueller-Hinton agar. Flattening of the clindamycin inhibition zone adjacent to the erythromycin disc (D-shaped zone) was interpreted as inducible clindamycin resistance. Erythromycin results were used only for D-test screening purposes and were not included in routine urinary antimicrobial susceptibility interpretation or multidrug resistance analysis.

### Quality control

Quality assurance measures encompassed monitoring reagent storage, equipment functionality, media sterility, and preparation of standardized bacterial suspensions. All stains and reagents were systematically labelled, dated, and stored under appropriate conditions. Refrigerator and incubator temperatures were recorded and tracked daily to ensure compliance with operational standards. Culture media were prepared in strict accordance with manufacturer instructions and evaluated for both performance and sterility. To maintain consistent inoculum density for susceptibility testing, a 0.5 McFarland standard was utilized; standard reference strains like *S. aureus* (ATCC 25923), *MRSA* (ATCC 29213), *E. coli* (ATCC 25922), ESBL-producing *Klebsiella pneumoniae* (ATCC 700603), and *Pseudomonas aeruginosa* (ATCC 27853) were employed as controls. Data cleaning and coding were conducted using STATA version 15, with records containing missing values excluded from analysis.

### Data analysis

Data were analysed using STATA software version 15 in line with the study’s objectives. Descriptive statistics were used to summarize data, Continuous variables were summarized as medians with interquartile ranges (IQR), while categorical variables were summarized as frequencies and proportions. Comparisons between categorical variables were performed using Chi-square test or Fisher’s exact test where appropriate. A p-value of <0.05 was considered statistically significant.

### Ethics statement

Ethical clearance was obtained from the Joint CUHAS/BMC Research Ethics and Review Committee (CREC) with certificate number CREC No 763/2024. All participants were requested to consent by signing written informed consent form. Privacy and confidentiality were maintained throughout the study. The urine culture results were timely shared to the manage clinicians to guide the management.

## Results

The study recruited a total of 1005 adult, patients with a clinical diagnosis of UTI. In general, majority of recruited patients were non-pregnant women 727(72.3%), about two third were married 610 (60.7%) and their median age was 32[23–49] years, as detailed in Table 1.

**Table 1:** Demographic and clinical characteristics of the studied population.

Variable		Buzuruga HC (566) n(%)	Igoma HC (439) n(%)	Total (1005)
Gender	Male	138(24.4)	140(31.9)	278(27.7)
	Female	428(75.6)	299(68.1)	727(72.3)
Age	Median [IQR]	32[23-50]	30[22-49]	32[23-49]
Education	No formal education	37(6.5)	65(14.8)	102(10.2)
	Primary	243(42.9)	216(49.2)	459(45.7)
	Secondary	220(38.9)	123(28.0)	343(34.1)
	University	66(11.7)	35(7.9)	101(10.1)
Marital status	Married	367(64.8)	243(55.4)	610(60.7)
	Single	199(35.2)	196(44.6)	395(39.3)
History of UTI	Yes	382(67.5)	264(60.1)	646(64.3)
	No	184(32.5)	175(39.9)	359(35.7)
Dysuria	Yes	137(24.2)	117(26.6)	254(25.3)
	No	429(75.8)	322(73.4)	751(74.7)
Urgency	Yes	54(9.5)	16(3.6)	70(6.9)
	No	512(90.5)	423(96.4)	935(93.0)
Frequency	Yes	66(11.7)	40(9.1)	106(10.6)
	No	500(88.3)	399(90.9)	899(89.4)
Nocturia	Yes	20(3.5)	22(5.0)	42(4.2)
	No	546(96.5)	417(94.9)	963(95.8)
Cloudy urine	Yes	58(10.3)	31(7.1)	89(8.9)
	No	508(89.7)	408(92.9)	916(91.1)
Smelling urine	Yes	40(7.1)	14(3.2)	54(5.4)
	No	526(92.9)	425(96.8)	951(94.6)
Lower abdominal pain	Yes	275(48.6)	237(53.9)	512(50.9)
	No	291(51.4)	202(46.0)	493(49.1)
Lower back pain	Yes	161(28.5)	212(48.3)	373(37.1)
	No	405(71.5)	227(51.7)	632(62.9)
Fever	Yes	377(66.6)	211(48.1)	588(58.5)
	No	189(33.4)	228(51.9)	417(41.5)
Duration of symptoms (days)		7 [3 –14]	14[7-28]	8[5 –15]
Presence of comorbidity	Yes	96(16.9)	26(5.9)	122(12.1)
	No	470(83.0)	413(94.1)	883(87.9)
Current antibiotic use	Yes	89(15.7)	40(9.1)	129(12.8)
	No	477(84.3)	399(90.9)	876(87.2)
Co-morbidity	High blood Pressure	26(27.1%)	12(46.2%)	38(31.1%)
	Diabetic Mellitus	19(19.8%)	6(23.1%)	25(20.5%)
	Chronic Back Pain	19(19.8%)	4(15.4%)	23(18.9%)
	Peptic Ulcer Diseases	23(23.9%)	0(0.0%)	23(18.9%)
	Others	9(9.4%)	4(15.4%)	13(10.7%)

Others included Benign prostatic hyperplasia 2; living with HIV 6; chronic leg pain 1; ovarian cyst 4

Regarding clinical history, 646 participants (64.3%) reported a previous episode of UTI within the past six months, and 129 (12.8%) had used antibiotics within the 14 days preceding enrollment. The median duration of symptoms before seeking care was 8 days (IQR: 5–15 days). Notably, only 359 participants (35.7%) presented with at least two of the classic urinary tract symptoms (dysuria, frequency, and urgency), while the majority presented with non-specific symptoms such as lower abdominal pain, fever, or lower back pain, highlighting the clinical diagnostic challenges in this setting. Patients recruited from Igoma health centre reported late to the health facility than those from Buzuruga health centre (14[7–28] vs. 7[3 –14] days), P = 0.001. as indicated in Table 1.

Among the recruited patients with other comorbidities 122, high blood pressure was the commonest, 38 (31.1%). A total of seven patients had more than one chronic condition (comorbidity) such as diabetic mellitus and hypertension (six patients), and one patient had both hypertension and peptic ulcer diseases making total count of 122.

### Prevalence of community-acquired UTI

Of the 1005 recruited patients with clinical presentation of urinary tract infections, 221(22%), 95% CI 19.4%-24.6% were microbiologically confirmed. Igoma health centre had 22.8%, (n = 100/439), 95% CI 18.9%-27% patients with microbiologically confirmed UTI while Buzuruga health centre had 21.4%, (n = 121/566), 95% CI 18%-25% patients.

### Proportions of bacterial species causing CA-UTIs

Of the 221 microbiologically confirmed cases, 194 were due to bacterial isolates, while 27 showed significant growth of Candida spp. which were not included in the bacterial antimicrobial resistance analysis. The majority of bacteria isolated were Gram-negative (59.8%; n = 116). Overall, the most frequently detected bacterial species were E. coli (32.9%; n = 64) and S. aureus (24.7%; n = 48). A similar pattern of predominant bacteria pathogens was observed at both Buzuruga Health Centre and Igoma Health Centre, however, the third and fourth most common isolates differed slightly between the two sites. At Buzuruga HC, Enterococcus spp. (10.5%; n = 11) and Acinetobacter spp. (9.5%; n = 10) were the commonest third and fourth pathogen, respectively while at Igoma Acinetobacter spp. (10.1%; n = 9) and K. pneumoniae complex (8.9%; n = 8) were common. Notably, all C. freundii strains were exclusively isolated at Buzuruga HC (Table 2).

**Table 2:** The proportions of bacterial species causing community-acquired urinary tract infections.

Isolate	All (n = 194)	Buzuruga HC (N = 105)	Igoma HC (N = 89)
	n(%)	n(%)	n(%)
*E. coli*	64 (32.9)	33 (31.4)	31 (34.8)
*S. aureus*	48 (24.7)	25 (23.8)	23 (25.8)
*Acinetobacter* spp.	19 (9.8)	10 (9.5)	9 (10.1)
*Enterococcus* spp.	16 (8.2)	11 (10.5)	5 (5.6)
*K. pneumoniae* complex	12 (6.2)	4 (3.8)	8 (8.9)
*S. pyogenes*	10 (5.2)	5 (4.8)	5(5.6)
*P. aeruginosa*	7 (3.6)	2 (1.9)	5 (5.6)
*C. freundii*	5 (2.6)	5 (4.8)	0 (0.0)
Others*	13 (6.7)	10 (9.5)	3 (3.4)

*Others: n = 13: *Morganella morganii* (n = 3), *Enterobacter cloacae* complex (n = 2), *Citrobacter koseri* (n = 1), *Klebsiella oxytoca* (n = 1), *Pseudomonas fluorescens* (n = 1), *Proteus mirabilis* (n = 1), *Pseudoglutamicibacter cumminsii* (n = 1), *Streptococcus agalactiae* (n = 1), *Staphylococcus epidermidis* (n = 1), and *Staphylococcus haemolyticus* (n = 1).

**Buzuruga HC (n = 10):** *Morganella morganii* (n = 3), *Enterobacter cloacae* complex (n = 1), *Citrobacter koseri* (n = 1), *Klebsiella oxytoca* (n = 1), *Pseudomonas fluorescens* (n = 1), *Pseudoglutamicibacter cumminsii* (n = 1), *Staphylococcus epidermidis* (n = 1), and *Staphylococcus haemolyticus* (n = 1).

**Igoma HC (n = 3):** *Enterobacter cloacae* complex (n = 1), *Proteus mirabilis* (n = 1), and *Streptococcus agalactiae* (n = 1).

### Percentage resistance of bacterial pathogens causing CA-UTIs

*Escherichia coli* demonstrated the highest levels of antimicrobial resistance to multiple antibiotics, including ciprofloxacin, amoxicillin-clavulanic acid, trimethoprim-sulfamethoxazole, tetracycline, and ampicillin. Among *E. coli* isolates, 45.3% were resistant to ceftriaxone, while 20.3% demonstrated positive phenotypes of extended-spectrum beta-lactamase (ESBL) production. Other members of the Enterobacterales group showed similarly high resistance rates to amoxicillin-clavulanic acid and ampicillin, with 12.0% detected with ESBL phenotypes, (Table 3).

**Table 3:** Percentage resistance of bacteria causing community-acquired urinary tract infection.

	Gram negative bacteria					
Antibiotic agents tested	*E. coli* (N = 64)		Other Enterobacterales (N = 25)		Non-fermentative Gram-negative bacteria (N = 27)	
	% Resistance	% Sensitive	% Resistance	% Sensitive	% Resistance	% Sensitive
AMP	58 (90.6)	6 (9.4)	25 (100)	0 (0.0)	NA	NA
TET	47 (73.4)	17 (26.6)	10 (40.0)	15 (60.0)	11 (47.8)	12 (52.2)
SXT	46 (73.0)	17 (27.0)	10 (40.0)	15 (60.0)	NA	
CIP	34 (53.1)	30 (46.9)	8 (32.0)	17 (68.0)	9 (33.3)	18 (66.7)
NIT	26 (40.6)	38 (59.4)	14 (56.0)	11 (44.0)	NA	NA
AMC	41 (64.1)	23 (35.9)	13 (52.0)	12 (48.0)	NA	
CRO	29 (45.3)	35 (54.7)	11 (44.0)	14 (56.00)	NA	
GEN	29 (45.3)	35 (54.7)	6 (25.0)	18 (75.0)	4 (15.4)	22 (86.4)
MEM	5 (7.9)	58 (92.1)	4 (16.0)	21 (84.0)	0 (0.0)	26 (100)
ESBL strains	13 (20.3)		3 (12.0)		NA	
***Antibiotic agents tested***	***Gram positive bacteria***					
	***S. aureus* (N = 48)**		**Other Gram-positive bacteria (N = 29)**			
	**% Resistance**	**% Sensitive**	**% Resistance**		**% Sensitive**	
ER	NA		28 (96.6)		1 (3.4)	
CIP	24 (50.0)	24 (50.0)	25 (86.2)		4 (13.8)	
SXT	31 (64.6)	17 (35.4)	24 (82.8)		5 (17.2)	
TET	28 (58.4)	20 (41.6)	22 (75.9)		7 (24.1)	
PEN	38 (79.2)	10 (20.8)	15 (51.7)		14 (48.3)	
CD	29 (31.3)	33 (68.7)	14 (48.3)		15 (51.7)	
GEN	11 (22.9)	37 (77.1)	11 (37.9)		18 (62.1)	
NIT	12 (25.0)	36 (75.0)	11 (37.9)		18 (62.1)	
FOX	31 (64.6)	17 (35.4)	NT		NT	

Key: AMP = ampicillin, TET = tetracycline, SXT = trimethoprim-sulfamethoxazole, CIP = ciprofloxacin, NIT = nitrofurantoin, AMC = amoxicillin-clavulanic acid, CRO = ceftriaxone, GEN = gentamicin, NA = not applicable, and MEM = meropenem; ER = erythromycin; CIP = ciprofloxacin; SXT = trimethoprim-sulfamethoxazole; TET = tetracycline; PEN = penicillin; CD = clindamycin; GEN = gentamicin; NIT-nitrofurantoin; and FOX = cefoxitin.

On the other hand, *S. aureus* demonstrated markedly high resistance rates (≥ 50%) to ciprofloxacin, tetracycline, trimethoprim-sulfamethoxazole, ampicillin, and erythromycin. Moreover, 64.6% of *S. aureus* strains were resistant to cefoxitin, hence methicillin-resistant *S.* *aureus* (MRSA). Similarly, other Gram-negative bacteria exhibited peak resistance (≥ 50%) to ampicillin, tetracycline, trimethoprim-sulfamethoxazole, ciprofloxacin, and erythromycin (Table 3).

### Proportions of multidrug resistance and resistance patterns of Gram-negative bacteria causing CA-UTIs

Overall, most Gram-negative bacteria (73.3%; 85/116) exhibited resistance to at least three classes of antimicrobials, with the number of classes ranging from three to six. By healthcare facility, the difference in proportions of MDR in Gram-negative bacteria isolated from patients attending at Buzuruga HC (70.5%; 43/61) and Igoma HC (76.4%; 42/55) was not statistically significant (p = 0.475). *E. coli* showed the highest proportion of multidrug resistance (MDR), with 82.8% (53/64) of isolates classified as MDR, including 15.1% (n = 8) that were resistant to six antimicrobial classes. The common resistance patterns in *E. coli* were AMP-TET-SXT-CIP-NIT-AMC-CRO-CAZ-FEP-GEN-AK-TZP (n = 3), AMP-TET-SXT-CIP-NIT-AMC-CRO-CAZ-FEP-GEN-TZP (n = 2), and AMP-TET-SXT-CIP-AMC-CRO-CAZ-FEP-AK-TZP (n = 2). Among other Enterobacterales, 44.0% (11/25) were MDR, with 27.3% (n = 3) also resistant to six classes. Similarly, 77.8% (22/27) of non-fermentative Gram-negative bacteria displayed MDR, with 9.1% (n = 2) resistant to six antimicrobial classes. The common resistance patterns in non-fermentative Gram-negative bacteria were SXT-NIT-CRO-CAZ-FEP (n = 2) and TET-SXT-NIT-CRO-FEP-TZP (n = 2) (Table 3). Detailed antimicrobial resistance patterns are provided in S1 File.

Among GPB, a substantial proportion of *S. aureus* isolates (83.3%; 40/48) were classified as MDR. Notably, 15.0% (6/40) of these MDR *S. aureus* strains exhibited resistance to as many as seven antibiotics. MDR phenotypes were significantly more prevalent among *S. aureus* isolates from patients at Igoma Health Centre compared to those from Buzuruga HC (95.6% vs. 72.0%; p = 0.019). The most frequently observed resistance patterns in *S. aureus* included CD-ER-AMP-SXT-TET-CIP-FOX (n = 3), ER-AMP-SXT-TET-CIP-FOX (n = 3), and ER-AMP-SXT-TET-NIT-CIP-FOX-GEN (n = 3). For other GPB, all isolates (100%; 29/29) were MDR. The most common resistance profiles among these included CD-ER-AMP-SXT-TET-CIP (n = 2), CD-ER-NIT-CIP (n = 2), and ER-AMP-SXT-TET-NIT-CIP (n = 2). Moreover, 24.1% (7/29) of these MDR GPB strains demonstrated resistance to six antimicrobial agents (Table 4). Detailed antimicrobial resistance patterns are shown on S2 File.

**Table 4:** Distribution of multidrug resistance and number of resistant antibiotic classes among bacterial isolates causing urinary tract infections in primary healthcare facilities.

Variable	Category	*E. coli* (N=64) n(%)	Other Enterobacterales (N=25) n(%)	Non-fermentative GNB (N=27) n(%)
Multidrug-resistant (MDR) strain	Yes	53 (82.8)	11 (44.0)	21 (77.8)
	No	11 (17.2)	14 (56.0)	6 (22.2)
The number of antibiotic classes to which the bacteria resist	3	10 (18.9)	2 (18.2)	10 (45.4)
	4	13 (24.5)	4 (36.4)	6 (27.3)
	5	22 (41.5)	2 (18.2)	3 (13.6)
	6	8 (15.1)	3 (27.3)	2 (9.1)
Health Centre for isolation of MDR strains	Buzuruga HC (N=43)	28 (65.1)	9 (20.9)	6 (13.9)
	Igoma HC (N=42)	25 (59.5)	12 (28.6)	5 (11.9)
**Variable**	**Category**	***S. aureus* (N=48)** **n(%)**		**Other Gram-positive bacteria (N=29)** **n(%)**
Multidrug-resistant (MDR) strain	Yes	40 (83.3)		29 (100)
	No	8 (16.7)		0 (0.0)
The number of antibiotic classes to which the bacteria resist	3	9 (22.5)		6 (20.7)
	4	11 (27.5)		8 (27.6)
	5	12 (30.0)		8 (27.6)
	6	2 (5.0)		7 (24.1)
	7	6 (15.0)		0 (0.0)
Health centre for isolation of MDR strains	Buzuruga HC	18/25 (72.0)		18/18 (100)
	Igoma HC	22/23 (95.6)		11/11 (100)

## Discussion

This study provides important insights into the bacterial causes and antimicrobial resistance patterns of community-acquired urinary tract infections among patients attending primary healthcare facilities in Mwanza, Tanzania. The findings demonstrate that *Escherichia coli* remains the predominant uropathogens, with a substantial proportion of isolates exhibiting multidrug resistance. Notably, high resistance was observed to several commonly used antibiotics, raising concerns about the effectiveness of current empirical treatment practices in primary healthcare settings. Although only a small proportion of participants reported prior antibiotic use, the observed resistance patterns suggest ongoing antimicrobial pressure within the community. Together, these findings highlight the growing public health challenge of antimicrobial resistance in community-acquired infections and underscore the need for strengthened surveillance, improved diagnostic capacity, and evidence-based antimicrobial stewardship to guide appropriate treatment.

The high prevalence of multidrug-resistant uropathogens observed in this study likely reflects multiple drivers operating at the community and healthcare levels. In many low- and middle-income countries, including Tanzania, antibiotics are frequently used empirically for urinary tract infections due to limited access to microbiological diagnostic services at primary healthcare facilities [25,26]. In addition, inappropriate antibiotic use, including self-medication, incomplete treatment courses, and over-the-counter access to antimicrobials, has been reported as a major contributor to the emergence and spread of antimicrobial resistance in community-acquired infections [27,28]. Previous studies have suggested that these factors may create selective pressure that favours the survival and transmission of resistant bacterial strains. Addressing these drivers requires strengthening antimicrobial stewardship, improving access to diagnostic services to support targeted therapy, and enhancing surveillance of antimicrobial resistance to inform local treatment guidelines and public health interventions [29].

The antimicrobial resistance patterns observed in this study have important implications for empirical treatment of community-acquired urinary tract infections (CA-UTIs) in primary healthcare settings. In our cohort, *Escherichia coli* was the predominant uropathogens, and a large proportion of isolates exhibited multidrug resistance, with high resistance observed to commonly prescribed antibiotics including amoxicillin-clavulanic acid and ciprofloxacin. These findings suggest that some antibiotics frequently used for empirical management of urinary tract infections may have reduced effectiveness in this setting. Similar resistance patterns have been reported in Tanzania and other parts of East Africa, where increasing resistance among uropathogens has been associated with widespread empirical antibiotic use and limited diagnostic capacity [9,25,26]. Clinicians managing suspected urinary tract infections at primary healthcare facilities should therefore consider local antimicrobial susceptibility patterns when selecting empirical therapy. Strengthening routine surveillance of antimicrobial resistance and integrating these data into national and facility-level treatment guidelines are essential to ensure that empirical treatment remains effective in the context of rising antimicrobial resistance [30–32].

The prevalence of community-acquired, microbiologically confirmed UTI in the present study was 22%, which falls within the previously reported national range of 11–23% from diverse populations in Tanzania [5–7]. However, this prevalence is slightly lower than the 27% reported by Silago et al. in the same setting three years earlier [9]. This discrepancy may be explained by differences in study populations; the earlier study included pregnant women a group with a physiologically higher susceptibility to UTI while the current study excluded them.

From a public health perspective, a 22% prevalence indicates that UTIs remain a substantial community health burden, contributing to frequent outpatient visits and antimicrobial prescriptions. This underscores the need for strengthened community-level infection prevention strategies, including improved personal hygiene practices, safe water access, and health education aimed at early care seeking and avoidance of self-medication [33]. The high frequency of non-specific clinical presentations observed in this study highlights the need for future research evaluating the diagnostic performance of clinical symptoms against microbiological confirmation. Such evidence could improve symptom-based diagnosis and help optimize empirical antibiotic prescribing in primary healthcare settings. Point-of-care urine culture systems, dipstick testing, and clinical decision support tools could improve diagnostic accuracy and reduce inappropriate antibiotic use. Although only a minority of participants reported antibiotic use during the preceding 14 days, the high burden of resistance suggests ongoing antimicrobial pressure within the community. Such widespread community antibiotic exposure, often unregulated, can suppress bacterial growth and potentially lead to underestimation of true UTI prevalence while simultaneously exerting selection pressure that drives antimicrobial resistance (AMR).

In the current study, just like other studies, Gram-negative bacteria were predominant pathogens detected causing community-acquired UTI. The predominance of Gram-negative bacteria is partly contributed to by the fact that these bacteria are normal microbiota of the gastrointestinal tract, and the proximity of the urinary and gastrointestinal tract makes it easy for the organism to ascend and cause a urinary tract infection.

The predominance of Gram-negative bacteria, especially *E. coli*, is consistent with global and regional trends in community-acquired urinary tract infections (CA-UTIs), reflecting its well-established role as the primary uropathogens [9]. The relatively frequent recovery of *S. aureus* from urine cultures in this study is noteworthy because this organism is an uncommon cause of uncomplicated community-acquired urinary tract infection. Its isolation from midstream urine should therefore be interpreted cautiously, as it may represent contamination, colonization, complicated urinary tract infection, or less commonly haematogenous seeding. Nevertheless, the high prevalence of methicillin resistance and multidrug resistance among these isolates underscores the value of documenting *S. aureus* in community antimicrobial resistance surveillance. In this study, participants were enrolled based on symptoms suggestive of urinary tract infection at primary healthcare level, and detailed evaluation for systemic involvement or underlying comorbidities was not performed. However, identification of *S. aureus* was conducted using standard microbiological methods, making misidentification unlikely. The clinical significance of *S. aureus* isolated from urine in this outpatient setting should therefore be interpreted cautiously.

Differences in less common isolates between the two health centres may be linked to local antimicrobial use, patient demographics, or environmental factors influencing bacterial populations. Although *Citrobacter freundii* and *Streptococcus pyogenes* were isolated infrequently in this study, their detection remains clinically and epidemiologically relevant. *Citrobacter freundii* is an opportunistic pathogen capable of causing urinary tract infections and is increasingly recognized for harbouring clinically important antimicrobial resistance mechanisms, including inducible AmpC β-lactamases and extended-spectrum β-lactamases [34,35]. On the other hand, *Streptococcus pyogenes* is an uncommon uropathogen, sporadic urinary tract infections have been reported, particularly among individuals with predisposing host factors [36]. Owing to the small number of isolates recovered, no conclusions regarding their epidemiology or distribution between the study sites can be drawn. Nevertheless, their identification underscores the importance of continued microbiological surveillance for unusual or emerging uropathogens and their antimicrobial resistance patterns in community settings [37].

The high resistance observed, particularly among *E. coli* and non-fermentative Gram-negative bacteria, emphasizes the increasing challenge of treating CA-UTIs with commonly prescribed antibiotics [38]. These findings underscore the urgent need for ongoing surveillance, rational antimicrobial use, and updated treatment guidelines tailored to local resistance patterns to manage infections effectively and limit the spread of multidrug-resistant pathogens. The widespread multidrug resistance (MDR) observed in Gram-negative bacteria, with *E. coli* showing the highest levels, is particularly concerning, given its dominance as a uropathogens. Resistance to multiple antibiotic classes, including critical agents like piperacillin-tazobactam and amikacin, limits treatment options and reinforces the critical need for antimicrobial stewardship to address this growing threat.

The observed prevalence of ESBL-producing *Enterobacterales*, together with the high resistance to ceftriaxone among *E. coli* isolates, suggests reduced effectiveness of third-generation cephalosporins for empirical treatment of community-acquired urinary tract infections in this setting. These findings raise concern regarding the continued reliance on commonly prescribed beta-lactam antibiotics and highlight the importance of routine antimicrobial susceptibility surveillance to guide empirical therapy and support appropriate antibiotic selection at primary healthcare facilities.

The high MDR prevalence among *S. aureus* and other GPB in this study is consistent with findings by Silago et al. in Mwanza and Dar es Salaam, where similar resistance patterns to ciprofloxacin, tetracycline, and trimethoprim-sulfamethoxazole were observed [9]. Notably, the resistance profiles align with post-COVID-19 trends reported by the same group, showing persistent and widespread MDR uropathogens in Tanzania [39]. The significantly higher MDR burden at Igoma HC may reflect differences in local antimicrobial practices. These findings underscore the ongoing threat of resistant GPB pathogens and the urgent need for context-specific antimicrobial stewardship in outpatient settings. The resistance patterns observed in this study may therefore reflect ongoing community misuse or overuse of antibiotics previously reported [25,26], reinforcing the urgency of antimicrobial stewardship interventions, regulation of non-prescription antibiotic sales, and public education on the risks of inappropriate antibiotic use.

### Study limitations

This study has several limitations. First, it was conducted over a four-month period at two urban primary healthcare facilities, which may limit the generalizability of the findings to other settings and seasons in Tanzania. Second, the broad clinical case definition may have included some patients without true urinary tract infection, reflecting routine primary healthcare practice where laboratory confirmation is often unavailable. Third, prior antibiotic use was assessed only for the preceding 14 days, which may not fully capture earlier exposures relevant to antimicrobial resistance. Finally, the study did not evaluate the association between clinical symptoms and microbiological confirmation of urinary tract infection, limiting assessment of the diagnostic accuracy of symptom-based diagnosis.

## Conclusion, public health implication and recommendation

### Conclusion

This study revealed a significant burden of community-acquired urinary tract infections (CA-UTIs) among adult outpatients in Mwanza, Tanzania, with an overall prevalence of 22%. *Escherichia coli* and *Staphylococcus aureus* were the most frequently isolated pathogens, and alarmingly high levels of a resistance particularly multidrug resistance in *E. coli* were observed. The late presentation of patients, limited recognition of classical UTI symptoms, and high recurrence rates further complicate clinical management.

### Public health implications and recommendations

Following the findings of this study, strengthening routine local antimicrobial susceptibility surveillance is important to guide empirical treatment decisions for community-acquired urinary tract infections at primary healthcare facilities. The current empirical treatment guidelines may also require revision due to the high resistance observed to ciprofloxacin, trimethoprim-sulfamethoxazole, and amoxicillin-clavulanic acid among community uropathogens. In addition, improving access to urine dipstick testing, urine culture, and antimicrobial susceptibility testing could enhance diagnostic accuracy and reduce inappropriate antibiotic use in outpatient settings. Strengthening antimicrobial stewardship programs at primary healthcare facilities is also essential to promote rational antibiotic prescribing practices and support efforts to control antimicrobial resistance.

## Supporting information

S1 File Detailed antimicrobial susceptibility data. (XLSX)

S2 File Raw dataset. (XLSX)
