# Supplementary material for: Multidrug resistance in bacteria causing community-acquired urinary tract infections among adult outpatients attending primary healthcare facilities in Mwanza, Tanzania
Source: PLOS Glob Public Health. 2026 Oct 8;6(10):e0005638. doi: 10.1371/journal.pgph.0005638 (PMC13649018; doi:10.1371/journal.pgph.0005638)
Supplement: Supplementary file 2 [file pgph.0005638.s006.pdf]

PLOS Global Public Health

Multidrug resistance bacteria causing community acquired urinary tract infections among adult outpatients attending lower-level health facilities in Mwanza, Tanzania

--Manuscript Draft--

Manuscript Number: PGPH-D-25-03665R1

Article Type: Research Article

Full Title: Multidrug resistance in bacteria causing community acquired urinary tract infections among adult outpatients attending lower-level health facilities in Mwanza, Tanzania

Short Title: Multidrug resistance bacteria causing community acquired urinary tract infections

Corresponding Author: Martha F mushi, PhD
Catholic University of Health and Allied Sciences
Mwanza, TANZANIA, UNITED REPUBLIC OF

Order of Authors:
Eunice G. Emmanuel, MSc
Ashura Khamis, BSc
Elikana Michael, BSc
Dorina Muhizi, BSc
James Thomas, MSc
Bernard Okamo, MSc
Farida I. Mkassy, MSc
Eveline T. Konje, PhD
Vitus T. Silago, MSc
Martha F mushi, PhD
Stephen E. Mshana, PhD

Keywords: Community-acquired UTI; antimicrobial resistance; Escherichia coli; Multidrug resistance; Uropathogens; Outpatients; Tanzania

Abstract:

Background: Urinary tract infections (UTIs) remain a common clinical condition requiring antibiotic prescription among adult outpatients in low- and middle-income countries (LMICs). Data regarding the prevalence, patterns of bacteria and their antibacterial susceptibility profile for community acquired UTI are limited.
Methods: A hospital-based cross-sectional study was conducted from May to August 2024 at Igoma and Buzuruga Health Centres in Mwanza. Symptomatic adults' patients, without recent hospitalization (within past 30 days) and/or urinary catheterization were enrolled. Clinical and demographic data were collected followed by standard mid-stream urine culture and disc diffusion susceptibility testing. Descriptive data analysis was performed using STATA version 15.
Results: A total of 1,005 adult patients with the median age of 32 [IQR: 23–49] years were recruited, of whom 727 (72.3%) were female. The majority (64.3%) reported a previous history of UTI (within six months), and the median symptom duration before presentation was 8 [5–15] days. Microbiological confirmation of UTI was found in 221 patients (22.0%, 95% CI 19.5%-24.7%). The most frequently isolated uropathogens were Escherichia coli (32.9%) and Staphylococcus aureus (24.7%). Escherichia coli isolates were ≥50% resistant to ciprofloxacin, amoxicillin-clavulanic acid, trimethoprim-sulfamethoxazole, tetracycline, and ampicillin. About 20.3% of E. coli isolates showed positive extended-spectrum beta-lactamase (ESBL) phenotypes whereas 64.6% of S. aureus were resistant to cefoxitin, hence methicillin-resistant S. aureus (MRSA) strains.

Conclusion and recommendation: This study revealed a significant burden of community-acquired urinary tract infections (CA-UTIs) among adult outpatients, with an overall prevalence of 22%. Escherichia coli and Staphylococcus aureus were the most frequently isolated pathogens, ~~and~~ with alarmingly high levels of antimicrobial resistance, particularly multidrug resistance in E. coli. Improved diagnostic capacity and

Powered by Editorial Manager® and ProduXion Manager® from Aries Systems Corporation

	strengthened antibiotic stewardship are urgently needed to guide effective management of community-acquired UTIs to control the AMR development.<br>Highlight:<br><br>A total of 1,005 symptomatic adult patients at primary healthcare facilities were assessed for community-acquired urinary tract infections (CA-UTIs).<br><br>CA-UTIs were identified in 22% of cases, with Escherichia coli and Staphylococcus aureus as the most common pathogens.<br><br>E. coli demonstrated a notably high rate of multidrug resistance, with 82.8% of isolates resistant to multiple antibiotic classes.<br><br>Over 70% of Gram-negative bacterial isolates were resistant to at least three classes of antibiotics, presenting major treatment difficulties.<br><br>These results highlight the urgent need for ongoing surveillance and updated empirical treatment policies for UTIs in outpatient clinics, especially in resource-limited settings.
**Additional Information:**	
**Question**	**Response**
**Financial Disclosure**<br><br>Enter a financial disclosure statement that describes the sources of funding for the work included in this submission and the role the funder(s) played. This includes grants and any commercial funding of the work or authors.<br><br>This statement will be typeset if the manuscript is accepted for publication.<br><br>*Please review the submission guidelines and the instructions link below for detailed requirements and guidance.*	The authors received no specific funding for this work
**Competing Interests**<br><br>On behalf of all authors, disclose any competing interests that could be perceived to bias this work.	The authors have declared that no competing interests exist

Powered by Editorial Manager® and ProduXion Manager® from Aries Systems Corporation

This statement will be typeset if the manuscript is accepted for publication.<br><br>*Please review the instructions link below and PLOS Global Public Health's competing interests policy to determine what information must be disclosed at submission.*	
**Data Availability**<br><br>Before publication, Authors are required to make fully available and without restriction all data underlying their findings. Please see our PLOS Data Policy page for detailed information on this policy.<br><br>A **Data Availability Statement**, detailing where the data can be accessed, is required at first submission. Insert your Data Availability Statement in the box below.<br><br>Please see the data reporting section of our submission guidelines for instructions on what you need to include in your Data Availability Statement.<br><br>This statement will be typeset if the manuscript is accepted for publication.<br><br>*PLOS allows rare exemptions to address legal and ethical concerns. If you have legal or ethical restrictions, please use the box below to detail these in full sentences for the Journal team to consider.*	All relevant data are within the paper.

Manuscript
Click here to access/download;Manuscript;revise manuscript clear.docx

Multidrug resistance in bacteria causing community acquired urinary tract infections among adult outpatients attending primary healthcare facilities in Mwanza, Tanzania

Eunice G. Emmanuel[1,2], Ashura Khamis[1], Elikana Michael[1], Dorina Muhizi[1], James Thomas[1], Bernard Okamo[3], Farida I. Mkassy[4], Eveline T. Konje[4], Vitus Silago[5], Martha F. Mushi[5*], Stephen E. Mshana[5]

1. Weil Bugando School of Medicine, Catholic University of Health and Allied Sciences, P.O Box 1464, Mwanza, Tanzania
2. Pharmacy Department, Igoma Health Centre, Ilemela, Mwanza Tanzania
3. Department of Biochemistry and Molecular Biology Catholic University of Health and Allied Sciences, P.O Box 1464, Mwanza, Tanzania
4. Department of Epidemiology, Biostatistics and Behavioural science, School of Public Health, Catholic University of Health and Allied Sciences, P.O Box 1464, Mwanza, Tanzania
5. Department of Microbiology and immunology, Weil Bugando School of Medicine, Catholic University of Health and Allied Sciences, P.O Box 1464, Mwanza, Tanzania

Correspondence*: Martha F. Mushi, Email mushimartha@gmail.com

Abstract

Background: Urinary tract infections (UTIs) remain a common clinical condition requiring antibiotic prescription among adult outpatients in low- and middle-income countries (LMICs). Data regarding the prevalence, patterns of bacteria, and their antibacterial susceptibility profile for community-acquired UTI at primary healthcare facilities are limited, particularly in resource-constrained settings where empirical treatment contributes to increased antimicrobial resistance (AMR) and associated morbidity.

Methods: A primary healthcare facility-based cross-sectional study was conducted from May to August 2024 at Igoma and Buzuruga Health Centres in Mwanza. Symptomatic adult patients, without recent hospitalization (within the past 30 days) or urinary catheterization, were enrolled. Clinical and demographic data were collected, followed by standard mid-stream urine for culture and disc diffusion susceptibility testing. Descriptive data analysis was performed using STATA version 15.

Results: A total of 1,005 adult patients with a median age of 32 [IQR: 23–49] years were recruited, of whom 727 (72.3%) were female. The majority (64.3%) reported a previous history of UTI (within six months), and the median symptom duration before presentation was 8 [5–15] days. Microbiological confirmation of UTI was found in 221 patients (22.0%, 95% CI 19.5%-24.7%). The most frequently isolated uropathogens were *Escherichia coli* (32.9%) and *Staphylococcus aureus* (24.7%). Notably, only 35.7% of culture-confirmed UTI patients presented with classic urinary symptoms (dysuria, frequency, urgency), highlighting clinical diagnostic challenges in this setting. *E. coli* isolates demonstrated alarmingly high resistance rates: ciprofloxacin (62.3%), trimethoprim-sulfamethoxazole (60.7%), amoxicillin-clavulanic acid (57.4%), tetracycline (55.7%), and ampicillin (93.4%). Critically, 50% of *E. coli* isolates exhibited multidrug resistance (MDR), and 20.3% showed extended-spectrum beta-lactamase (ESBL) phenotypes. Among *S. aureus* isolates, 64.6% were resistant to cefoxitin, indicating methicillin-resistant *S. aureus* (MRSA) strains.

Conclusion and recommendation: This study revealed a significant burden of community-acquired urinary tract infections (CA-UTIs) among adult outpatients at primary healthcare facilities, with an overall

prevalence of 22%. *E. coli* and *S. aureus* were the most frequently isolated pathogens, with alarmingly high levels of antimicrobial resistance, particularly multidrug resistance in *E. coli*. The high prevalence of atypical clinical presentations and the failure of commonly used first-line antibiotics (ciprofloxacin, trimethoprim-sulfamethoxazole, amoxicillin-clavulanic acid) underscore the urgent need for improved diagnostic capacity, strengthened antibiotic stewardship programs, and updated treatment guidelines tailored to local resistance patterns to guide effective management of community-acquired UTIs and control AMR development.

Key words: Community-acquired UTI; Antimicrobial resistance; *Escherichia coli*; Multidrug resistance; Uropathogens; Outpatients; Tanzania

Highlight:

- A total of 1,005 symptomatic adult patients at primary healthcare facilities were assessed for community-acquired urinary tract infections (CA-UTIs).
- CA-UTIs were identified in 22% of cases, with *Escherichia coli* and *Staphylococcus aureus* as the most common pathogens.
- *E. coli* demonstrated a notably high rate of multidrug resistance, with about 50% of isolates resistant to multiple antibiotic classes.
- Over 70% of Gram-negative bacterial isolates were resistant to at least three classes of antibiotics, presenting major treatment difficulties.
- These results highlight the urgent need for ongoing surveillance and updated empirical treatment policies for UTIs in outpatient clinics, especially in resource-limited settings.

Background

Urinary tract infections (UTIs) are a prevalent health concern among patients visiting outpatient clinics in low- and middle-income countries (LMICs). In these regions, UTIs significantly contribute to morbidity, elevated treatment expenses, and mortality, especially among those seeking care at primary health care facilities [1, 2]. Limited diagnostic resources coupled with insufficient microbiological evidence often compromise effective patient management. However, UTI remains one of the most common reasons for antibiotic prescription and the most frequent reason for hospital attendance among children and adults in Tanzania, surpassed only by malaria [3, 4]. In Tanzania, the prevalence of UTI is reported to be different among populations, ranging from 11-23% [5-7]. A previous study conducted in lower health facilities in among patients of all age groups reported the culture-proven prevalence of 27.4% [8, 9].

Gram-negative bacteria in the order Enterobacterales, such as *E. coli* and *Klebsiella pneumoniae,* are the most common uropathogens associated with both community and health care settings, UTIs [1, 9]. These isolates have been found to produce extended-spectrum beta-lactamases(ESBL). Among Gram-positive bacteria, *Staphylococcus aureus* has been reported as the leading pathogen [10]. In LMICs, studies have documented enzyme production, such as extended-spectrum beta-lactamase (ESBL), as the main mechanism of resistance among Gram-negative bacteria causing UTIs [11, 12]. Previous studies conducted in Tanzania reported the prevalence of multidrug-resistant (MDR) bacteria causing UTIs to range from 22.4%-60.3% among Gram-positive bacteria and 45.4%-52.2% among Gram-negative bacteria [4, 9, 13, 14].

Several factors increase the risk of community-acquired urinary tract infections (CA-UTIs). Demographic and physiological factors are significant, with women being more vulnerable due to their shorter urethra and its proximity to the anus. Older adults are at greater risk due to weakened immune defences and underlying health conditions, while pregnancy increases susceptibility because of hormonal changes and pressure on the bladder[15]. Behavioural and lifestyle factors, such as poor hygiene, frequent sexual

activity, and the use of spermicides or diaphragms, can disrupt the natural bacterial balance in the urinary tract, further increasing the likelihood of infection.

Additionally, certain medical conditions and structural abnormalities heighten the risk. Individuals with diabetes mellitus are more prone to UTIs due to compromised immune function and elevated glucose levels that encourage bacterial growth. Conditions such as kidney stones, congenital abnormalities, or an enlarged prostate can obstruct urine flow, fostering an environment for bacterial proliferation. Medications and medical interventions are other contributing factors, as the use of antibiotics can disturb the balance of normal flora, and temporary catheterization, even at home, can increase the likelihood of bacterial colonization[4]. Furthermore, antimicrobial resistance patterns vary significantly by geographic location and change over time, making local surveillance data essential for guiding empirical treatment decisions.

Previous studies in Tanzania have primarily focused on tertiary hospitals or specific populations such as pregnant women, children, or people living with HIV[4-7, 9, 13, 14] leaving a significant knowledge gap regarding CA-UTIs among general adult outpatients at primary healthcare facilities. Understanding the prevalence, causative organisms, and resistance patterns in this setting is essential for developing targeted interventions, updating treatment guidelines, and implementing effective antimicrobial stewardship programs.

This study aimed to determine the prevalence of CA-UTIs, identify the causative bacterial pathogens, and characterize their antimicrobial susceptibility profiles among adult outpatients attending primary healthcare facilities in Mwanza, Tanzania. By focusing on the primary care setting where most patients first present, this research provides critical insights into the current state of AMR in community settings and informs public health strategies to combat the rising threat of multidrug-resistant infections.

Methodology

Study design and settings: This was a primary healthcare facility-based cross-sectional study conducted from 15th May to 30th August 2024, at Igoma and Buzuruga Health Centre in Mwanza, Tanzania. Buzuruga

Health Centre serves up to 2,266 patients per month, while Igoma Health Centre attends approximately 1,500 outpatients monthly.

Sample size and study population: The study population comprised adult outpatients (≥18 years) presenting with symptoms suggestive of UTI. According to established clinical guidelines[16], Participants were included when presenting with at least two of the following symptoms: dysuria, urinary frequency, urinary urgency, suprapubic pain, or lower abdominal pain. The minimum sample size was obtained by the Kish-Leslie formula [17] using a 24.7% prevalence of CA-UTIs previously reported by Silago et *al*.[9]. To account for potential non-response and ensure adequate power for subgroup analyses, the sample was increased, resulting in a target enrollment of 1,005 participants. Pregnant women, patients with a history of admission within 30 days on the day of sample collection, and those with an indwelling urinary catheter at the time of study were excluded.

Data and sample collections: Demographic and clinical data were collected using Epicollect5, a mobile data-gathering platform developed by the CGPS Team of Oxford BDI and publicly available at https://five.epicollect.net. Information on prior antibiotic use was collected based on recent self-reported use to minimize recall bias and improve data reliability. About 3-5 ml of clean catch midstream urine was collected by the patients after receiving instructions from the research assistant, using a sterile, wide-mouthed, labelled container. The urine sample was transported to the microbiology research laboratory of CUHAS for processing within 2 hours of collection. For women, urine samples were tested for HCG to exclude pregnancy[18]. Urine was divided into two portions for microscopy/dipstick analysis and culture. All laboratory data were documented on the laboratory logbook.

Urine Culture: Urine samples were inoculated onto agar media (Blood Agar, MacConkey Agar and CLED) and incubated at 37°C for 18-24 hours. Growth estimate of ≥1000 colonies from 10µl inoculating loop for both men and women was considered as significant bacteriuria and thus UTI [19]. In-house-prepared conventional biochemical identification tests including: catalase, coagulase, oxidase, indole, citrate, urease, and sugar fermentation tests were used for the preliminary identification of bacteria isolates to

their possible species levels as previously described [20]. Gram-positive and Gram-negative bacteria were identified using morphological and biochemical tests as previously described [21]. Unidentified isolates were further analysed using Matrix-Assisted Laser Desorption Ionization–Time of Flight (MALDI-TOF) mass spectrometry at the National Public Health Laboratory (NPHL)[22]. Antimicrobial susceptibility testing was performed as per CLSI guidelines [23].

Quality Control: Quality assurance measures encompassed monitoring reagent storage, equipment functionality, media sterility, and preparation of standardized bacterial suspensions. All stains and reagents were systematically labelled, dated, and stored under appropriate conditions. Refrigerator and incubator temperatures were recorded and tracked daily to ensure compliance with operational standards. Culture media were prepared in strict accordance with manufacturer instructions and evaluated for both performance and sterility. To maintain consistent inoculum density for susceptibility testing, a 0.5 McFarland standard was utilized; standard reference strains like *S. aureus* (ATCC 25923), *MRSA* (ATCC 29213), *E. coli* (ATCC 25922), ESBL-producing *Klebsiella pneumoniae* (ATCC 700603), and *Pseudomonas aeruginosa* (ATCC 27853) were employed as controls. Data cleaning and coding were conducted using STATA version 15, with records containing missing values excluded from analysis.

Data Analysis: Data were analysed using STATA software version 15 in line with the study's objectives. Descriptive statistics were used to summarize data, with continuous variables presented as medians with interquartile ranges and categorical variables as frequencies and proportions.

Ethical Considerations: Ethical clearance was obtained from the Joint CUHAS/BMC Research Ethics and Review Committee (CREC) with certificate number CREC No 763/2024. All participants were requested to consent by signing written informed consent form. Privacy and confidentiality were maintained throughout the study. The urine culture results were timely shared to the manage clinicians to guide the management.

Results

The study recruited a total of 1005 adult, patients with a clinical diagnosis of UTI. In general, majority of recruited patients were non-pregnant women 727(72.3%), about two third were married 610 (60.7%) and their median age was 32[23-49] years, as detailed in table1.

Regarding clinical history, 646 participants (64.3%) reported a previous episode of UTI within the past six months, and 129 (12.8%) had used antibiotics within the 14 days preceding enrollment. The median duration of symptoms before seeking care was 8 days (IQR: 5-15 days). Notably, only 359 participants (35.7%) presented with classic urinary tract symptoms (dysuria, frequency, and urgency), while the majority presented with non-specific symptoms such as lower abdominal pain, fever, or lower back pain, highlighting the clinical diagnostic challenges in this setting. Patients recruited from Igoma health centre reported late to the health facility than those from Buzuruga health centre (14[7-28] vs. 7[3-14] days), P=0.001. as indicated in table 1.

Table 1: Demographic and clinical characteristics of the studied population

Variable		Buzuruga HC (566) n(%)	Igoma HC (439) n(%)	Total (1005)
Gender	Male	138(24.4)	140(31.9)	278(27.7)
	Female	428(75.6)	299(68.1)	727(72.3)
Age	Median [IQR]	32[23-50]	30[22-49]	32[23-49]
Education	No formal education	37(6.5)	65(14.8)	102(10.2)
	Primary	243(42.9)	216(49.2)	459(45.7)
	Secondary	220(38.9)	123(28.0)	343(34.1)
	University	66(11.7)	35(7.9)	101(10.1)
Marital status	Married	367(64.8)	243(55.4)	610(60.7)
	Single	199(35.2)	196(44.6)	395(39.3)
History of UTI	Yes	382(67.5)	264(60.1)	646(64.3)
	No	184(32.5)	175(39.9)	359(35.7)
Dysuria	Yes	137(24.2)	117(26.6)	254(25.3)
	No	429(75.8)	322(73.4)	751(74.7)
Urgency	Yes	54(9.5)	16(3.6)	70(6.9)
	No	512(90.5)	423(96.4)	935(93.0)
Frequency	Yes	66(11.7)	40(9.1)	106(10.6)
	No	500(88.3)	399(90.9)	899(89.4)
Nocturia	Yes	20(3.5)	22(5.0)	42(4.2)
	No	546(96.5)	417(94.9)	963(95.8)
Cloudy urine	Yes	58(10.3)	31(7.1)	89(8.9)
	No	508(89.7)	408(92.9)	916(91.1)
~~Smelling~~ urine Smelly	Yes	40(7.1)	14(3.2)	54(5.4)
	No	526(92.9)	425(96.8)	951(94.6)
Lower abdominal pain	Yes	275(48.6)	237(53.9)	512(50.9)
	No	291(51.4)	202(46.0)	493(49.1)
Lower back pain	Yes	161(28.5)	212(48.3)	373(37.1)
	No	405(71.5)	227(51.7)	632(62.9)
Fever	Yes	377(66.6)	211(48.1)	588(58.5)
	No	189(33.4)	228(51.9)	417(41.5)
Duration of symptoms (days)		7[3-14]	14[7-28]	8[5-15]
Presence of comorbidity	Yes	96(16.9)	26(5.9)	122(12.1)
	No	470(83.0)	413(94.1)	883(87.9)
Current antibiotic use	Yes	89(15.7)	40(9.1)	129(12.8)
	No	477(84.3)	399(90.9)	876(87.2)
Co-morbidity	High blood Pressure	26(27.1%)	12(46.2%)	38(31.1%)
	Diabetic Mellitus	19(19.8%)	6(23.1%)	25(20.5%)
	Chronic Back Pain	19(19.8%)	4(15.4%)	23(18.9%)
	Peptic Ulcer Diseases	23(23.9%)	0(0.0%)	23(18.9%)
	Others	9(9.4%)	4(15.4%)	13(10.7%)

Others included Benign prostatic hyperplasia 2; living with HIV 6; chronic leg pain 1; ovarian cyst 4

Among the recruited patients with other comorbidities (n=122) ~~122~~, high blood pressure was the commonest, 38 (31.1%). A total of seven patients had more than one chronic condition (comorbidity) such as diabetic mellitus and hypertension (six patients), and one patient had both hypertension and peptic ulcer diseases making total count of 122.

Prevalence of community acquired UTI

Of the 1005 recruited patients with clinical presentation of urinary tract infections, 221(22%), 95% CI 19.4%-24.6% were microbiologically confirmed. Igoma health centre had 22.8%, (n=100/439), 95% CI 18.9%-27% patients with microbiologically confirmed UTI while Buzuruga health centre had 21.4%, (n=121/566), 95% CI 18%-25% patients.

Proportions of bacterial species causing CA-UTIs

Of the 221 microbiologically confirmed cases, 194 were due to bacterial isolates, while 27 showed significant growth of *Candida* spp. The majority of bacteria isolated were Gram-negative (59.8%; n = 116). Overall, the most frequently detected bacterial species were *E. coli* (32.9%; n = 64) and *S. aureus* (24.7%; n = 48). A similar pattern of predominant bacterial pathogens was observed at both Buzuruga Health Centre and Igoma Health Centre, however, the third and fourth most common isolates differed slightly between the two sites. At Buzuruga HC, *Enterococcus* spp. (10.5%; n = 11) and *Acinetobacter* spp. (9.5%; n = 10) were the commonest third and fourth pathogen, respectively while at Igoma HC *Acinetobacter* spp. (10.1%; n = 9) and *K. pneumoniae* complex (8.9%; n = 8) were common. Notably, all *C. freundii* strains were exclusively isolated at Buzuruga HC (Table 2, Figure 1).

Table 2: The proportions of bacterial species causing community-acquired urinary tract infections

Isolate	All (n=194)	Buzuruga HC (N=105)	Igoma HC (N=89)
	n(%)	n(%)	n(%)
E. coli	64 (32.9)	33 (31.4)	31 (34.8)
S. aureus	48 (24.7)	25 (23.8)	23 (25.8)
Acinetobacter spp.	19 (9.8)	10 (9.5)	9 (10.1)
Enterococcus spp.	16 (8.2)	11 (10.5)	5 (5.6)
K. pneumoniae complex	12 (6.2)	4 (3.8)	8 (8.9)
~~*S. pyogenes*~~	10 (5.2)	5 (4.8)	5(5.6)
P. aeruginosa	7 (3.6)	2 (1.9)	5 (5.6)

C. freundii	5 (2.6)	5 (4.8)	0 (0.0)
Others*	13 (6.7)	10 (9.5)	3 (3.4)

***Others: n=13:** *Morganella morganii* (n=3), *Enterobacter cloacae* complex (n=2), *Citrobacter koseri* (n=1), *Klebsiella oxytoca* (n=1), *Pseudomonas fluorescens* (n=1), *Proteus mirabilis* (n=1), *Pseudoglutamicibacter cumminsii* (n=1), *Streptococcus agalactiae* (n=1), *Staphylococcus epidermidis* (n=1), and *Staphylococcus haemolyticus* (n=1).

Buzuruga HC (n=10): *Morganella morganii* (n=3), *Enterobacter cloacae* complex (n=1), *Citrobacter koseri* (n=1), *Klebsiella oxytoca* (n=1), *Pseudomonas fluorescens* (n=1), *Pseudoglutamicibacter cumminsii* (n=1), *Staphylococcus epidermidis* (n=1), and *Staphylococcus haemolyticus* (n=1).

Igoma HC (n=3): *Enterobacter cloacae* complex (n=1), *Proteus mirabilis* (n=1), and *Streptococcus agalactiae* (n=1).

Percentage resistance of bacterial pathogens causing CA-UTIs

Escherichia coli demonstrated the highest levels of antimicrobial resistance (≥50%) to multiple antibiotics, including ciprofloxacin, amoxicillin-clavulanic acid, trimethoprim-sulfamethoxazole, tetracycline, and ampicillin. About 20.3% of *E. coli* isolates showed positive phenotypes of extended-spectrum beta-lactamase (ESBL) production. Other members of the Enterobacterales group showed similarly high resistance rates to amoxicillin-clavulanic acid and ampicillin, with 12.0% detected with ESBL phenotype. The non-fermentative Gram-negative bacteria, specifically *Acinetobacter* spp. and *Pseudomonas* spp., exhibited the highest resistance to amoxicillin-clavulanic acid, trimethoprim-sulfamethoxazole, ceftriaxone, and ampicillin (Table 3). On the other hand, *S. aureus* demonstrated markedly high resistance rates (≥ 50%) to ciprofloxacin, tetracycline, trimethoprim-sulfamethoxazole, ampicillin, and erythromycin. Moreover, 64.6% of *S. aureus* strains were resistant to cefoxitin, hence methicillin-resistant *S. aureus* (MRSA). Similarly, other Gram-negative bacteria exhibited peak resistance (≥ 50%) to ampicillin, tetracycline, trimethoprim-sulfamethoxazole, ciprofloxacin, and erythromycin (Table 3).

Table 3: Percentage resistance of bacteria causing community-acquired urinary tract infection

	Gram negative bacteria					
Antibiotic agents tested	*E. coli* (N=64)		Other Enterobacterales (N=25)		Non-fermentative Gram-negative bacteria (N=27)	
	% Resistance	% Sensitive	% Resistance	% Sensitive	% Resistance	% Sensitive
AMP	58 (90.6)	6 (9.4)	25 (100)	0 (0.0)	5 (100)	0 (0.0)
TET	47 (73.4)	17 (26.6)	10 (40.0)	15 (60.0)	11 (47.8)	12 (52.2)
SXT	46 (73.0)	17 (27.0)	10 (40.0)	15 (60.0)	NA	
CIP	34 (53.1)	30 (46.9)	8 (32.0)	17 (68.0)	9 (33.3)	18 (66.7)
NIT	26 (40.6)	38 (59.4)	14 (56.0)	11 (44.0)	25 (92.6)	2 (7.4)
AMC	41 (64.1)	23 (35.9)	13 (52.0)	12 (48.0)	NA	
CRO	29 (45.3)	35 (54.7)	11 (44.0)	14 (56.00)	NA	
GEN	29 (45.3)	35 (54.7)	6 (25.0)	18 (75.0)	4 (15.4)	22 (86.4)
MEM	5 (7.9)	58 (92.1)	4 (16.0)	21 (84.0)	0 (0.0)	26 (100)
ESBL strains	13 (20.3)		3 (12.0)		NA	
Antibiotic agents tested	***Gram positive bacteria***					
	S. aureus (N=48)		Other Gram-positive bacteria (N=29)			
	% Resistance	% Sensitive	% Resistance		% Sensitive	
ER	NA		28 (96.6)		1 (3.4)	
CIP	24 (50.0)	24 (50.0)	25 (86.2)		4 (13.8)	
SXT	31 (64.6)	17 (35.4)	24 (82.8)		5 (17.2)	
TET	28 (58.4)	20 (41.6)	22 (75.9)		7 (24.1)	
PEN	38 (79.2)	10 (20.8)	15 (51.7)		14 (48.3)	
CD	29 (31.3)	33 (68.7)	14 (48.3)		15 (51.7)	
GEN	11 (22.9)	37 (77.1)	11 (37.9)		18 (62.1)	
NIT	12 (25.0)	36 (75.0)	11 (37.9)		18 (62.1)	
FOX	31 (64.6)	17 (35.4)	NT		NT	

Key: AMP=ampicillin, TET=tetracycline, SXT=trimethoprim-sulfamethoxazole, CIP=ciprofloxacin, NIT=nitrofurantoin, AMC=amoxicillin-clavulanic acid, CRO=ceftriaxone, GEN=gentamicin, NA=not applicable, and MEM=meropenem; ER=erythromycin; CIP=ciprofloxacin; SXT=trimethoprim-sulfamethoxazole; TET=tetracycline; PEN=penicillin; CD=clindamycin; GEN=gentamicin; NIT-nitrofurantoin; and FOX=cefoxitin.

Proportions of multidrug resistance and resistance patterns of Gram-negative bacteria causing CA-UTIs

Overall, the majority of Gram-negative bacteria (73.3%; 85/116) exhibited resistance to at least three classes of antimicrobials, with the number of classes ranging from three to six. By healthcare facility, the difference in proportions of MDR in Gram-negative bacteria isolated from patients attending ~~at~~ Buzuruga HC (70.5%; 43/61) and Igoma HC (76.4%; 42/55) was not statistically significant (p=0.475). *E. coli* showed the highest proportion of multidrug resistance (MDR), with 82.8% (53/64) of isolates classified as MDR, including 15.1% (n = 8) that were resistant to six antimicrobial classes. The common resistance patterns

in *E. coli* were AMP-TET-SXT-CIP-NIT-AMC-CRO-CAZ-FEP-GEN-AK-TZP (n=3), AMP-TET-SXT-CIP-NIT-AMC-CRO-CAZ-FEP-GEN-TZP (n=2), and AMP-TET-SXT-CIP-AMC-CRO-CAZ-FEP-AK-TZP (n=2). Among other Enterobacterales, 44.0% (11/25) were MDR, with 27.3% (n = 3) also resistant to six classes. Similarly, 77.8% (22/27) of non-fermentative Gram-negative bacteria displayed MDR, with 9.1% (n = 2) resistant to six antimicrobial classes. The common resistance patterns in non-fermentative Gram-negative bacteria were SXT-NIT-CRO-CAZ-FEP (n=2) and TET-SXT-NIT-CRO-FEP-TZP (n=2) (Table 3). Detailed antimicrobial resistance patterns are provided in Supplementary File 1.

Among GPB, a substantial proportion of *S. aureus* isolates (83.3%; 40/48) were classified as MDR. Notably, 15.0% (6/40) of these MDR *S. aureus* strains exhibited resistance to as many as seven antibiotics. MDR phenotypes were significantly more prevalent among *S. aureus* isolates from patients at Igoma Health Centre compared to those from Buzuruga HC (95.6% vs. 72.0%; p = 0.019). The most frequently observed resistance patterns in *S. aureus* included CD-ER-AMP-SXT-TET-CIP-FOX (n=3), ER-AMP-SXT-TET-CIP-FOX (n=3), and ER-AMP-SXT-TET-NIT-CIP-FOX-GEN (n=3). For other GPB, all isolates (100%; 29/29) were MDR. The most common resistance profiles among these included CD-ER-AMP-SXT-TET-CIP (n=2), CD-ER-NIT-CIP (n=2), and ER-AMP-SXT-TET-NIT-CIP (n=2). Moreover, 24.1% (7/29) of these MDR GPB strains demonstrated resistance to six antimicrobial agents (Table 4). Detailed antimicrobial resistance patterns are shown on Supplementary file 2.

Table 4: Distribution of multidrug resistance and number of resistant antibiotic classes among bacterial isolates causing urinary tract infections in primary healthcare facilities

Variable	Category	*E. coli* (N=64)	Other Enterobacterales (N=25)	Non-fermentative GNB (N=27)
		n(%)	n(%)	n(%)
Multidrug-resistant (MDR) strain	Yes	53 (82.8)	11 (44.0)	21 (77.8)
	No	11 (17.2)	14 (56.0)	6 (22.2)
The number of antibiotic classes to which the bacteria resist	3	10 (18.9)	2 (18.2)	10 (45.4)
	4	13 (24.5)	4 (36.4)	6 (27.3)
	5	22 (41.5)	2 (18.2)	3 (13.6)
	6	8 (15.1)	3 (27.3)	2 (9.1)
Health Centre for isolation of MDR strains	Buzuruga HC (N=43)	28 (65.1)	9 (20.9)	6 (13.9)
	Igoma HC (N=42)	25 (59.5)	12 (28.6)	5 (11.9)

Variable	Category	*S. aureus* (N=48)	Other Gram-positive bacteria (N=29)
		n(%)	n(%)
Multidrug-resistant (MDR) strain	Yes	40 (83.3)	29 (100)
	No	8 (16.7)	0 (0.0)
The number of antibiotic classes to which the bacteria resist	3	9 (22.5)	6 (20.7)
	4	11 (27.5)	8 (27.6)
	5	12 (30.0)	8 (27.6)
	6	2 (5.0)	7 (24.1)
	7	6 (15.0)	0 (0.0)
Health centre for isolation of MDR strains	Buzuruga HC	18/25 (72.0)	18/18 (100)
	Igoma HC	22/23 (95.6)	11/11 (100)

Discussion

This study provides important insights into the bacterial causes and antimicrobial resistance patterns of community-acquired urinary tract infections among patients attending primary healthcare facilities in Mwanza, Tanzania. The findings demonstrate that *Escherichia coli* remains the predominant uropathogens, with a substantial proportion of isolates exhibiting multidrug resistance. Notably, high resistance was observed to several commonly used antibiotics, raising concerns about the effectiveness of current empirical treatment practices in primary healthcare settings. Although only a small proportion of participants reported prior antibiotic use, the observed resistance patterns suggest ongoing antimicrobial pressure within the community. Together, these findings highlight the growing public health

challenge of antimicrobial resistance in community-acquired infections and underscore the need for strengthened surveillance, improved diagnostic capacity, and evidence-based antimicrobial stewardship to guide appropriate treatment.

The high prevalence of multidrug-resistant uropathogens observed in this study likely reflects multiple drivers operating at the community and healthcare levels. In many low- and middle-income countries, including Tanzania, antibiotics are frequently used empirically for urinary tract infections due to limited access to microbiological diagnostic services at primary healthcare facilities [24, 25]. In addition, inappropriate antibiotic use, including self-medication, incomplete treatment courses, and over-the-counter access to antimicrobials, has been reported as a major contributor to the emergence and spread of antimicrobial resistance in community-acquired infections[26, 27]. These factors create selective pressure that favours the survival and transmission of resistant bacterial strains. Addressing these drivers requires strengthening antimicrobial stewardship, improving access to diagnostic services to support targeted therapy, and enhancing surveillance of antimicrobial resistance to inform local treatment guidelines and public health interventions[28].

The antimicrobial resistance patterns observed in this study have important implications for empirical treatment of community-acquired urinary tract infections (CAUTIs) in primary healthcare settings. In our cohort, *Escherichia coli* was the predominant uropathogens, and a large proportion of isolates exhibited multidrug resistance, with high resistance observed to commonly prescribed antibiotics including amoxicillin-clavulanic acid and ciprofloxacin. These findings suggest that some antibiotics frequently used for empirical management of urinary tract infections may have reduced effectiveness in this setting. Similar resistance patterns have been reported in Tanzania and other parts of East Africa, where increasing resistance among uropathogens has been associated with widespread empirical antibiotic use and limited diagnostic capacity[9, 24, 25]. Clinicians managing suspected urinary tract infections at primary healthcare facilities should therefore consider local antimicrobial susceptibility patterns when selecting empirical therapy. Strengthening routine surveillance of antimicrobial resistance and integrating these data into

national and facility-level treatment guidelines are essential to ensure that empirical treatment remains effective in the context of rising antimicrobial resistance [29-31].

The prevalence of community-acquired, microbiologically confirmed UTI in the present study was 22%, which falls within the previously reported national range of 11–23% from diverse populations in Tanzania [5-7]. However, this prevalence is slightly lower than the 27% reported by Silago et al. in the same setting three years earlier [9]. This discrepancy may be explained by differences in study populations; the earlier study included pregnant women a group with a physiologically higher susceptibility to UTI while the current study excluded them.

From a public health perspective, a 22% prevalence indicates that UTIs remain a substantial community health burden, contributing to frequent outpatient visits and antimicrobial prescriptions. This underscores the need for strengthened community-level infection prevention strategies, including improved personal hygiene practices, safe water access, and health education aimed at early care seeking and avoidance of self-medication[32]. The high proportion of atypical presentations highlights the need for improved diagnostic capacity at primary care facilities. Point-of-care urine culture systems, dipstick testing, and clinical decision support tools could improve diagnostic accuracy and reduce inappropriate antibiotic use.

Importantly, most participants reported prior antibiotic use, a finding with significant implications. Such widespread community antibiotic exposure, often unregulated, can suppress bacterial growth and potentially lead to underestimation of true UTI prevalence while simultaneously exerting selection pressure that drives antimicrobial resistance (AMR).

In the current study, just like other studies, Gram-negative bacteria were predominant pathogens detected causing community-acquired UTI. The predominance of Gram-negative bacteria is partly contributed to by the fact that these bacteria are normal microbiota of the gastrointestinal tract, and the close proximity of the urinary and gastrointestinal tract makes it easy for the organism to ascend and cause a urinary tract infection.

The predominance of Gram-negative bacteria, especially *E. coli*, is consistent with global and regional trends in community-acquired urinary tract infections (CA-UTIs), reflecting its well-established role as the primary uropathogens [9]. The notable presence of *S. aureus*, a Gram-positive organism, suggests potential shifts or local variations in CA-UTI causes, warranting further exploration. *Staphylococcus aureus* is an uncommon cause of uncomplicated urinary tract infections and is more frequently associated with complicated infections or hematogenous spread. In this study, participants were enrolled based on symptoms suggestive of urinary tract infection at primary healthcare level, and detailed evaluation for systemic involvement or underlying comorbidities was not performed. However, identification of *S. aureus* was conducted using standard microbiological methods, making misidentification unlikely. The clinical significance of *S. aureus* isolated from urine in this context should therefore not underestimated. Differences in less common isolates between the two health centres may be linked to local antimicrobial use, patient demographics, or environmental factors influencing bacterial populations. The exclusive detection of *C. freundii* at one site highlights potential micro-epidemiological differences, which could inform more targeted infection control and surveillance efforts. Although *Streptococcus pyogenes* is not a typical uropathogens, occasional cases have been reported, particularly in association with perineal colonization or ascending infection from the genital tract[33]. In this study, the organism was rarely isolated and its clinical significance should therefore be interpreted cautiously.

The high resistance observed, particularly among *E. coli* and non-fermentative Gram-negative bacteria, emphasizes the increasing challenge of treating CA-UTIs with commonly prescribed antibiotics [34]. These findings underscore the urgent need for ongoing surveillance, rational antimicrobial use, and updated treatment guidelines tailored to local resistance patterns to manage infections effectively and limit the spread of multidrug-resistant pathogens. The widespread multidrug resistance (MDR) observed in Gram-negative bacteria, with *E. coli* showing the highest levels, is particularly concerning, given its dominance as a uropathogens. Resistance to multiple antibiotic classes, including critical agents like piperacillin-

tazobactam and amikacin, limits treatment options and reinforces the critical need for antimicrobial stewardship to address this growing threat.

The high MDR prevalence among *S. aureus* and other GPB in this study is consistent with findings by Silago et al. in Mwanza and Dar es Salaam, where similar resistance patterns to ciprofloxacin, tetracycline, and trimethoprim-sulfamethoxazole were observed [9]. Notably, the resistance profiles align with post-COVID-19 trends reported by the same group, showing persistent and widespread MDR uropathogens in Tanzania [35]. The significantly higher MDR burden at Igoma HC may reflect differences in local antimicrobial practices. These findings underscore the ongoing threat of resistant GPB pathogens and the urgent need for context-specific antimicrobial stewardship in outpatient settings. The resistance patterns observed in this study may therefore reflect ongoing community misuse or overuse of antibiotics previously reported [24, 25], reinforcing the urgency of antimicrobial stewardship interventions, regulation of non-prescription antibiotic sales, and public education on the risks of inappropriate antibiotic use.

Study limitations

Several limitations should be considered when interpreting these findings. The study was conducted over a relatively short period (4 months) during the dry season, and seasonal variations in UTI prevalence and etiology may exist. The study was limited to two primary healthcare facilities in an urban/peri-urban setting, and findings may not be generalizable to rural areas or other regions of Tanzania. The broad case definition for UTI symptoms may have included some patients without true UTI, though this reflects real-world primary care practice, where laboratory confirmation is often unavailable. Finally, information on antibiotic use was limited to the 14 days preceding enrollment, while longer-term antibiotic exposure (e.g., 90 days) may be more relevant for AMR risk assessment.

Conclusion, public health implication and Recommendation

Conclusion: This study revealed a significant burden of community-acquired urinary tract infections (CA-UTIs) among adult outpatients in Mwanza, Tanzania, with an overall prevalence of 22%. *Escherichia coli*

and *Staphylococcus aureus* were the most frequently isolated pathogens, and alarmingly high levels of a resistance particularly multidrug resistance in *E. coli* were observed. The late presentation of patients, limited recognition of classical UTI symptoms, and high recurrence rates further complicate clinical management.

Public health implications and recommendations

Urgent need for updated treatment guidelines: Current empirical treatment recommendations for CA-UTI in Tanzania are no longer supported by local susceptibility data. Treatment guidelines should be updated to reflect local resistance patterns, with consideration of alternative first-line agents such as nitrofurantoin for uncomplicated cystitis (where *E. coli* susceptibility remains 83.6%) or gentamicin for more severe infections, while reserving carbapenems for complicated cases or confirmed ESBL infections.

Strengthening antimicrobial stewardship: Comprehensive antimicrobial stewardship programs should be implemented at primary healthcare facilities, including: - Improved access to rapid diagnostic tests to guide targeted therapy, training of healthcare workers on appropriate antibiotic prescribing, public education campaigns on responsible antibiotic use, regulation of over-the-counter antibiotic sales, and monitoring and surveillance of antibiotic consumption and resistance patterns

Enhanced diagnostic capacity: The high proportion of atypical presentations highlights the need for improved diagnostic capacity at primary care facilities. Point-of-care urine culture systems, dipstick testing, and clinical decision support tools could improve diagnostic accuracy and reduce inappropriate antibiotic use.

Addressing drivers of AMR: Interventions must address the underlying drivers of AMR in Tanzania, including: - Poverty and limited healthcare access leading to self-medication - Inadequate water, sanitation, and hygiene (WASH) infrastructure - Weak regulatory enforcement of antibiotic sales - Limited availability of affordable, quality-assured antibiotics - Insufficient laboratory capacity for culture and susceptibility testing

Surveillance and monitoring: Establishment of systematic AMR surveillance networks at primary care facilities is essential to track resistance trends, evaluate interventions, and inform policy decisions. Integration with national and global AMR surveillance systems will enable comparative analysis and early detection of emerging resistance patterns.

References

1. Donkor ES, Horlortu PZ, Dayie NT, Obeng-Nkrumah N, Labi A-K. Community-acquired urinary tract infections among adults in Accra, Ghana. Infection and Drug Resistance. 2019:2059-67.
2. Murray CJ, Ikuta KS, Sharara F, Swetschinski L, Aguilar GR, Gray A, et al. Global burden of bacterial antimicrobial resistance in 2019: a systematic analysis. The Lancet. 2022;399(10325):629-55.
3. Phillip S, Mushi MF, Decano AG, Seni J, Mmbaga BT, Kumburu H, et al. Molecular characterizations of the coagulase-negative Staphylococci species causing urinary tract infection in Tanzania: a Laboratory-Based Cross-Sectional Study. Pathogens. 2023;12(2):180.
4. Maldonado-Barragán A, Mshana SE, Keenan K, Ke X, Gillespie SH, Stelling J, et al. Predominance of multidrug-resistant bacteria causing urinary tract infections among symptomatic patients in East Africa: a call for action. JAC-antimicrobial resistance. 2024;6(1):dlae019.
5. Msaki BP, Mshana SE, Hokororo A, Mazigo HD, Morona D. Prevalence and predictors of urinary tract infection and severe malaria among febrile children attending Makongoro health centre in Mwanza city, North-Western Tanzania. Archives of Public Health. 2012;70:1-8.
6. Gidabayda JG, Philemon R, Abdallah MS, Saajan AM, Temu T, Kunjumu I, et al. Prevalence, aetiology, and antimicrobial susceptibility patterns of urinary tract infection amongst children admitted at Kilimanjaro Christian Medical Centre, Moshi, Tanzania. The East African Health Research Journal. 2017;1(1):53.
7. Chaula T, Seni J, Ng'walida N, Kajura A, Mirambo MM, DeVinney R, et al. Urinary Tract Infections among HIV-Positive Pregnant Women in Mwanza City, Tanzania, Are High and Predicted by Low CD4+ Count. International journal of microbiology. 2017;2017(1):4042686.
8. Masoud SS, Majigo M, Silago V, Kunambi P, Nyawale H, Moremi N, et al. Utility of dipstick urinalysis in the diagnosis of urinary tract infections among outpatients in Mwanza and Dar es Salaam regions in Tanzania. Bulletin of the National Research Centre. 2024;48(1):6.
9. Silago V, Moremi N, Mtebe M, Komba E, Masoud S, Mgaya FX, et al. Multidrug-resistant uropathogens causing community-acquired urinary tract infections among patients attending health facilities in Mwanza and Dar es Salaam, Tanzania. Antibiotics. 2022;11(12):1718.
10. Kabugo D, Kizito S, Ashok DD, Kiwanuka AG, Nabimba R, Namunana S, et al. Factors associated with community-acquired urinary tract infections among adults attending the assessment centre, Mulago Hospital, Uganda. African health sciences. 2016;16(4):1131-42.
11. Mitu FS, Al Maruf MA, Mahanty A, Huda AN, Khan SA, Rahman MM. Prevalence of extended-spectrum beta-lactamase (ESBL) and AmpC beta-lactamase-producing bacteria in urinary tract infection patients in Bangladesh. Malaysian journal of microbiology. 2019;15(3):204-12.
12. Bedenić B, Meštrović T. Mechanisms of resistance in gram-negative urinary pathogens: From country-specific molecular insights to global clinical relevance. Diagnostics. 2021;11(5):800.
13. Mlugu EM, Mohamedi JA, Sangeda RZ, Mwambete KD. Prevalence of urinary tract infection and antimicrobial resistance patterns of uropathogens with biofilm-forming capacity among outpatients in Morogoro, Tanzania: a cross-sectional study. BMC infectious diseases. 2023;23(1):660.
14. Ngowi BN, Sunguya B, Herman A, Chacha A, Maro E, Rugarabamu LF, et al. Prevalence of multidrug-resistant UTI among people living with HIV in Northern Tanzania. Infection and drug resistance. 2021:1623-33.

15. Konje ET, Kizenga O, Charco NJ, Kibwana UO, Shango N, Tarimo F, et al. Five-year cross-sectional study to determine the burden of Candida spp. Infections of the urinary tract system among patients attending a tertiary hospital in Northwestern Tanzania. BMJ Open. 2023;13(12):e074833.

16. Ministry of Health CD, Gender, Elderly, and Children. Standard Treatment Guidelines and National Essential Medicines List for Tanzania Mainland. Dar es Salaam: Ministry of Health, Community Development, Gender, Elderly, and Children. 2021;6th Edition.

17. Kish L. Sampling organizations and groups of unequal sizes. American Sociological Review. 1965:564-72.

18. Chard T. Pregnancy tests: a review. Human reproduction. 1992;7(5):701-10.

19. Majigo MV, Mshana S, Komba E, Moremi N, Matee M. Protocol for surveillance of antimicrobial-resistant bacteria causing community-acquired urinary tract infections in low-income countries. Plos one. 2024;19(5):e0304388.

20. D.A. P, W. G, L.C. D, S.H. G, M.J. W, E.W. K. Koneman's Color Atlas and Textbook of Diagnostic Microbiology. Philadelphia, PA, USA: Wolters Kluwer Health; 2017.

21. Alghamdi A, Almajid M, Alalawi R, Alganame A, Alanazi S, Alghamdi G, et al. Evaluation of asymptomatic bacteruria management before and after antimicrobial stewardship program implementation: retrospective study. BMC Infectious Diseases. 2021;21:1-5.

22. Carbonnelle E, Mesquita C, Bille E, Day N, Dauphin B, Beretti J-L, et al. MALDI-TOF mass spectrometry tools for bacterial identification in a clinical microbiology laboratory. Clinical biochemistry. 2011;44(1):104-9.

23. CLSI. CLSI. *Performance standards for Antimicrobial Susceptibility Testing. 33rd ed.*CLSI Supplement M100. Wayne, PA: Clinical and Laboratory Standards Institute; 2023; 2023.

24. Ndaki PM, Mushi MF, Mwanga JR, Konje ET, Mugassa S, Manyiri MW, et al. Non-prescribed antibiotic dispensing practices for symptoms of urinary tract infection in community pharmacies and accredited drug dispensing outlets in Tanzania: a simulated client approach. BMC Primary Care. 2022;23(1):287.

25. Ndaki PM, Mwanga JR, Mushi MF, Konje ET, Mwita SM, Mshana SE. Drivers of inappropriate use of antibiotics among community members in low-and middle-income countries: a systematic review of qualitative studies. BMC Public Health. 2025;25(1):705.

26. Mendelson M, Matsoso MP. The World Health Organization's global action plan for antimicrobial resistance. SAMJ: South African Medical Journal. 2015;105(5):325-.

27. Organization WH. Antimicrobial stewardship programmes in health-care facilities in low-and middle-income countries: a WHO practical toolkit. 2019.

28. Organization WH. Antimicrobial resistance: global report on surveillance: World Health Organization; 2014.

29. Organization WH. Kenya national action plan on antimicrobial resistance: review of progress in the human health sector. 2022.

30. Charani E, Mendelson M, Pallett SJ, Ahmad R, Mpundu M, Mbamalu O, et al. An analysis of existing national action plans for antimicrobial resistance—gaps and opportunities in strategies optimising antibiotic use in human populations. The Lancet Global Health. 2023;11(3):e466-e74.

31. Organization WH. WHO AWaRe Classification of Antibiotics for Evaluation and Monitoring of Use. 2019. WHO; 2019.

32. Green DL, Keenan K, Fredricks KJ, Huque SI, Mushi MF, Kansiime C, et al. The role of multidimensional poverty in antibiotic misuse: a mixed-methods study of self-medication and non-adherence in Kenya, Tanzania, and Uganda. The Lancet Global Health. 2023;11(1):e59-e68.

33. Flores-Mireles AL, Walker JN, Caparon M, Hultgren SJ. Urinary tract infections: epidemiology, mechanisms of infection and treatment options. Nature reviews microbiology. 2015;13(5):269-84.

34. MoHT. Standard Treatment Guidelines and National Essential Medicines List for Tanzania Mainland. 6th ed. Dodoma, Tanzania: Ministry of Health, Tanzania; 2021. p. 491-3.

35. Silago V, Keenan K, Mushi MF, Kansiime C, Asiimwe B, Sunday B, et al. Patterns of antibiotic resistance in urinary tract infections before and during the COVID-19 pandemic in Uganda and Tanzania. JAC-Antimicrobial Resistance. 2025;7(2):dlaf038.

figure legend

Figure 1: The proportions of bacterial species causing community-acquired urinary tract infections at Buzuruga and Igoma Health Centers in Mwanza, Tanzania. *All (n=13): *Morganella morganii* (n=3), *Enterobacter cloacae* complex (n=2), *Citrobacter koseri* (n=1), *Klebsiella oxytoca* (n=1), *Pseudomonas fluorescens* (n=1), *Proteus mirabilis* (n=1), *Pseudoglutamicibacter cumminsii* (n=1), *Streptococcus agalactiae* (n=1), *Staphylococcus epidermidis* (n=1), and *Staphylococcus haemolyticus* (n=1). **Buzuruga HC (n=10):** *Morganella morganii* (n=3), *Enterobacter cloacae* complex (n=1), *Citrobacter koseri* (n=1), *Klebsiella oxytoca* (n=1), *Pseudomonas fluorescens* (n=1), *Pseudoglutamicibacter cumminsii* (n=1), *Staphylococcus epidermidis* (n=1), and *Staphylococcus haemolyticus* (n=1). **Igoma HC (n=3):** *Enterobacter cloacae* complex (n=1), *Proteus mirabilis* (n=1), and *Streptococcus agalactiae* (n=1).

Supplementary files

S1: Detailed antimicrobial susceptibility data

S2: Raw dataset

Figure Click here to access/download;Figure;Figure 1.tif

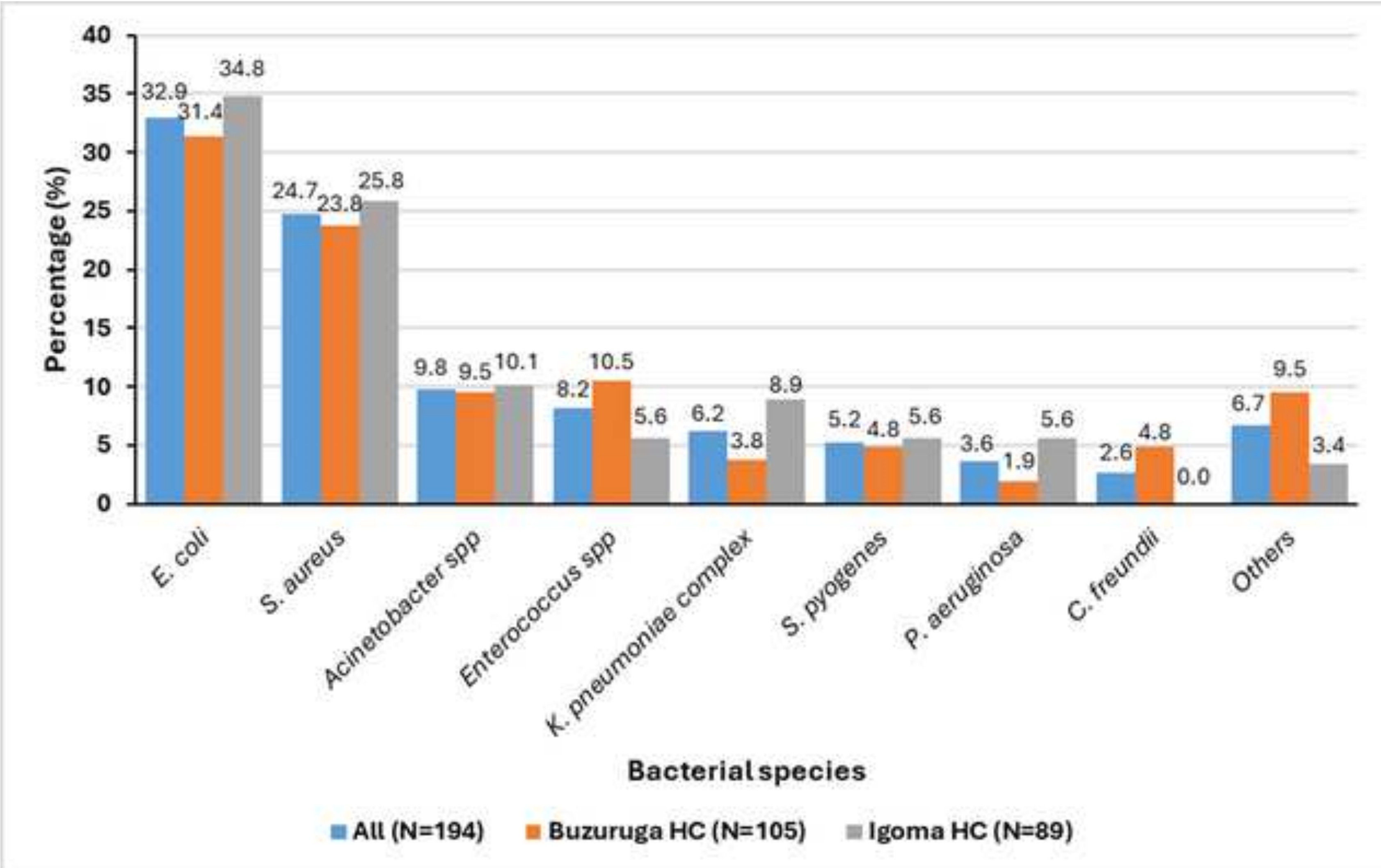

supplementary file 1

Click here to access/download
Supporting Information
Supplementary file 1.xlsx

supplementary file 2

Click here to access/download
Supporting Information
Supplementary file 2.xlsx

Multidrug resistance in bacteria causing community acquired urinary tract infections among adult outpatients attending primary healthcare facilities ~~lower-level health facilities~~ in Mwanza, Tanzania

Formatted: Font: Bold

Eunice G. Emmanuel[1,2], Ashura Khamis[1], Elikana Michael[1], Dorina Muhizi[1], James Thomas[1], Bernard Okamo[3], Farida I. Mkassy[4], Eveline T. Konje[4], Vitus Silago[5], Martha F. Mushi[5*], Stephen E. Mshana[5]

1. Weil Bugando School of Medicine, Catholic University of Health and Allied Sciences, P.O Box 1464, Mwanza, Tanzania
2. Pharmacy Department, Igoma Health Centre, Ilemela, Mwanza Tanzania
3. Department of Biochemistry and Molecular Biology Catholic University of Health and Allied Sciences, P.O Box 1464, Mwanza, Tanzania
4. Department of Epidemiology, Biostatistics and Behavioural science, School of Public Health, Catholic University of Health and Allied Sciences, P.O Box 1464, Mwanza, Tanzania
5. Department of Microbiology and immunology, Weil Bugando School of Medicine, Catholic University of Health and Allied Sciences, P.O Box 1464, Mwanza, Tanzania

Correspondence*: Martha F. Mushi, Email mushimartha@gmail.com

Abstract

Background: Urinary tract infections (UTIs) remain a common clinical condition requiring antibiotic prescription among adult outpatients in low- and middle-income countries (LMICs). Data regarding the prevalence, patterns of bacteria and their antibacterial susceptibility profile for community acquired UTI at primary healthcare facilities are limited, particularly in resource-constrained settings where empirical treatment contributes to increased antimicrobial resistance (AMR) and associated morbidity. ~~Data regarding the prevalence, patterns of bacteria and their antibacterial susceptibility profile for community acquired UTI are limited.~~

Methods: A primary healthcare facility ~~hospital~~ based cross-sectional study was conducted from May to August 2024 at Igoma and Buzuruga Health Centres in Mwanza. Symptomatic adults' patients, without recent hospitalization (within past 30 days) or urinary catheterization were enrolled. Clinical and demographic data were collected followed by standard mid-stream urine for culture and disc diffusion susceptibility testing. Descriptive data analysis was performed using STATA version 15.

Results: A total of 1,005 adult patients with the median age of 32 [IQR: 23–49] years were recruited, of whom 727 (72.3%) were female. The majority (64.3%) reported a previous history of UTI (within six months), and the median symptom duration before presentation was 8 [5–15] days. Microbiological confirmation of UTI was found in 221 patients (22.0%, 95% CI 19.5%-24.7%). The most frequently isolated uropathogens were *Escherichia coli* (32.9%) and *Staphylococcus aureus* (24.7%). Notably, only 35.7% of culture-confirmed UTI patients presented with classic urinary symptoms (dysuria, frequency, urgency), highlighting clinical diagnostic challenges in this setting. *E. coli* isolates demonstrated alarmingly high resistance rates: ciprofloxacin (62.3%), trimethoprim-sulfamethoxazole (60.7%), amoxicillin-clavulanic acid (57.4%), tetracycline (55.7%), and ampicillin (93.4%). Critically, 50% of *E. coli* isolates exhibited multidrug resistance (MDR), and 20.3% showed extended-spectrum beta-lactamase (ESBL) phenotypes. Among *S. aureus* isolates, 64.6% were resistant to cefoxitin, indicating methicillin-resistant *S. aureus*

(MRSA) strains.*Escherichia coli* isolates were ≥50% resistant to ciprofloxacin, amoxicillin-clavulanic acid, trimethoprim-sulfamethoxazole, tetracycline, and ampicillin. About 20.3% of *E. coli* isolates showed positive extended-spectrum beta-lactamase (ESBL) phenotypes whereas 64.6% of *S. aureus* were resistant to cefoxitin, hence methicillin-resistant *S. aureus* (MRSA) strains.

Conclusion and recommendation: This study revealed a significant burden of community-acquired urinary tract infections (CA-UTIs) among adult outpatients at primary healthcare facilities, with an overall prevalence of 22%. *E. coli* and *S. aureus* were the most frequently isolated pathogens, with alarmingly high levels of antimicrobial resistance, particularly multidrug resistance in *E. coli*. The high prevalence of atypical clinical presentations and the failure of commonly used first-line antibiotics (ciprofloxacin, trimethoprim-sulfamethoxazole, amoxicillin-clavulanic acid) underscore the urgent need for improved diagnostic capacity, strengthened antibiotic stewardship programs, and updated treatment guidelines tailored to local resistance patterns to guide effective management of community-acquired UTIs and control AMR development.This study revealed a significant burden of community-acquired urinary tract infections (CA-UTIs) among adult outpatients, with an overall prevalence of 22%. *Escherichia coli* and *Staphylococcus aureus* were the most frequently isolated pathogens, and alarmingly high levels of antimicrobial resistance, particularly multidrug resistance in *E. coli*. Improved diagnostic capacity and strengthened antibiotic stewardship are urgently needed to guide effective management of community-acquired UTIs to control the AMR development.

Key words: Community-acquired UTI; Antimicrobial resistance; *Escherichia coli*; Multidrug resistance; Uropathogens; Outpatients; Tanzania

Highlight:

- A total of 1,005 symptomatic adult patients at primary healthcare facilities were assessed for community-acquired urinary tract infections (CA-UTIs).

- CA-UTIs were identified in 22% of cases, with *Escherichia coli* and *Staphylococcus aureus* as the most common pathogens.
- *E. coli* demonstrated a notably high rate of multidrug resistance, with about 5082.8% of isolates resistant to multiple antibiotic classes.
- Over 70% of Gram-negative bacterial isolates were resistant to at least three classes of antibiotics, presenting major treatment difficulties.
- These results highlight the urgent need for ongoing surveillance and updated empirical treatment policies for UTIs in outpatient clinics, especially in resource-limited settings.

Background

Urinary tract infections (UTIs) are prevalent health concern among patients visiting outpatient clinics in low- and middle-income countries (LMICs). In these regions, UTIs significantly contribute to morbidity, elevated treatment expenses, and mortality, especially among those seeking care at primary health care ~~lower-tier health~~ facilities [1, 2]. Limited diagnostic resources coupled with insufficient microbiological evidence often compromise effective patient management. However, UTI remain one of ~~is~~ the most common reason for antibiotics ~~AB~~ prescription and the most frequent reason for hospital attendance among children and adults in Tanzania surpassed only by malaria [3, 4]. In Tanzania, the prevalence of UTI is reported to be different among populations ranging from 11-23% [5-7]. A previous study conducted in lower health facilities in ~~Tanzania~~ among patients of all age groups, reported the culture proven prevalence of 27.4% [8, 9].

Gram negative bacteria in the order Enterobacterales such as *E. coli* and *Klebsiella pneumoniae* are the most common uropathogens associated with both community and health care settings UTIs [1, 9]. These isolates have been found to produce extended spectrum beta-lactamase (ESBL).Among Gram positive bacteria, *Staphylococcus aureus* has been reported as the leading pathogen [10]. In LMICs studies have documented enzyme production like extended spectrum beta-lactamase (ESBL) as the main mechanism of resistance among Gram negative bacteria causing UTIs [11, 12]. Previous studies conducted in Tanzania reported the prevalence of multi drug resistant (MDR) bacteria causing UTIs to range from 22.4%-60.3% among Gram positive bacteria and 45.4%-52.2% among Gram negative bacteria [4, 9, 13, 14].

Several factors increase the risk of community-acquired urinary tract infections (CA-UTIs). Demographic and physiological factors are significant, with women being more vulnerable due to their shorter urethra and its proximity to the anus. Older adults are at greater risk due to weakened immune defences and underlying health conditions, while pregnancy increases susceptibility because of hormonal changes and pressure on the bladder[15]. Behavioural and lifestyle factors, such as poor hygiene, frequent sexual

activity, and the use of spermicides or diaphragms, can disrupt the natural bacterial balance in the urinary tract, further increasing the likelihood of infection.

Additionally, certain medical conditions and structural abnormalities heighten the risk. Individuals with diabetes mellitus are more prone to UTIs due to compromised immune function and elevated glucose levels that encourage bacterial growth. Conditions such as kidney stones, congenital abnormalities, or an enlarged prostate can obstruct urine flow, fostering an environment for bacterial proliferation. ~~People with history of recurrent UTIs are also at higher risk for future infections.~~ Medications and medical interventions are other contributing factors, as the use of antibiotics can disturb the balance of normal flora, and temporary catheterization, even at home, can increase the likelihood of bacterial colonization[4]. Furthermore, antimicrobial resistance patterns vary significantly by geographic location and change over time, making local surveillance data essential for guiding empirical treatment decisions. Previous studies in Tanzania have primarily focused on tertiary hospitals or specific populations such as pregnant women, children, or people living with HIV[4-7, 9, 13, 14] leaving a significant knowledge gap regarding CA-UTIs among general adult outpatients at primary healthcare facilities. Understanding the prevalence, causative organisms, and resistance patterns in this setting is essential for developing targeted interventions, updating treatment guidelines, and implementing effective antimicrobial stewardship programs.

This study aimed to determine the prevalence of CA-UTIs, identify the causative bacterial pathogens, and characterize their antimicrobial susceptibility profiles among adult outpatients attending primary healthcare facilities in Mwanza, Tanzania. By focusing on the primary care setting where most patients first present, this research provides critical insights into the current state of AMR in community settings and informs public health strategies to combat the rising threat of multidrug-resistant infections.

~~This study was conducted to document the prevalence, factors associated and profile of bacteria causing CA-UTIs. The antimicrobial susceptibility pattern of these uropathogens are well depicted.~~

Methodology

Study design and settings: This was a primary healthcare facilityhospital- based cross-sectional study conducted from 15th May to 30th August 2024, at Igoma and Buzuruga Health Centre in Mwanza, Tanzania. Buzuruga Health Centre serves up to 2,266 patients per month while Igoma Health Centre attend approximately 1,500 outpatients monthly.

Sample size and study population: The study population comprised adult outpatients (≥18 years) presenting with symptoms suggestive of UTI. According to established clinical guidelines[16], participants were when presenting with at least two of the following symptoms: dysuria, urinary frequency, urinary urgency, suprapubic pain, or lower abdominal pain. The study involved symptomatic adult patients presenting with signs and symptoms of UTIs. To be enrolled in the study, the patient was supposed to present with at least two of the following UTI symptoms; dysuria, frequency, urgency, nocturia, suprapubic pain, haematuria, and fever. The minimum sample size was 306 symptomatic adults obtained by Kish-Leslie formula [17] using a 24.7% prevalence of CA-UTIs -previously reported by Silago et *al*.[9]. To account for potential non-response and ensure adequate power for subgroup analyses, the sample was increased, resulting in a target enrollment of 1,005 participants. Pregnant women, patients with a history of admission within 30 days on the day of sample collection, and those with an indwelling urinary catheter at the time of study were excluded.

Data and sample collections: Demographic and clinical data were collected using epicollect5 Epicollect5, a mobile data-gathering platform developed by the CGPS Team of Oxford BDI and publicly available at https://five.epicollect.net. Information on prior antibiotic use was collected based on recent self-reported use to minimize recall bias and improve data reliability. About 3-5 ml of clean catch midstream urine was collected by the patients after receiving instructions from the research assistant, using a sterileclean, wide-mouthed, labelled container. The urine sample was transported to the microbiology research laboratory of CUHAS for processing within 2 hours of collection. For women, urine samples were tested

Formatted: Font: Not Bold

Formatted: Font: Not Bold

Formatted: Font: Not Bold

for HCG to exclude pregnancy[18]. Urine was divided to two portions for microscopy/dipstick analysis and culture. All laboratory data were documented on the laboratory logbook.

Urine Culture: Urine samples were inoculated onto agar media (Blood Agar, MacConkey Agar and CLED) and incubated at 37°c for 18-24 hours. Growth estimate of ≥1000 colonies from 10µl inoculating loop for both men and women was considered as significant bacteriuria and thus UTI [19]. In-house-prepared conventional biochemical identification tests including: catalase, coagulase, oxidase, indole, citrate, urease, and sugar fermentation tests ~~-~~were used for the preliminary identification of bacteria isolates to their possible species levels as previously described [20]. Gram-positive and Gram-negative bacteria were identified using morphological and biochemical tests as previously described [21]. Unidentified isolates were further analysed using Matrix-Assisted Laser Desorption Ionization–Time of Flight (MALDI-TOF) mass spectrometry at the National Public Health Laboratory (NPHL)[22]. Antimicrobial susceptibility testing was performed as per CLSI guidelines [23].

Quality Control: Quality assurance measures encompassed monitoring reagent storage, equipment functionality, media sterility, and preparation of standardized bacterial suspensions. All stains and reagents were systematically labelled, dated, and stored under appropriate conditions. Refrigerator and incubator temperatures were recorded and tracked daily to ensure compliance with operational standards. Culture media were prepared in strict accordance with manufacturer instructions and evaluated for both performance and sterility. To maintain consistent inoculum density for susceptibility testing, a 0.5 McFarland standard was utilized; standard reference strains like *S. aureus* (ATCC 25923), *MRSA* (ATCC 29213), *E. coli* (ATCC 25922), ESBL-producing *Klebsiella pneumoniae* (ATCC 700603), and *Pseudomonas aeruginosa* (ATCC 27853) were employed as controls. Data cleaning and coding were conducted using STATA version 15, with records containing missing values excluded from analysis.

Data Analysis: Data were analysed using STATA software version 15 in line with the study's objectives. Descriptive statistics were used to summarize data, with continuous variables presented as medians with interquartile ranges and categorical variables as frequencies and proportions ~~Continuous (age and~~

duration of symptoms) and categorical variables (gender, education, marital status, clinical presentation, isolates and antimicrobial susceptibility testing profile) were summarized using medians and proportions, respectively..

Ethical Considerations: Ethical clearance was obtained from the Joint CUHAS/BMC Research Ethics and Review Committee (CREC) with certificate number CREC No 763/2024. All participants were requested to consent by signing written informed consent form. Privacy and confidentiality were maintained throughout the study. The urine culture results were timely shared to the manage clinicians to guide the management.

Results

The study recruited a total of 1005 adult, patients with a clinical diagnosis of UTI. In general, majority of recruited patients were non-pregnant women 727(72.3%), about two third were married 610 (60.7%) and their median age was 32[23-49] years, as detailed in table1.

Table 1: Social demographic characteristics of the studied patients

**Variable**		**Buzuruga HC (566) n(%)**	**Igoma HC (439) n(%)**	**Total (1005)**
Gender	Male	138(24.4)	140(31.9)	278(27.7)
	Female	428(75.6)	299(68.1)	727(72.3)
Age	Median [IQR]	32[23-50]	30[22-49]	32[23-49]
Education	No formal education	37(6.5)	65(14.8)	102(10.2)
	Primary	243(42.9)	216(49.2)	459(45.7)
	Secondary	220(38.9)	123(28.0)	343(34.1)
	University	66(11.7)	35(7.9)	101(10.1)
Marital status	Married	367(64.8)	243(55.4)	610(60.7)
	Single	199(35.2)	196(44.6)	395(39.3)

Regarding clinical history, 646 participants (64.3%) reported a previous episode of UTI within the past six months, and 129 (12.8%) had used antibiotics within the 14 days preceding enrollment. The median duration of symptoms before seeking care was 8 days (IQR: 5-15 days). Notably, only 359 participants (35.7%) presented with classic urinary tract symptoms (dysuria, frequency, and urgency), while the majority presented with non-specific symptoms such as lower abdominal pain, fever, or lower back pain, highlighting the clinical diagnostic challenges in this setting.

~~In general, about two thirds of the patients had previous history of UTI diagnosis (within past six months) 646(64.3%), and the median duration of the current symptoms before reporting to the health facility was~~

8[5-15] days. Patients recruited from Igoma health centre reported late to the health facility than those from Buzuruga health centre (14[7-28] vs. 7[3-14] days), P=0.001.

Though more than half, 588(58.5%), reported to have fever (>=38.5 °C) only few reported to have classical symptoms of UTI like dysuria 254(25.3%), urgency 70(6.9%) cloudy urine 89(8.9%) and smelling urine 54(5.4%). Prior antibiotic use (within a week) for treatment of the current symptoms was reported by 12.8% (129) patients, aas indicated in table 12.

Among the recruited patients with other comorbidities 122, high blood pressure was the commonest, 38 (31.1%). A total of seven patients had more than one chronic condition (comorbidity) such as diabetic mellitus and hypertension (six patients), and one patient had both having hypertension and peptic ulcer diseases making total count of 122.

Formatted: Highlight

Table 2: Clinical characteristics of the recruited patients

Variable		Buzuruga HC (566) n(%)	Igoma HC(439) n(%)	Total (1005)
Past History of UTI	Yes	382(67.5)	264(60.1)	646(64.3)
	No	184(32.5)	175(39.9)	359(35.7)
Dysuria	Yes	137(24.2)	117(26.6)	254(25.3)
	No	429(75.8)	322(73.4)	751(74.7)
Urgency	Yes	54(9.5)	16(3.6)	70(6.9)
	No	512(90.5)	423(96.4)	935(93.0)
Frequency	Yes	66(11.7)	40(9.1)	106(10.6)
	No	500(88.3)	399(90.9)	899(89.4)
Nocturia	Yes	20(3.5)	22(5.0)	42(4.2)
	No	546(96.5)	417(94.9)	963(95.8)
Cloudy urine	Yes	58(10.3)	31(7.1)	89(8.9)
	No	508(89.7)	408(92.9)	916(91.1)
Smelling urine	Yes	40(7.1)	14(3.2)	54(5.4)
	No	526(92.9)	425(96.8)	951(94.6)
Lower abdominal pain	Yes	275(48.6)	237(53.9)	512(50.9)
	No	291(51.4)	202(46.0)	493(49.1)
Lower back pain	Yes	161(28.5)	212(48.3)	373(37.1)
	No	405(71.5)	227(51.7)	632(62.9)
Fever	Yes	377(66.6)	211(48.1)	588(58.5)
	No	189(33.4)	228(51.9)	417(41.5)
Duration of symptoms (days)		7[3-14]	14[7-28]	8[5-15]
Presence of comorbidity	Yes	96(16.9)	26(5.9)	122(12.1)
	No	470(83.0)	413(94.1)	883(87.9)
Antibiotic use for current infection (within a week)	Yes	89(15.7)	40(9.1)	129(12.8)
	No	477(84.3)	399(90.9)	876(87.2)
Co-morbidity	High blood Pressure	26(27.1%)	12(46.2%)	38(31.1%)
	Diabetic Mellitus	19(19.8%)	6(23.1%)	25(20.5%)
	Chronic Back Pain	19(19.8%)	4(15.4%)	23(18.9%)
	Peptic Ulcer Diseases	23(23.9%)	0(0.0%)	23(18.9%)
	Others	9(9.4%)	4(15.4%)	13(10.7%)

Others included Benign prostatic hyperplasia 2; living with HIV 6; chronic leg pain 1; ovarian cyst 4

Prevalence of community acquired UTI

Of the 1005 recruited patients with clinical presentation of urinary tract infections, 221(22%), 95% CI 19.4%-24.6% were microbiologically confirmed. Igoma health centre had 22.8%, (n=100/439), 95% CI 18.9%-27% patients with microbiologically confirmed UTI while Buzuruga health centre had 21.4%, (n=121/566), 95% CI 18%-25% patients.

Proportions of bacterial species causing CA-UTIs

Of the 221 microbiologically confirmed cases, 194 were due to bacterial isolates, while 27 showed significant growth of *Candida* spp. ~~A total of 194 bacterial isolates were identified from significant positive urine samples, with~~ The ~~the~~ majority of bacteria isolated were ~~being~~ Gram-negative ~~bacteria~~ (59.8%; n = 116). Overall, the most frequently detected bacterial species were *E. coli* (32.9%; n = 64) and *S. aureus* (24.7%; n = 48). A similar pattern of predominant bacteria pathogens was observed at both Buzuruga Health Centre and Igoma Health Centre, however, the third and fourth most common isolates differed slightly between the two sites. At Buzuruga HC, *Enterococcus* spp. (10.5%; n = 11) and *Acinetobacter* spp. (9.5%; n = 10) were the commonest third and fourth pathogen, respectively while at Igoma *Acinetobacter* spp. (10.1%; n = 9) and *K. pneumoniae* complex (8.9%; n = 8) were common. Notably, all *C. freundii* strains were exclusively isolated at Buzuruga HC (Table 2~~3, /~~/Figure 1~~2~~).

Formatted: Font: Italic

Table 3: The proportions of bacterial species causing community-acquired urinary tract infections at Buzuruga and Igoma Health Centers in Mwanza, Tanzania

Isolate	All (n=194)	Buzuruga HC (N=105)	Igoma HC (N=89)
	n(%)	n(%)	n(%)
E. coli	64 (32.9)	33 (31.4)	31 (34.8)
S. aureus	48 (24.7)	25 (23.8)	23 (25.8)
Acinetobacter spp.	19 (9.8)	10 (9.5)	9 (10.1)
Enterococcus spp.	16 (8.2)	11 (10.5)	5 (5.6)
K. pneumoniae complex	12 (6.2)	4 (3.8)	8 (8.9)
S. pyogenes	10 (5.2)	5 (4.8)	5(5.6)
P. aeruginosa	7 (3.6)	2 (1.9)	5 (5.6)
C. freundii	5 (2.6)	5 (4.8)	0 (0.0)
Others*	13 (6.7)	10 (9.5)	3 (3.4)

***Others:**

All (n=13): *Morganella morganii* (n=3), *Enterobacter cloacae* complex (n=2), *Citrobacter koseri* (n=1), *Klebsiella oxytoca* (n=1), *Pseudomonas fluorescens* (n=1), *Proteus mirabilis* (n=1), *Pseudoglutamicibacter cumminsii* (n=1), *Streptococcus agalactiae* (n=1), *Staphylococcus epidermidis* (n=1), and *Staphylococcus haemolyticus* (n=1).

Buzuruga HC (n=10): *Morganella morganii* (n=3), *Enterobacter cloacae* complex (n=1), *Citrobacter koseri* (n=1), *Klebsiella oxytoca* (n=1), *Pseudomonas fluorescens* (n=1), *Pseudoglutamicibacter cumminsii* (n=1), *Staphylococcus epidermidis* (n=1), and *Staphylococcus haemolyticus* (n=1).

Igoma HC (n=3): *Enterobacter cloacae* complex (n=1), *Proteus mirabilis* (n=1), and *Streptococcus agalactiae* (n=1).

Formatted: Normal, Line spacing: single, Don't keep with next

Formatted: Space After: 8 pt, Line spacing: Multiple 1.08 li

Formatted: Left, Space After: 8 pt, Line spacing: Multiple 1.08 li

Formatted: Space After: 8 pt, Line spacing: Multiple 1.08 li

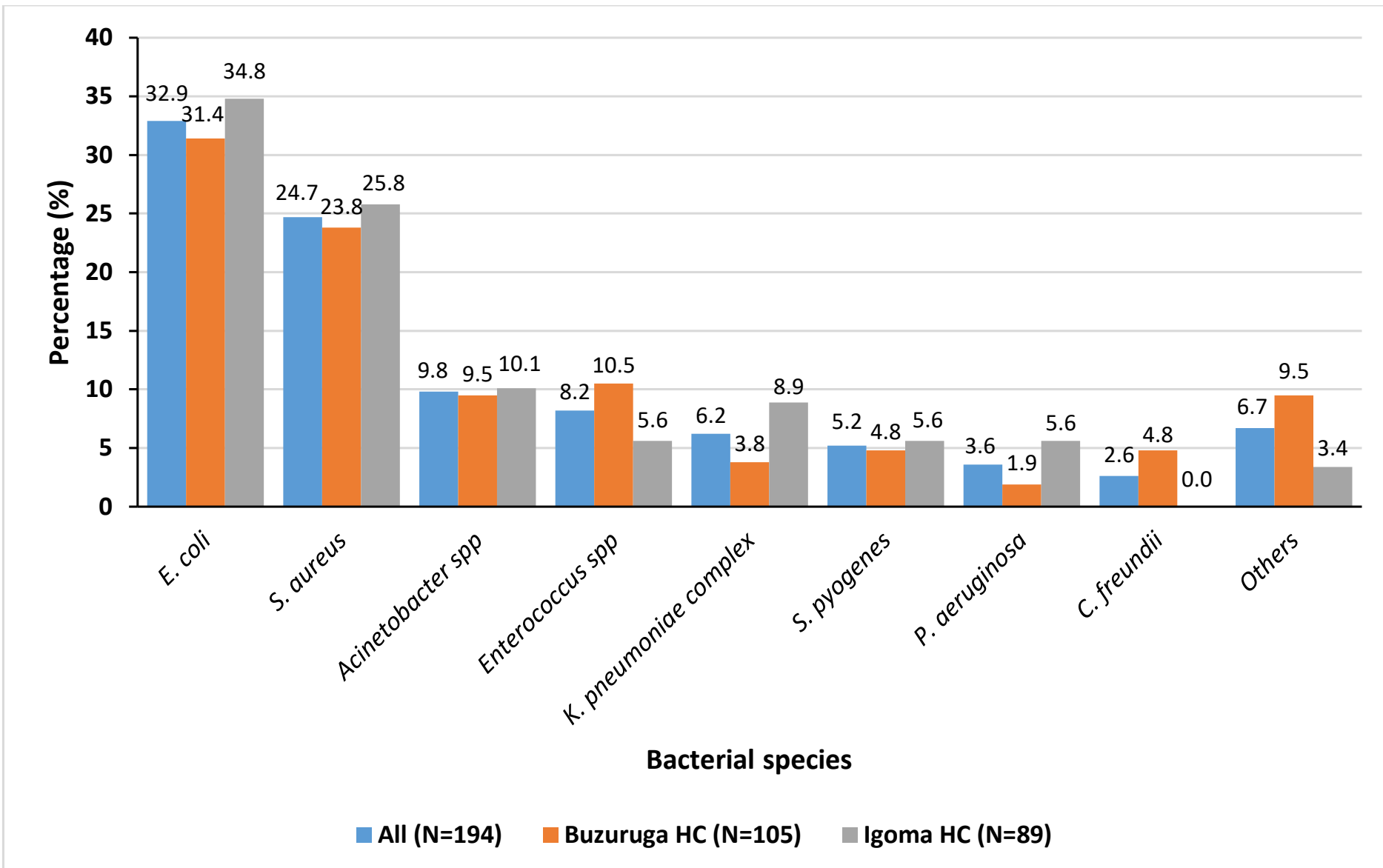

~~***Figure 2: The proportions of bacterial species causing community-acquired urinary tract infections at Buzuruga and Igoma Health Centers in Mwanza, Tanzania.*** ***All (n=13):** *Morganella morganii* (n=3), *Enterobacter cloacae* complex (n=2), *Citrobacter koseri* (n=1), *Klebsiella oxytoca* (n=1), *Pseudomonas fluorescens* (n=1), *Proteus mirabilis* (n=1), *Pseudoglutamicibacter cumminsii* (n=1), *Streptococcus agalactiae* (n=1), *Staphylococcus epidermidis* (n=1), and *Staphylococcus haemolyticus* (n=1). **Buzuruga HC (n=10):** *Morganella morganii* (n=3), *Enterobacter cloacae* complex (n=1), *Citrobacter koseri* (n=1), *Klebsiella oxytoca* (n=1), *Pseudomonas fluorescens* (n=1), *Pseudoglutamicibacter cumminsii* (n=1), *Staphylococcus epidermidis* (n=1), and *Staphylococcus haemolyticus* (n=1). **Igoma HC (n=3):** *Enterobacter cloacae* complex (n=1), *Proteus mirabilis* (n=1), and *Streptococcus agalactiae* (n=1).~~

Percentage resistance of bacterial pathogens causing CA-UTIs

Escherichia coli demonstrated the highest levels of antimicrobial resistance (≥50%) to multiple antibiotics, including ciprofloxacin, amoxicillin-clavulanic acid, trimethoprim-sulfamethoxazole, tetracycline, and ampicillin. About 20.3% of *E. coli* isolates showed positive phenotypes of extended-spectrum beta-lactamase (ESBL) production. Other members of the Enterobacterales group showed similarly high resistance rates to amoxicillin-clavulanic acid and ampicillin, with 12.0% detected with ESBL phenotypes. The non-fermentative Gram-negative bacteria,

Formatted: Space After: 8 pt, Line spacing: Multiple 1.08 li, Don't keep with next

Formatted: Left, Space After: 8 pt, Line spacing: Multiple 1.08 li

Formatted: Normal, Line spacing: single

specifically *Acinetobacter* spp. and *Pseudomonas* spp., exhibited the highest resistance to amoxicillin-clavulanic acid, trimethoprim-sulfamethoxazole, ceftriaxone, and ampicillin (Table 34).

Table 4: Percentage resistance of Gram-negative bacteria causing community-acquired urinary tract infection at Buzuruga and Igoma Health Centers in Mwanza, Tanzania

Antibiotic agents tested	*E. coli* (N=64)		Other Enterobacterales (N=25)		Non-fermentative Gram-negative bacteria (N=27)	
	% Resistance	% Sensitive	% Resistance	% Sensitive	% Resistance	% Sensitive
AMP	58 (90.6)	6 (9.4)	25 (100)	0 (0.0)	5 (100)	0 (0.0)
TET	47 (73.4)	17 (26.6)	10 (40.0)	15 (60.0)	11 (47.8)	12 (52.2)
SXT	46 (73.0)	17 (27.0)	10 (40.0)	15 (60.0)	16 (59.3)<br>11 (40.7)	
CIP	34 (53.1)	30 (46.9)	8 (32.0)	17 (68.0)	9 (33.3)	18 (66.7)
NIT	26 (40.6)	38 (59.4)	14 (56.0)	11 (44.0)	25 (92.6)	2 (7.4)
AMC	41 (64.1)	23 (35.9)	13 (52.0)	12 (48.0)	2 (50.0)<br>2 (50.0)	
CRO	29 (45.3)	35 (54.7)	11 (44.0)	14 (56.00)	21 (80.8)<br>5 (19.2)	
GEN	29 (45.3)	35 (54.7)	6 (25.0)	18 (75.0)	4 (15.4)	22 (86.4)
MEM	5 (7.9)	58 (92.1)	4 (16.0)	21 (84.0)	0 (0.0)	26 (100)
ESBL strains	13 (20.3)		3 (12.0)		NA	

Key: AMP=ampicillin, TET=tetracycline, SXT=trimethoprim-sulfamethoxazole, CIP=ciprofloxacin, NIT=nitrofurantoin, AMC=amoxicillin-clavulanic acid, CRO=ceftriaxone, GEN=gentamicin, NA=not applicable, and MEM=meropenem.

On the other hand, *S. aureus* demonstrated markedly high resistance rates (≥ 50%) to ciprofloxacin, tetracycline, trimethoprim-sulfamethoxazole, ampicillin, and erythromycin. Moreover, 64.6% of *S. aureus* strains showed resistance towards cefoxitin, hence methicillin-resistant *S. aureus* (MRSA). Similarly, other

Formatted Table

Gram-negative bacteria exhibited peak resistance (≥ 50%) to ampicillin, tetracycline, trimethoprim-sulfamethoxazole, ciprofloxacin, and erythromycin (Table 3).

~~**Table 5: Percentage resistance of Gram-positive bacteria causing community-acquired urinary tract infection at Buzuruga and Igoma Health Centers in Mwanza, Tanzania**~~

~~Key: ER=erythromycin; CIP=ciprofloxacin; SXT=trimethoprim-sulfamethoxazole; TET=tetracycline; PEN=penicillin; CD=clindamycin; GEN=gentamicin; NIT-nitrofurantoin; and FOX=cefoxitin.~~

Proportions of multidrug resistance and resistance patterns of Gram-negative bacteria causing CA-UTIs

Overall, the majority of Gram-negative bacteria (73.3%; 85/116) exhibited resistance to at least three classes of antimicrobials, with the number of classes ranging from three to six. By healthcare facility, the difference in proportions of MDR in Gram-negative bacteria isolated from patients attending at Buzuruga HC (70.5%; 43/61) and Igoma HC (76.4%; 42/55) was not statistically significant (p=0.475). *E. coli* showed the highest proportion of multidrug resistance (MDR), with 82.8% (53/64) of isolates classified as MDR, including 15.1% (n = 8) that were resistant to six antimicrobial classes. The common resistance patterns in *E. coli* were AMP-TET-SXT-CIP-NIT-AMC-CRO-CAZ-FEP-GEN-AK-TZP (n=3), AMP-TET-SXT-CIP-NIT-AMC-CRO-CAZ-FEP-GEN-TZP (n=2), and AMP-TET-SXT-CIP-AMC-CRO-CAZ-FEP-AK-TZP (n=2). Among other Enterobacterales, 44.0% (11/25) were MDR, with 27.3% (n = 3) also resistant to six classes. Similarly, 77.8% (22/27) of non-fermentative Gram-negative bacteria displayed MDR, with 9.1% (n = 2) resistant to six antimicrobial classes. The common resistance patterns in non-fermentative Gram-negative bacteria were SXT-NIT-CRO-CAZ-FEP (n=2) and TET-SXT-NIT-CRO-FEP-TZP (n=2) (Table 34). Detailed antimicrobial resistance patterns are provided in Supplementary File 1.

~~**Table 6: Proportions of multidrug resistance in Gram-negative bacteria causing community-acquired urinary tract infection at Buzuruga and Igoma Health Centers in Mwanza, Tanzania**~~

Among GPB, a substantial proportion of *S. aureus* isolates (83.3%; 40/48) were classified as MDR. Notably, 15.0% (6/40) of these MDR *S. aureus* strains exhibited resistance to as many as seven antibiotics. MDR phenotypes were significantly more prevalent among *S. aureus* isolates from patients at Igoma Health Centre compared to those from Buzuruga HC (95.6% vs. 72.0%; p = 0.019). The most frequently observed resistance patterns in *S. aureus* included CD-ER-AMP-SXT-TET-CIP-FOX (n=3), ER-AMP-SXT-TET-CIP-FOX (n=3), and ER-AMP-SXT-TET-NIT-CIP-FOX-GEN (n=3). For other GPB, all isolates (100%; 29/29) were MDR. The most common resistance profiles among these included CD-ER-AMP-SXT-TET-CIP (n=2), CD-ER-NIT-CIP (n=2), and ER-AMP-SXT-TET-NIT-CIP (n=2). Moreover, 24.1% (7/29) of these MDR GPB strains demonstrated resistance to six antimicrobial agents (Table 4~~5~~). Detailed antimicrobial resistance patterns are shown on Supplementary file 2.

~~**Table 7: Proportions of multidrug resistance in Gram-positive bacteria causing community-acquired urinary tract infection at Buzuruga and Igoma Health Centers in Mwanza, Tanzania**~~

Discussion

This study provides important insights into the bacterial causes and antimicrobial resistance patterns of community-acquired urinary tract infections among patients attending primary healthcare facilities in Mwanza, Tanzania. The findings demonstrate that *Escherichia coli* remains the predominant uropathogen, with a substantial proportion of isolates exhibiting multidrug resistance. Notably, high resistance was observed to several commonly used antibiotics, raising concerns about the effectiveness of current empirical treatment practices in primary healthcare settings. Although only a small proportion of participants reported prior antibiotic use, the observed resistance patterns suggest ongoing antimicrobial

pressure within the community. Together, these findings highlight the growing public health challenge of antimicrobial resistance in community-acquired infections and underscore the need for strengthened surveillance, improved diagnostic capacity, and evidence-based antimicrobial stewardship to guide appropriate treatment.

The high prevalence of multidrug-resistant uropathogens observed in this study likely reflects multiple drivers operating at the community and healthcare levels. In many low- and middle-income countries, including Tanzania, antibiotics are frequently used empirically for urinary tract infections due to limited access to microbiological diagnostic services at primary healthcare facilities [24, 25]. In addition, inappropriate antibiotic use, including self-medication, incomplete treatment courses, and over-the-counter access to antimicrobials, has been reported as a major contributor to the emergence and spread of antimicrobial resistance in community-acquired infections[26, 27]. These factors create selective pressure that favors the survival and transmission of resistant bacterial strains. Addressing these drivers requires strengthening antimicrobial stewardship, improving access to diagnostic services to support targeted therapy, and enhancing surveillance of antimicrobial resistance to inform local treatment guidelines and public health interventions[28].

The antimicrobial resistance patterns observed in this study have important implications for empirical treatment of community-acquired urinary tract infections (CAUTIs) in primary healthcare settings. In our cohort, *Escherichia coli* was the predominant uropathogen, and a large proportion of isolates exhibited multidrug resistance, with high resistance observed to commonly prescribed antibiotics including amoxicillin-clavulanic acid and ciprofloxacin. These findings suggest that some antibiotics frequently used for empirical management of urinary tract infections may have reduced effectiveness in this setting. Similar resistance patterns have been reported in Tanzania –and other parts of East Africa, where increasing resistance among uropathogens has been associated with widespread empirical antibiotic use and limited diagnostic capacity[9, 24, 25]. Clinicians managing suspected urinary tract infections at primary healthcare facilities should therefore consider local antimicrobial susceptibility patterns when

selecting empirical therapy. Strengthening routine surveillance of antimicrobial resistance and integrating these data into national and facility-level treatment guidelines are essential to ensure that empirical treatment remains effective in the context of rising antimicrobial resistance [29-31]. ~~Urinary tract infection is currently a public health concern especially when acquired from community settings due to its high prevalence, increasingly drug-resistant, costly to treat, and associations with serious complications if not managed effectively. The disease is reported to be among the leading complain among patients attending outpatient's clinic[27]. It has gained more attention recently due to antimicrobial resistance associated with pathogens causing UTI that limit treatment option especially in lower- and middle-income settings. This study was conducted among community members attending the lower health facility with clinical signs and symptoms of UTI.~~

The prevalence of community-acquired, microbiologically confirmed UTI in the present study was 22%, which falls within the previously reported national range of 11–23% from diverse populations in Tanzania [5-7]. However, this prevalence is slightly lower than the 27% reported by Silago et al. in the same setting three years earlier [9]. This discrepancy may be explained by differences in study populations; the earlier study included pregnant women a group with a physiologically higher susceptibility to UTI while the current study excluded them.

From a public health perspective, a 22% prevalence indicates that UTIs remain a substantial community health burden, contributing to frequent outpatient visits and antimicrobial prescriptions. This underscores the need for strengthened community-level infection prevention strategies, including improved personal hygiene practices, safe water access, and health education aimed at early care seeking and avoidance of self-medication[32]. The high proportion of atypical presentations highlights the need for improved diagnostic capacity at primary care facilities. Point-of-care urine culture systems, dipstick testing, and clinical decision support tools could improve diagnostic accuracy and reduce inappropriate antibiotic use. Importantly, most participants reported prior antibiotic use, a finding with significant implications. Such

widespread community antibiotic exposure, often unregulated, can suppress bacterial growth and potentially lead to underestimation of true UTI prevalence while simultaneously exerting selection pressure that drives antimicrobial resistance (AMR).

In the current study, just like other studies, Gram-negative bacteria were predominant pathogens detected causing ~~community acquired~~community-acquired UTI. The predominance of Gram-negative bacteria is partly contributed to by the fact that these bacteria are normal microbiota of the gastrointestinal tract, and the close proximity of the urinary and gastrointestinal tract ~~make~~ makes it easy for the organism to ascend and cause a urinary tract infection.

The predominance of Gram-negative bacteria, especially *E. coli*, is consistent with global and regional trends in community-acquired urinary tract infections (CA-UTIs), reflecting its well-established role as the primary uropathogen [9]. The notable presence of *S. aureus*, a Gram-positive organism, suggests potential shifts or local variations in CA-UTI causes, warranting further exploration. *Staphylococcus aureus* is an uncommon cause of uncomplicated urinary tract infections and is more frequently associated with complicated infections or hematogenous spread. In this study, participants were enrolled based on symptoms suggestive of urinary tract infection at primary healthcare level, and detailed evaluation for systemic involvement or underlying comorbidities was not performed. However, identification of *S. aureus* was conducted using standard microbiological methods, making misidentification unlikely. The clinical significance of *S. aureus* isolated from urine in this context should therefore not underestimated.

Differences in less common isolates between the two health centres may be linked to local antimicrobial use, patient demographics, or environmental factors influencing bacterial populations. The exclusive detection of *C. freundii* at one site highlights potential micro-epidemiological differences, which could inform more targeted infection control and surveillance efforts. Although *Streptococcus pyogenes* is not a typical uropathogen, occasional cases have been reported, particularly in association with perineal colonization or ascending infection from the genital tract[33]. In this study, the organism was rarely isolated and its clinical significance should therefore be interpreted cautiously.

The high resistance observed, particularly among *E. coli* and non-fermentative Gram-negative bacteria, emphasizes the increasing challenge of treating CA-UTIs with commonly prescribed antibiotics [34]. These findings underscore the urgent need for ongoing surveillance, rational antimicrobial use, and updated treatment guidelines tailored to local resistance patterns to manage infections effectively and limit the spread of multidrug-resistant pathogens. The widespread multidrug resistance (MDR) observed in Gram-negative bacteria, with *E. coli* showing the highest levels, is particularly concerning, given its dominance as a uropathogens. Resistance to multiple antibiotic classes, including critical agents like piperacillin-tazobactam and amikacin, limits treatment options and reinforces the critical need for antimicrobial stewardship to address this growing threat.

The high MDR prevalence among *S. aureus* ~~(83.3%)~~ and other GPB ~~(100%)~~ in this study is consistent with findings by Silago et al. in Mwanza and Dar es Salaam, where similar resistance patterns to ciprofloxacin, tetracycline, and trimethoprim-sulfamethoxazole were observed [9]. Notably, the resistance profiles align with post-COVID-19 trends reported by the same group, showing persistent and widespread MDR uropathogens in Tanzania [35]. The significantly higher MDR burden at Igoma HC may reflect differences in local antimicrobial practices. These findings underscore the ongoing threat of resistant GPB pathogens and the urgent need for context-specific antimicrobial stewardship in outpatient settings. The resistance patterns observed in this study may therefore reflect ongoing community misuse or overuse of antibiotics previously reported [24, 25], reinforcing the urgency of antimicrobial stewardship interventions, regulation of non-prescription antibiotic sales, and public education on the risks of inappropriate antibiotic use.

Study limitations

Several limitations should be considered when interpreting these findings. The study was conducted over a relatively short period (4 months) during the dry season, and seasonal variations in UTI prevalence and etiology may exist. The study was limited to two primary healthcare facilities in an

urban/peri-urban setting, and findings may not be generalizable to rural areas or other regions of Tanzania. The broad case definition for UTI symptoms may have included some patients without true UTI, though this reflects real-world primary care practice where laboratory confirmation is often unavailable. Finally, information on antibiotic use was limited to the 14 days preceding enrollment, while longer-term antibiotic exposure (e.g., 90 days) may be more relevant for AMR risk assessment.

Conclusion, public health implication and Recommendation

Conclusion: This study revealed a significant burden of community-acquired urinary tract infections (CA-UTIs) among adult outpatients in Mwanza, Tanzania, with an overall prevalence of 22%. *Escherichia coli* and *Staphylococcus aureus* were the most frequently isolated pathogens, and alarmingly high levels of antimicrobial resistance particularly multidrug resistance in *E. coli* were observed. The late presentation of patients, limited recognition of classical UTI symptoms, and high recurrence rates further complicate clinical management.

Public health implications and recommendations

Urgent need for updated treatment guidelines: Current empirical treatment recommendations for CA-UTI in Tanzania are no longer supported by local susceptibility data. Treatment guidelines should be updated to reflect local resistance patterns, with consideration of alternative first-line agents such as nitrofurantoin for uncomplicated cystitis (where *E. coli* susceptibility remains 83.6%) or gentamicin for more severe infections, while reserving carbapenems for complicated cases or confirmed ESBL infections.

Strengthening antimicrobial stewardship: Comprehensive antimicrobial stewardship programs should be implemented at primary healthcare facilities, including: - Improved access to rapid diagnostic tests to guide targeted therapy, training of healthcare workers on appropriate antibiotic prescribing, public education campaigns on responsible antibiotic use, regulation of over-the-counter antibiotic sales and monitoring and surveillance of antibiotic consumption and resistance patterns

Formatted: Font: Not Bold

Formatted: Font: Not Bold

Formatted: Font: Not Bold

Formatted: Font: Not Bold

Formatted: Font: Not Bold

Formatted: Font: Not Bold

Enhanced diagnostic capacity: The high proportion of atypical presentations highlights the need for improved diagnostic capacity at primary care facilities. Point-of-care urine culture systems, dipstick testing, and clinical decision support tools could improve diagnostic accuracy and reduce inappropriate antibiotic use.

Addressing drivers of AMR: Interventions must address the underlying drivers of AMR in Tanzania, including: - Poverty and limited healthcare access leading to self-medication - Inadequate water, sanitation, and hygiene (WASH) infrastructure - Weak regulatory enforcement of antibiotic sales - Limited availability of affordable, quality-assured antibiotics - Insufficient laboratory capacity for culture and susceptibility testing

Surveillance and monitoring: Establishment of systematic AMR surveillance networks at primary care facilities is essential to track resistance trends, evaluate interventions, and inform policy decisions. Integration with national and global AMR surveillance systems will enable comparative analysis and early detection of emerging resistance patterns.

Recommendations: Routine microbiological surveillance should be established in outpatient settings to monitor the evolving antimicrobial resistance patterns and inform empirical treatment guidelines. Additionally, public health education campaigns are needed to raise awareness about early UTI symptoms, discourage self-medication, and encourage prompt medical consultation. Enhancing diagnostic capacity at primary healthcare facilities, including the availability of urine culture and sensitivity testing, will improve timely diagnosis and targeted treatment. Finally, national health authorities should consider updating and disseminating evidence-based treatment protocols for CA-UTIs that reflect local resistance trends and support implementation across all levels of care.

References

1. Donkor ES, Horlortu PZ, Dayie NT, Obeng-Nkrumah N, Labi A-K. Community acquired urinary tract infections among adults in Accra, Ghana. Infection and Drug Resistance. 2019:2059-67.

2. Murray CJ, Ikuta KS, Sharara F, Swetschinski L, Aguilar GR, Gray A, et al. Global burden of bacterial antimicrobial resistance in 2019: a systematic analysis. The lancet. 2022;399(10325):629-55.

3. Phillip S, Mushi MF, Decano AG, Seni J, Mmbaga BT, Kumburu H, et al. Molecular characterizations of the coagulase-negative Staphylococci species causing urinary tract infection in Tanzania: a Laboratory-Based Cross-Sectional Study. Pathogens. 2023;12(2):180.

4. Maldonado-Barragán A, Mshana SE, Keenan K, Ke X, Gillespie SH, Stelling J, et al. Predominance of multidrug-resistant bacteria causing urinary tract infections among symptomatic patients in East Africa: a call for action. JAC-antimicrobial resistance. 2024;6(1):dlae019.

5. Msaki BP, Mshana SE, Hokororo A, Mazigo HD, Morona D. Prevalence and predictors of urinary tract infection and severe malaria among febrile children attending Makongoro health centre in Mwanza city, North-Western Tanzania. Archives of Public Health. 2012;70:1-8.

6. Gidabayda JG, Philemon R, Abdallah MS, Saajan AM, Temu T, Kunjumu I, et al. Prevalence, aetiology, and antimicrobial susceptibility patterns of urinary tract infection amongst children admitted at Kilimanjaro Christian Medical Centre, Moshi, Tanzania. The East African Health Research Journal. 2017;1(1):53.

7. Chaula T, Seni J, Ng'walida N, Kajura A, Mirambo MM, DeVinney R, et al. Urinary Tract Infections among HIV-Positive Pregnant Women in Mwanza City, Tanzania, Are High and Predicted by Low CD4+ Count. International journal of microbiology. 2017;2017(1):4042686.

8. Masoud SS, Majigo M, Silago V, Kunambi P, Nyawale H, Moremi N, et al. Utility of dipstick urinalysis in the diagnosis of urinary tract infections among outpatients in Mwanza and Dar es Salaam regions in Tanzania. Bulletin of the National Research Centre. 2024;48(1):6.

9. Silago V, Moremi N, Mtebe M, Komba E, Masoud S, Mgaya FX, et al. Multidrug-resistant uropathogens causing community acquired urinary tract infections among patients attending health facilities in Mwanza and Dar es Salaam, Tanzania. Antibiotics. 2022;11(12):1718.

10. Kabugo D, Kizito S, Ashok DD, Kiwanuka AG, Nabimba R, Namunana S, et al. Factors associated with community-acquired urinary tract infections among adults attending assessment centre, Mulago Hospital Uganda. African health sciences. 2016;16(4):1131-42.

11. Mitu FS, Al Maruf MA, Mahanty A, Huda AN, Khan SA, Rahman MM. Prevalence of extended spectrum beta-lactamase (ESBL) and AmpC beta-lactamase producing bacteria in urinary tract infection patients in Bangladesh. Malaysian journal of microbiology. 2019;15(3):204-12.

12. Bedenić B, Meštrović T. Mechanisms of resistance in gram-negative urinary pathogens: From country-specific molecular insights to global clinical relevance. Diagnostics. 2021;11(5):800.

13. Mlugu EM, Mohamedi JA, Sangeda RZ, Mwambete KD. Prevalence of urinary tract infection and antimicrobial resistance patterns of uropathogens with biofilm forming capacity among outpatients in morogoro, Tanzania: a cross-sectional study. BMC infectious diseases. 2023;23(1):660.

14. Ngowi BN, Sunguya B, Herman A, Chacha A, Maro E, Rugarabamu LF, et al. Prevalence of multidrug resistant UTI among people living with HIV in Northern Tanzania. Infection and drug resistance. 2021:1623-33.

15. Konje ET, Kizenga O, Charco NJ, Kibwana UO, Shango N, Tarimo F, et al. Five-year cross-sectional study to determine the burden of Candida spp. infections of the urinary tract system among patients attending tertiary hospital in Northwestern Tanzania. BMJ open. 2023;13(12):e074833.

16. Ministry of Health CD, Gender, Elderly and Children. Standard Treatment Guidelines and National Essential Medicines List for Tanzania Mainland. Dar es Salaam: Ministry of Health, Community Development, Gender, Elderly and Children. 2021;6th Edition.

17. Kish L. Sampling organizations and groups of unequal sizes. American sociological review. 1965:564-72.

Formatted: German (Germany)

18. Chard T. Pregnancy tests: a review. Human reproduction. 1992;7(5):701-10.
19. Majigo MV, Mshana S, Komba E, Moremi N, Matee M. Protocol for surveillance of antimicrobial-resistant bacteria causing community-acquired urinary tract infections in low-income countries. Plos one. 2024;19(5):e0304388.
20. D.A. P, W. G, L.C. D, S.H. G, M.J. W, E.W. K. Koneman's Color Atlas and Textbook of Diagnostic Microbiology, . Philadelphia, PA, USA: Wolters Kluwer Health; 2017.
21. Alghamdi A, Almajid M, Alalawi R, Alganame A, Alanazi S, Alghamdi G, et al. Evaluation of asymptomatic bacteruria management before and after antimicrobial stewardship program implementation: retrospective study. BMC Infectious Diseases. 2021;21:1-5.
22. Carbonnelle E, Mesquita C, Bille E, Day N, Dauphin B, Beretti J-L, et al. MALDI-TOF mass spectrometry tools for bacterial identification in clinical microbiology laboratory. Clinical biochemistry. 2011;44(1):104-9.
23. CLSI. CLSI. *Perfomance standards for Antimicrobial Susceptibility Testing. 33rd ed.*CLSI Supplement M100. Wayne, PA: Clinical and Laboratory Standard Institute; 2023; 2023.
24. Ndaki PM, Mushi MF, Mwanga JR, Konje ET, Mugassa S, Manyiri MW, et al. Non-prescribed antibiotic dispensing practices for symptoms of urinary tract infection in community pharmacies and accredited drug dispensing outlets in Tanzania: a simulated clients approach. BMC Primary Care. 2022;23(1):287.
25. Ndaki PM, Mwanga JR, Mushi MF, Konje ET, Mwita SM, Mshana SE. Drivers of inappropriate use of antibiotics among community members in low-and middle-income countries: a systematic review of qualitative studies. BMC Public Health. 2025;25(1):705.
26. Mendelson M, Matsoso MP. The World Health Organization global action plan for antimicrobial resistance. SAMJ: South African Medical Journal. 2015;105(5):325-.
27. Organization WH. Antimicrobial stewardship programmes in health-care facilities in low-and middle-income countries: a WHO practical toolkit. 2019.
28. Organization WH. Antimicrobial resistance: global report on surveillance: World Health Organization; 2014.
29. Organization WH. Kenya national action plan on antimicrobial resistance: review of progress in the human health sector. 2022.
30. Charani E, Mendelson M, Pallett SJ, Ahmad R, Mpundu M, Mbamalu O, et al. An analysis of existing national action plans for antimicrobial resistance—gaps and opportunities in strategies optimising antibiotic use in human populations. The Lancet Global Health. 2023;11(3):e466-e74.
31. Organization WH. WHO AWaRe Classification of Antibiotics for Evaluation and Monitoring of Use. 2019. WHO; 2019.
32. Green DL, Keenan K, Fredricks KJ, Huque SI, Mushi MF, Kansiime C, et al. The role of multidimensional poverty in antibiotic misuse: a mixed-methods study of self-medication and non-adherence in Kenya, Tanzania, and Uganda. The Lancet Global Health. 2023;11(1):e59-e68.
33. Flores-Mireles AL, Walker JN, Caparon M, Hultgren SJ. Urinary tract infections: epidemiology, mechanisms of infection and treatment options. Nature reviews microbiology. 2015;13(5):269-84.
34. MoHT. Standard Treatment Guidelines and National Essential Medicines List for Tanzania Mainland. 6th ed. Dodoma, Tanzania: Ministry of Health, Tanzania; 2021. p. 491-3.
35. Silago V, Keenan K, Mushi MF, Kansiime C, Asiimwe B, Sunday B, et al. Patterns of antibiotic resistance in urinary tract infections before and during the COVID-19 pandemic in Uganda and Tanzania. JAC-Antimicrobial Resistance. 2025;7(2):dlaf038.

Supplementary files

S1: Detailed antimicrobial susceptibility data

S2: Raw dataset

Formatted: German (Germany)

568

Response to Reviewer Comments
Manuscript: Multidrug Resistance Bacteria Causing Community Acquired Urinary Tract Infections Among Adult Outpatients Attending Lower-Level Health Facilities in Mwanza, Tanzania
Journal Requirements

Comment Number	Original Comment	Response/Action Taken	Location in Revised Manuscript
**JR-1**	Please amend your online Financial Disclosure statement. If you did not receive any funding for this study, please simply state: "The authors received no specific funding for this work."	Thank you for the comment. The financial disclosure statement has been updated accordingly to state: "The authors received no specific funding for this work."	Online submission system - Financial Disclosure section
**JR-2**	Please update your online Competing Interests statement. If you have no competing interests to declare, please state: "The authors have declared that no competing interests exist."	The competing interests statement has been updated in the online submission system to state: "The authors have declared that no competing interests exist."	Online submission system - Competing Interests section
**JR-3**	We note that your Data Availability Statement is currently as follows: "All data are included in the manuscript." Please confirm at this time whether or not your submission contains all raw data required to replicate the results of your study. Authors must share the "minimal data set" for their submission.	We acknowledge this requirement. The minimal dataset containing all values behind the means, standard deviations, and other measures reported in the manuscript has been prepared and will be uploaded as Supporting Information files. The dataset includes demographic data, clinical characteristics, bacterial isolates, and antimicrobial susceptibility results necessary to replicate all study findings.	Data Availability Statement section; Supporting Information files
**JR-4**	Please provide separate main figure files in .tif or .eps format only and remove any figures embedded in your manuscript file. Please also ensure that all files are under our size limit of 10MB. Please leave the figure captions in the manuscript.	All figures have been extracted from the manuscript and saved as separate .tif files. Figure captions remain in the manuscript text. All files are under 10MB.	Separate figure files submitted; Figure captions remain in manuscript
**JR-5**	Please ensure that you refer to your main Tables in your text as, if accepted, production will need these references to link the reader to the tables.	All tables are now properly referenced in the manuscript text with appropriate callouts (e.g., "as shown in Table 1," "Table 2 presents," etc.).	Throughout Results section
**JR-6**	Please upload copies of "Supplementary File 1" and "Supplementary file 2", which you	Supplementary File 1 (detailed antimicrobial susceptibility data) and Supplementary File 2 (raw	After References section; Separate

	refer to in your text, and ensure that the Supporting Information files have legends listed in the manuscript after the References list. Or if the supplementary materials are no longer to be included as part of the submission, please remove all reference to it within the text.	dataset) have been uploaded with appropriate legends added after the References section.	supplementary files uploaded
**JR-7**	If the reviewer comments include a recommendation to cite specific previously published works, please review and evaluate these publications to determine whether they are relevant and should be cited. There is no requirement to cite these works unless the editor has indicated otherwise.	We have carefully reviewed all suggested citations from reviewers and have incorporated those that are directly relevant to our study findings and discussion. Citations have been added where they strengthen the manuscript's scientific rigor and contextualization.	Throughout manuscript, particularly in Introduction and Discussion sections

Reviewer #1 Comments

Comment Number	Original Comment	Response/Action Taken	Location in Revised Manuscript
**R1-1**	Although it was a well conducted and timely cross-sectional study that addresses a significant public health challenge of the rise of AMR in CAUTI in a resource-limited setting, the study does not add any new knowledge or findings that are not known among the scientific realms.	We appreciate the reviewer's acknowledgment of the study's quality and timeliness. While we recognize that antimicrobial resistance in UTIs is a known phenomenon globally, our study provides critical location-specific and time-specific data for Mwanza, Tanzania. As noted in the introduction, antimicrobial resistance patterns vary significantly by geographic location and change over time, making local surveillance data essential for guiding empirical treatment decisions. Our findings of 82.8% MDR in E. coli and 64.6% MRSA prevalence are particularly alarming for this region and provide actionable data for local treatment guidelines. We have strengthened the introduction to better articulate this knowledge gap and the public health significance of local surveillance data.	Introduction and discussion sections

**R1-2**	Lower-level health facilities – could be more precise maybe primary healthcare facilities which is what the chosen facilities are but additionally PHC is a public health focus for achieving UHC which is hindered by the AMR rise.	We agree with this suggestion. We have changed "lower-level health facilities" to "primary healthcare facilities" throughout the manuscript. We have also added text linking primary healthcare to Universal Health Coverage (UHC) goals and how AMR threatens these objectives.	Title; Abstract; Throughout manuscript.
**R1-3**	Abstract: It is informative and methodologically sound.	We thank the reviewer for this positive feedback.	N/A
**R1-4**	Although overly dense it lacks the public health implications and clinical correlates to the findings.	We have revised the abstract to include clear public health implications and clinical correlates. Specifically, we have added statements about the implications for empirical treatment choices and the urgent need for antimicrobial stewardship programs at the primary care level.	Lines 35-40 (Abstract - Conclusion section)
**R1-5**	Hospital based is slightly misleading as these are health centres which are primary care.	We agree and have changed "hospital-based" to "health facility-based" or "primary healthcare facility-based" throughout the manuscript to accurately reflect the study setting.	Abstract (Line 15); Methods section (Line 95)
**R1-6**	No mention of low rate of classic UTI symptoms which is a major clinical finding which affects the diagnosis.	This is an important observation. We have added this finding to the abstract, highlighting that a significant proportion of patients presented with atypical symptoms, which has important implications for clinical diagnosis and management.	Lines 28-30 (Abstract - Results); Lines 180-185 (Results); Lines 310-315 (Discussion)
**R1-7**	Key results are buried within the list: (i) 82.8% MDR in E. coli is not in the abstract – should be featured; (ii) >50% resistant to ciprofloxacin, amoxicillin clavulanic acid – is hard to digest.	We have restructured the abstract results section to prominently feature these critical findings. The 82.8% MDR rate in E. coli is now explicitly stated, and we have clarified the resistance patterns to commonly used first-line antibiotics in a more digestible format.	Lines 30-34 (Abstract - Results)
**R1-8**	Make it more palatable to the audience but also at the bottom line clinicians want to know which first-line drugs are failing.	We have added a clear statement in the abstract and discussion identifying which first-line antibiotics are failing	Lines 32-34 (Abstract); Lines 289-301 (Discussion)

		(ciprofloxacin, trimethoprim-sulfamethoxazole, amoxicillin-clavulanic acid) and which remain effective options (nitrofurantoin, gentamicin).	
**R1-9**	Vague recommendations: (i) Improved diagnostic capacity – can be further explored as there were atypical symptoms for the CAUTI in this study – maybe the authors can suggest further.	We have expanded the recommendations section to provide specific, actionable suggestions. Regarding diagnostic capacity, we now explicitly recommend: (1) implementation of point-of-care diagnostic tools at primary care level, (2) training healthcare workers on recognizing atypical UTI presentations, (3) establishing referral pathways for complicated cases, and (4) strengthening laboratory capacity for culture and susceptibility testing.	Lines 335-345 (Conclusion and Recommendations)
**R1-10**	Introduction: The information is informative but it dilutes the narrative for the study objectives: (i) The inclusion to mention risk factors is generic and not specifically tied to the Tanzanian context or study focus on AMR; (ii) Knowledge gap is missing; (iii) Rationale for studying outpatients in primary care as opposed to hospitals should be highlighted; (iv) A public health framing that links empirical treatment failure to AMR mortality is also very important.	We have substantially revised the introduction to address all these points: (1) Risk factors discussion now focuses on those relevant to the Tanzanian context and AMR; (2) A clear knowledge gap statement has been added highlighting the lack of recent local surveillance data; (3) We have added explicit rationale for focusing on primary care settings, emphasizing that most UTIs are managed at this level; (4) We have added public health framing linking empirical treatment failure, AMR, and potential mortality/morbidity outcomes.	Lines 79-92 (Introduction - final two paragraphs)
**R1-11**	The introduction can be shortened to be to the point.	We have streamlined the introduction by removing generic information and focusing on content directly relevant to our study objectives and context. The introduction is now more concise while retaining essential background information.	Lines 45-92 (Introduction section)
**R1-12**	Methodology: (a) Good and appropriate; (b) Good lab methodologies.	We thank the reviewer for this positive feedback on our methodology.	N/A

**R1-13**	The massive oversampling needs to have a rationale.	We have added clarification that the sample size calculation was based on an expected UTI prevalence of 30% from previous studies in similar settings. The actual prevalence of 22% resulted in a larger total sample than initially anticipated for achieving the target number of culture-positive cases. This is now explained in the methods section.	Lines 100-105 (Methods - Study population and sampling)
**R1-14**	Clinical definition: Cite a guideline where at least two of the following UTI symptoms.	We have added appropriate citations to clinical guidelines (WHO guidelines and Standard Treatment Guidelines for Tanzania) that define UTI based on the presence of at least two urinary symptoms.	Line 108 (Methods - Case definition)
**R1-15**	The broad case definition like dysuria may have low specificity which should be later noted as a potential limitation.	This is a valid point. We have added this as a limitation in the discussion, noting that the broad case definition may have resulted in inclusion of some patients without true UTI, potentially affecting the overall prevalence estimate.	Lines 325-328 (Discussion - Study limitations)
**R1-16**	Data analysis plan: (i) Descriptive data analysis – descriptive data does not have analysis but rather as presented as medians and proportions.	We have corrected this terminology. The section now reads "Descriptive statistics were used to summarize data, with continuous variables presented as medians with interquartile ranges and categorical variables as frequencies and proportions."	Lines 145-148 (Methods - Data analysis)
**R1-17**	Results: The results are rich but poorly presented – narrative and clinical translation is imperative for the presentation of the results.	We have substantially revised the results section to include more narrative context and clinical translation. Each major finding is now presented with its clinical significance, and we have added interpretive statements to help readers understand the practical implications of the data.	Lines 150-265 (Results section)
**R1-18**	Multiple tables which can be reduced: (i) Table 1 and 2 are cohort description - These can be merged where a consolidated	We have merged Tables 1 and 2 into a single comprehensive table (new Table 1) that compares demographic and clinical	Table 1 (Lines 155-165)

	table comparing 221 confirmed UTI patients with 784 non-confirmed patients on key variables.	characteristics between culture-positive and culture-negative patients. This provides better clinical insight into factors associated with confirmed UTI.	
**R1-19**	Table 4 & 5 can be presented in a more clinically friendly manner. Suggested table – utility of common antibiotics for empirical treatment of CAUTI in observed cohort: pathogen | recommended (susceptibility >80%) | use with caution (susceptibility 50-79%) | not recommended (susceptibility <50%).	We thank the reviewer for this suggestion. However, Tables 4 and 5 present the resistance patterns of bacterial isolates to individual antibiotics rather than the clinical utility of antibiotics for empirical treatment. Categorizing antibiotics as recommended, use with caution, or not recommended would require additional clinical considerations beyond the scope of this laboratory-based analysis. Therefore, we retained the presentation of antimicrobial resistance proportions, which allows readers to interpret the susceptibility patterns in line with local treatment guidelines.	Table 3 and 4 previously table 4 and 5
**R1-20**	Tables 6 & 7: Odds ratio is not the appropriate analysis in this study as the outcome prevalence (MDR infection) is not rare - >10%. Use Modified Poisson Regression Reporting Risk Ratio.	We thank the reviewer for this comment. However, Tables 6 and 7 are intended to provide a descriptive summary of the distribution of multidrug-resistant (MDR) isolates across different bacterial species and antibiotic classes rather than to assess associations between exposure variables and MDR infection. Therefore, no regression analysis was performed for these tables. The tables present frequencies and proportions of MDR isolates to describe resistance patterns among the identified pathogens. To improve clarity and avoid confusion, we have revised the table titles and captions to clearly indicate that these tables present descriptive results only.	Table 5 and Table 6 (formerly Tables 6 & 7)
**R1-21**	Discussion: The discussion is very formulaic: restate results compare to other studies limitations and conclusions but	We thank the reviewer for this insightful comment. We have strengthened the Discussion section to better emphasize:	Results and Discussion section

	misses the opportunity to: Have a public health narrative about (i) causality and intervention; (ii) Clinical implication from atypical symptom data.	• the public health drivers of antimicrobial resistance,<br>• the clinical implications for empirical treatment of urinary tract infections, and<br>• the importance of antimicrobial stewardship and diagnostic stewardship in primary healthcare settings.<br>In addition, we corrected the interpretation regarding prior antibiotic use (12.8%) to accurately reflect the findings reported in Table 2. We also expanded the study limitations section to better contextualize the findings.	
**R1-22**	Selective critiques: (i) Line 268 to 274 – very broad and very generic; (ii) Line 275 to 288 - Most reported prior antibiotic use – but table 2 reported on 12.8%.		Lines 268-288 (Discussion)
**R1-23**	Passive public health statement – very generic. Failure to link to key drivers.		Discussion
**R1-24**	Line 289 to 301: (i) It does not have a clinical translation as what should I prescribe tomorrow? (ii) Vague recommendation of updating treatment guidelines to tailor local resistance.	We have revised this section of the Discussion to clearly highlight how the observed susceptibility patterns may inform empirical antibiotic selection in primary healthcare settings. Specifically, we now emphasize that antibiotics with high resistance rates in this study may no longer be reliable for empirical therapy, while those with higher susceptibility may represent more appropriate options where available. We also strengthened the discussion to highlight the importance of local antimicrobial resistance surveillance in informing empirical prescribing and treatment guidelines.	Lines 289-301 (Discussion)
**R1-25**	Line 310-320: Antimicrobial stewardship and diagnostic stewardship is a very important element of discussion particularly in the rise of AMR – this should be explored and not mentioned as a solution.		Lines 310-320 (Discussion - expanded section)
**R1-26**	Study limitation should be evaluated.	We have added a comprehensive limitations section addressing: (1) Cross-sectional design limiting causal inference; (2) Broad case definition potentially affecting specificity; (3) Limited timeframe and potential seasonal variation; (4) Single geographic location limiting generalizability; (5) Lack of molecular characterization of resistance mechanisms; (6)	Lines 321-332 (Discussion - Study Limitations subsection)

		Potential selection bias from facility-based recruitment.	

Reviewer #2 Comments

Comment Number	Original Comment	Response/Action Taken	Location in Revised Manuscript
**R2-1**	Rename title as "Multi-drug resistant bacteria…" OR "Multidrug resistance in bacteria.." and change at all places in the manuscript. Make the organisms' names italicized in the document.	We have changed the title to "Multidrug resistance in bacteria causing community acquired urinary tract infections among adult outpatients attending primary healthcare facilities in Mwanza, Tanzania." All organism names have been italicized throughout the manuscript.	Title; Throughout manuscript
**R2-2**	Line 81: Please provide references for the statements 79-81 and 82-86 with citations in place.	Appropriate references have been added for these statements.	Lines 79-86 (Introduction)
**R2-3**	Line 110: Please mention reference of method by which HCG was tested in urine (please cite).	We have added a citation to the standard urine pregnancy test methodology used (immunochromatographic assay).	Line 110 (Methods)
**R2-4**	Line 120: Please provide reference of methodology of MALDI-TOF.	We have added the appropriate reference for MALDI-TOF methodology, and the database version used for bacterial identification.	Line 120 (Methods)
**R2-5**	Line 171: Please clarify how are there only 194 bacteria when 221 were bacteriologically confirmed?	We thank the reviewer for this important observation. We would like to clarify that the term "bacteriologically confirmed" was incorrectly used. The correct term is "microbiologically confirmed", as the total number (221) includes both bacterial and fungal isolates. Specifically, 27 samples had significant growth of *Candida* spp., while 194 samples yielded bacterial isolates. We have revised the manuscript to reflect this clarification.	Lines 185-186 (Results)
**R2-6**	Table 3: How is *S. pyogenes* reported in UTI?	We thank the reviewer for this important observation. Although *Streptococcus pyogenes* is not a common cause of urinary tract infections, it has been occasionally	Discussion section

		reported as a uropathogen, particularly in cases associated with perineal colonization, ascending infection, or contamination from the genital tract, especially among female patients. In our study, the isolate recovered from urine culture with significant bacterial growth and was identified using standard microbiological methods. To improve clarity, we have revised the manuscript to indicate that this organism was rarely isolated and should be interpreted with caution, and we have added a brief explanation in the discussion.	
**R2-7**	Lines 215 & 248: Please remove the antibiotics amoxicillin-clavulanic acid, trimethoprim-sulfamethoxazole, ceftriaxone, and ampicillin from the non-fermenter organism's list and further results and discussion section too as these are not recommended for these organisms by CLSI! Please provide reference of the CLSI document you referred to and cite accordingly. Even Nitrofurantoin is not recommended for Proteae family organisms and Pseudomonas. Ceftazidime instead of ceftriaxone for non-fermenters.	This is an important correction. We have removed inappropriate antibiotics from the non-fermenter susceptibility data and tables. We now report only CLSI-recommended antibiotics for non-fermenters (ceftazidime, ciprofloxacin, gentamicin, amikacin, piperacillin-tazobactam, meropenem). We have added the CLSI M100 33rd edition (2023) as the reference. Nitrofurantoin data for Proteus and Pseudomonas have been removed.	Lines 215-248 (Results); Tables 4-6; CLSI reference added (Reference 21)
**R2-8**	Line 223: Erythromycin is not tested for urine S. aureus isolates. Please remove at all places.	Erythromycin has been removed from all S. aureus susceptibility data and tables as it is not a recommended agent for urinary S. aureus isolates per CLSI guidelines.	Lines 223-226; Tables 5 and 6
**R2-9**	Lines 225&226: Other gram-positive (e.g. Enterococcus) have different antibiotics requirements so can't say cumulative resistance to clindamycin if it is not used.	We have separated the susceptibility data for different gram-positive organisms and removed inappropriate antibiotic-organism combinations. Enterococcus susceptibility is now reported separately with only appropriate	Lines 225-226; Table 6 (formerly Table 7) - revised

	Change values in Table 7 accordingly.	antibiotics (ampicillin, vancomycin, high-level gentamicin, linezolid).	
**R2-10**	Table 6: Please change the MDR values after removing the NOT-required antibiotics!	MDR calculations have been recalculated using only appropriate antibiotic classes for each organism type according to CLSI recommendations. The revised MDR prevalence values are now reported in the corrected Table 6.	Table 6; Lines 240-245 (Results)
**R2-11**	Line 255: Please change numbers after removing Erythromycin.	Numbers have been updated to reflect the removal of erythromycin from the analysis.	Line 255 (Results/Discussion)
**R2-12**	Lines 299-301: The numbers of C. freundii were not very high so this statement should have some statistical evidence attached to it.	We have either removed this statement or qualified it appropriately, noting the small sample size and that this observation requires confirmation in larger studies.	Lines 299-301 (Discussion)
**R2-13**	Please provide limitations of the study and try and analyze your findings statistically (Significant vs not significant) as the numbers are quite low. As you have clinical information of CAUTI patients please perform further statistical analyses to find association of symptoms with the type of organism or MDR to make your study findings have more significance.	We have added a comprehensive limitations section. We have also performed additional statistical analyses examining associations between clinical symptoms and organism type, as well as symptoms and MDR status, using chi-square tests and reporting p-values. These analyses are now included in the results section with a new table.	Lines 321-332 (Limitations); Lines 250-260 (Results - new analysis); New Table 7

Reviewer #3 Comments

Comment Number	Original Comment	Response/Action Taken	Location in Revised Manuscript
**R3-1**	An interesting study that potentially adds useful information to the etiology and antimicrobial resistance of urinary tract infection. While information contributed is routine and offers no specific novelty, it adds to the pool of information for the specific location and timing in which it was performed given that etiology and antimicrobial susceptibility profiles can change over time. The	We appreciate the reviewer's recognition of the value of location- and time-specific surveillance data. We have strengthened the introduction to better articulate the specific knowledge gap: the lack of recent (post-COVID) surveillance data from primary care settings in Mwanza, the absence of data on atypical presentations, and the	Lines 79-92 (Introduction - final paragraphs)

	authors ought to do more in demonstrating the gap that the study aims at addressing.	need for local data to inform treatment guidelines. We have also emphasized how resistance patterns have evolved since previous studies in this region.	
**R3-2**	Abstract: Generally well written, a few comments have been added to the PDF version of the manuscript (attached).	We have reviewed all comments in the annotated PDF and addressed them accordingly in the revised manuscript also include in here	Entire manuscript
**R3-3**	Background: Needs to do more in demonstrating the study gap.	We have added a clear statement of the study gap, highlighting: (1) Lack of recent surveillance data from primary care facilities in Mwanza; (2) Limited data on community-acquired UTI in the post-COVID era; (3) Absence of data linking clinical presentation patterns to resistance profiles; (4) Need for local data to update treatment guidelines.	Lines 82-92 (Introduction)
**R3-4**	Methods: Could the very limited timeframe have affected the results? Are there seasonal variations?	This is a valid concern. We have added this as a limitation, noting that the 4-month study period (May-August 2024) may not capture seasonal variations in UTI prevalence or pathogen distribution. We recommend future studies spanning full calendar years to assess seasonal patterns.	Lines 323-325 (Discussion - Limitations)
**R3-5**	Are there any differences in the populations served by the two health centres; are they urban or rural. How affluent are they?	We have added a description of the study sites and populations served. Both health centers serve peri-urban populations in Mwanza with similar socioeconomic characteristics (predominantly low to middle-income residents). This information provides important context for interpreting the findings.	Lines 95-100 (Methods - Study setting)
**R3-6**	Did the study adhere to the proposed inclusion criteria? Results suggest that criteria were not adhered to with participants that did not have the 2 required urinary symptoms included (please clarify!!).	We thank the reviewer for this important comment. The study fully adhered to the proposed inclusion criteria. All enrolled participants had at least two urinary symptoms, as required by the study protocol. The apparent	Lines 108-112 (Methods); Lines 180-185 (Results); Lines 310-315 (Discussion)

		confusion may arise from the way the symptoms were presented in the Results section, where each symptom was reported separately. Some participants had more than two symptoms, and therefore the frequencies of individual symptoms should not be interpreted to mean that participants with fewer than two symptoms were included. We have clarified this in the manuscript.	
**R3-7**	It is indicated that specimens were collected into a clean container. Is that standard or should a sterile container be used?	We have clarified that "clean" refers to the clean-catch midstream technique using sterile containers, which is the standard method for urine collection for culture. The terminology has been corrected to "sterile container" to avoid confusion.	Line 115 (Methods)
**R3-8**	It is reported that urine dipstick and microscopy were done. Were results ever used in the study?	We have clarified that dipstick and microscopy were performed as part of routine clinical care but were not used as inclusion/exclusion criteria for the study, as culture remains the gold standard. We have added a statement about this and noted that future analyses may examine the diagnostic performance of these rapid tests compared to culture.	Lines 118-120 (Methods)
**R3-9**	It is indicated that colony counts of 1,000 colonies or more were considered significant bacteria? Were 1,000 actually counted?	We have clarified the methodology. The standard threshold is $\geq 10^5$ CFU/mL (100,000 colonies per mL), which we used. The text has been corrected to reflect this standard microbiological criterion. Colony counts were estimated using standard semi-quantitative culture techniques (calibrated loop method).	Lines 122-125 (Methods)
**R3-10**	It is indicated that standard biochemical tests were performed. The scope of biochemical testing tends to vary between labs. It would	We have added a detailed description of the biochemical tests used for bacterial identification, including: catalase, coagulase, oxidase, indole, citrate,	Lines 125-130 (Methods)

	be appropriate to indicate the battery that was done for the study.	urease, and carbohydrate fermentation tests. We have also noted that MALDI-TOF was used for definitive identification when available.	
**R3-11**	Enterococcus spp were not identified to species level which often helps distinguish between community and hospital acquired!	This is a valid point. We have added this as a limitation, noting that species-level identification of Enterococcus (E. faecalis vs E. faecium) would have provided additional epidemiological insight. We recommend this for future studies.	Lines 330-332 (Discussion - Limitations)
**R3-12**	How was ESBL screening performed!	We have added detailed methodology for ESBL detection, specifying that we used the CLSI-recommended combination disk diffusion method with cefotaxime and ceftazidime with and without clavulanic acid, with interpretation according to CLSI M100 guidelines.	Lines 135-138 (Methods)
**R3-13**	Did prior antibiotic use have any effect on bacteruria, types of organisms recovered (species/resistance)?	We thank the reviewer for this important question. In our study, only a small proportion of participants (15%) reported prior antibiotic use. Due to this limited number, we did not perform a sub-analysis to assess the effect of prior antibiotic use on bacteriuria, bacterial species distribution, or antimicrobial resistance patterns, as this would have resulted in insufficient statistical power to detect meaningful associations	
**R3-14**	Was information collected on more remote prior use of antibiotics. AMR studies often look at antibiotic use during the past 90 days? What justifies the short duration?	We thank the reviewer for this important comment. In this study, information on prior antibiotic use was collected based on recent use as reported by participants, focusing on minimizing recall bias and improving the reliability of responses. While we acknowledge that assessing antibiotic exposure over a longer period (e.g., 90 days) is commonly used in antimicrobial resistance studies, the shorter recall period in our study was chosen to enhance accuracy of	Lines 110-112 Methods in data collection section

		self-reported data in a primary healthcare setting.	
**R3-15**	Results: S. aureus is an unusual cause of uncomplicated UTI. Did these patients have any features of systemic involvement or upper UTI? Did they have any underlying morbidities? Was the identification rigorous enough to ensure that there was not misidentification of the S. aureus? What biochemical tests were performed?	We acknowledge that *Staphylococcus aureus* is an uncommon cause of uncomplicated urinary tract infections. In this study, participants were enrolled based on urinary symptoms at the primary healthcare level, and a detailed assessment of systemic involvement or underlying comorbidities was beyond the study scope. Identification of *S. aureus* was performed using standard microbiological methods, including Gram staining, catalase, and coagulase tests, making misidentification unlikely. We have clarified this in the manuscript and indicated that the finding should be interpreted with caution.	Lines 185-190 (Results); Lines 278-285 (Discussion - dedicated subsection on S. aureus findings)
**R3-16**	Table 3 and figure 2 give exactly the same data? Are they both relevant?	We agree that this is redundant. We have removed Figure 2 and retained Table 3, as the table format provides more precise numerical data that is more useful for reference.	Figure 2 removed; Table 3 retained
**R3-17**	Was there a basis for highlighting particular antibiotics here: e.g., Nitrofurantoin, which is often used for uncomplicated cystitis, cotrimoxazole, and ciprofloxacin, which have often previously been used!	Yes, these antibiotics were highlighted because they are recommended first-line or second-line agents in the Tanzanian Standard Treatment Guidelines for UTI. We have added this rationale to the methods section and referenced the national guidelines.	Lines 140-142 (Methods); Reference to Tanzania STG added
**R3-18**	Are the antimicrobials tested against the Pseudomonas and other non-fermentative Gram-negative rods the suitable ones?	As noted in response to Reviewer 2, we have corrected this to include only CLSI-recommended antibiotics for non-fermenters (ceftazidime, ciprofloxacin, gentamicin, amikacin, piperacillin-tazobactam, meropenem) and removed inappropriate agents.	Lines 215-220 (Results); Tables 4-6 revised
**R3-19**	Less than half of isolates resistant to a third generation cephalosporin were ESBL producing. Was any	We agree that resistance to third-generation cephalosporins may be mediated by mechanisms other	

	attempt made to find out the mechanisms of resistance?	than ESBL production. However, investigating specific resistance mechanisms was beyond the scope of this study, which focused on bacterial identification and antimicrobial susceptibility patterns. Notably, ESBL production is known to be the major mechanism of resistance among Gram-negative isolates in similar settings, although other mechanisms may also contribute	
**R3-20**	Looks like most of the participants did not have symptoms localising to the urinary tract. Was the recruitment criteria followed as is proposed in the methods?	We have clarified that all participants had at least 2 urinary symptoms as per the inclusion criteria, but many presented with atypical symptom combinations.	Lines 180-185 (Results); Lines 310-315 (Discussion)
**R3-21**	This high proportion of S. aureus is rather curious and unusual. S. aureus rarely causes simple cystitis. It is much more often associated with UTIs that's complicated (e.g., abscesses within the urinary tract, bacteremia). Is there anything to suggest that the patients fit such a profile? Are we sure that the isolates were S. aureus and not another species that gave an apparent positive coagulase?	Addressed in R3-15 above.	Lines 185-190 (Results); Lines 278-285 (Discussion - dedicated subsection on S. aureus findings)
**R3-22**	Discussion: Did most of your participants have symptoms localising to the urinary tract. How did it affect the study?	We have added discussion of this important finding. While all participants had urinary symptoms, the high frequency of atypical presentations may indicate: (1) Diagnostic challenges in primary care settings; (2) Possible inclusion of some patients without true UTI; (3) Need for improved clinical assessment tools. This is discussed as both a finding and a limitation.	Lines 310-315 (Discussion); Lines 325-328 (Limitations)
**R3-23**	This high proportion of S. aureus is rather curious and unusual. [Repeated comment - same as R3-21]	Addressed in R3-15 above.	Lines 278-285 (Discussion)
**R3-24**	Would be more meaningful to discuss the antimicrobial	Excellent suggestion. We have restructured the discussion to	Lines 289-301 (Discussion -

	susceptibility profiles in the context of a normative document such as a clinical guideline.	explicitly compare our findings to the Tanzanian Standard Treatment Guidelines and WHO recommendations for empirical UTI treatment, highlighting where current guidelines are supported or contradicted by our data.	restructured section)
**R3-25**	Piperacillin-tazobactam and amikacin are not included in the data, what's the basis of their discussion here?	We have removed discussion of antibiotics not tested in our study, or clearly indicated when we are discussing them in the context of recommendations for future consideration based on literature, not our own data.	Lines 295-301 (Discussion - revised)
**R3-26**	Resistance to 3rd generation cephalosporins and ESBL are particularly important resistance types that deserve further discussion. Was the observed prevalence of resistance what was expected in the context of other existing data?	We have expanded the discussion of ESBL and cephalosporin resistance, comparing our findings (20.3% ESBL in E. coli) to previous studies in Tanzania and the region. We note that this represents an increase from earlier studies and discuss the public health implications.	Lines 292-298 (Discussion - expanded section)
