# Supplementary material for: Multidrug resistance in bacteria causing community-acquired urinary tract infections among adult outpatients attending primary healthcare facilities in Mwanza, Tanzania
Source: PLOS Glob Public Health. 2026 Oct 8;6(10):e0005638. doi: 10.1371/journal.pgph.0005638 (PMC13649018; doi:10.1371/journal.pgph.0005638)
Supplement: Supplementary file 3 [file pgph.0005638.s003.docx]

**Manuscript Review**

**Title:**	Multidrug resistant bacteria causing community acquired urinary tract infections

among adult outpatients at lower-level health facilities in Mwanza, Tanzania

**Reviewer:**	Henry Kajumbula

| **Aspect** | **Comment** |
| --- | --- |
| ***General observation*** | - An interesting study that potentially adds useful information to the etiology and antimicrobial resistance of urinary tract infection   - While information contributed is routine and offers no specific novelty. It adds to the pool of information for the specific location and timing in which it was performed given that etiology and antimicrobial susceptibility profiles can change over time.   - The authors ought to do more in demonstrating the gap that the study aims at addressing |
| ***Abstract*** | - Generally well written, a few comments have been added to the PDF version of the manuscript (attached) |
| ***Background*** | - Needs to do more in demonstrating the study gap |
| ***Methods*** | - Could the very limited timeframe have affected the results? Are there seasonal variation? - Are there any differences in the populations served by the two health centres; are they urban or rural. How affluent are they? - Did the study adhere to the proposed inclusion criteria? Results suggest that criteria were not adhered to with participants that did not have the 2 required urinary symptoms included (please clarify!!) - It is indicated that specimens were collected into a clean container. Is that standard or should a sterile container be used - It is reported that urine dipstick and microscopy were done. Were results ever used in the study? - It is indicated that colony counts of 1,000 colonies or more were considered significant bacteria? Were 1,000 actually counted? - It is indicated that standard biochemical test were performed. The scope of biochemical testing tends to vary between labs. It would be appropriate to indicate the batter that was done for the study. - Enterococcus spp were not identified to species level which often helps distinguish between community and hospital acquired! - How was ESBL screening performed! - Did prior antibiotic use have any effect on bacteruria, types of organisms recovered (species/resistance)? - Was information collected on more remote prior use of antibiotics. AMR studies often look at antibiotic use during the past 90 days? What justifies the short duration |
| ***Results*** | *S.aureus* is an unusual cause of uncomplicated UTI. Did these patients have any features of systemic involvement or upper UTI? Did they have any underlying morbidities? Was the identification rigorous enough to ensure that there was not misidentification of the *S.aureus*? What biochemical tests were performed?  Table 3 and figure 2 give exactly the same data? Are they both relevant?  Was there a basis for highlighting particular antibiotics here: e.g., Nitrofurantoin which is often used for uncomplicated cystis, cotrimoxazole, ciprofloxacin which have often previously been used!  Are the antimicrobials tested against the Pseudomonas and other non-fermentative Gram-negative rods the suitable ones?  Less than half of isolates resistant to a third generation cephalosporin were ESBL producing. Was any attempt made to find out the mechanisms of resistance?  Looks like most of the participants did not have symptoms localising to the urinary tract. Was the recruitment criteria followed as is proposed in the methods?  This high proportion of *S.aureus* is rather curious and unusual. *S.aureus* rarely causes simple cystitis. It is much more often associated with UTIs that's complicated (e.g., abscesses within the urinary tract, bacteremia). Is there anything to suggest that the patients fit such a profile? Are we sure that the isolates were *S.aureus* and not another species that gave an apparent positive coagulase? |
| ***Discussion*** | Did most of your participants have symptoms localising to the urinary tract. How did it affect the study?  This high proportion of S.aureus is rather curious and unusual. S.aureus rarely causes simple cystitis. It is much more often associated with UTIs that's complicated (e.g., abscesses within the urinary tract, bacteremia). Is there anything to suggest that the patients fit such a profile? Are we sure that the isolates were S.aureus and not another species that gave an apparent positive coagulase?  Would be more meaningful to discuss the antimicrobial susceptibility profiles in the context of a normative document such as a clinical guideline.  Piperacillin-tazobactam and amikacin are not included in the data, what's the basis of their discussion here?  Resistance to 3rd generation cephalosporins and ESBL are particularly important resistance types that deserve further discussion. Was the observed prevalence of resistance what was expected in the context of other existing data? |
