# Supplementary material for: Multidrug resistance in bacteria causing community-acquired urinary tract infections among adult outpatients attending primary healthcare facilities in Mwanza, Tanzania
Source: PLOS Glob Public Health. 2026 Oct 8;6(10):e0005638. doi: 10.1371/journal.pgph.0005638 (PMC13649018; doi:10.1371/journal.pgph.0005638)
Supplement: Supplementary file 4 [file pgph.0005638.s007.docx]

Review for manuscript titled: “Multidrug resistance bacteria causing community acquired urinary tract infections among adult outpatients attending lower-level health facilities in Mwanza, Tanzania” by Martha F mushi et al.

General feedback:
The study looks more like it was conducted retrospectively with whatever data was available rather than prospectively with definite aims and objectives and does have some technical knowledge gaps. Moreover, the public health implications mentioned are general and not solely based on this study.

The study participants were included with probable UTI but later very few show symptoms, so how were these patients identified in the OPD? Please elaborate. Please add the methodology of ESBL detection. Do follow CLSI guidelines (as cited) for antibiotic testing for various microorganisms.

Abstract: Please keep all scientific name italicized e.g., *Escherichia coli*

Some of the suggestions previously made were still not addressed!

Line 195, Table 2: *Streptococcus pyogenes* is not commonly reported in urine. Please check and confirm the identity or do not include.

Lines 207-209: You mention 20% ESBL but no mention of third-generation cephalosporin resistance.

Line 212: How were ESBLs detected? Please elaborate in methodology.

Lines 213-214: You mention resistance of *Pseudomonas* to Ampicillin, amoxy-clav and ceftriaxone but these are NOT the recommended antibiotics for *Pseudomonas* as the organism has intrinsic resistance towards these antibiotics.

Line 216 and 219: Erythromycin is not to be reported for urinary isolates of Gram negative organisms or *S.aureus*. You can use Erythromycin to screen for Macrolide-Lincosamide resistance using the D-test and mention the details in methodology but exclude it from resistance and MDR analyses.

Line 223, Table 3: Nitrofurantoin is not applicable for *Pseudomonas*.

Lines 322-323: As you have collected the co-morbidities data, you should analyze the correlation.

Lines 370-374: For the public health implications to be true, the information regarding current status has to be provided. Whenever providing conclusions or recommendations, please limit it to your study findings. If your study had provided local treatment guideline details and then compared with the empiric treatment in these canters, then only can you conclude that this is the public health implication.

Lines 384-388: You can’t generalize the comments, they have to be observed in your study and then only discussed.
