# Supplementary material for: Multidrug resistance in bacteria causing community-acquired urinary tract infections among adult outpatients attending primary healthcare facilities in Mwanza, Tanzania
Source: PLOS Glob Public Health. 2026 Oct 8;6(10):e0005638. doi: 10.1371/journal.pgph.0005638 (PMC13649018; doi:10.1371/journal.pgph.0005638)
Supplement: Supplementary file 5 [file pgph.0005638.s008.docx]

# Response to Reviewer Comments

## Manuscript: Multidrug Resistance Bacteria Causing Community Acquired Urinary Tract Infections Among Adult Outpatients Attending Lower-Level Health Facilities in Mwanza, Tanzania

## Journal Requirements

| Comment Number | Original Comment | Response/Action Taken | Location in Revised Manuscript |
| --- | --- | --- | --- |
| **JR-1** | Please amend your online Financial Disclosure statement. If you did not receive any funding for this study, please simply state: “The authors received no specific funding for this work.” | Thank you for the comment. The financial disclosure statement has been updated accordingly to state: “The authors received no specific funding for this work.” | Online submission system - Financial Disclosure section |
| **JR-2** | Please update your online Competing Interests statement. If you have no competing interests to declare, please state: “The authors have declared that no competing interests exist.” | The competing interests statement has been updated in the online submission system to state: “The authors have declared that no competing interests exist.” | Online submission system - Competing Interests section |
| **JR-3** | We note that your Data Availability Statement is currently as follows: “All data are included in the manuscript.” Please confirm at this time whether or not your submission contains all raw data required to replicate the results of your study. Authors must share the “minimal data set” for their submission. | We acknowledge this requirement. The minimal dataset containing all values behind the means, standard deviations, and other measures reported in the manuscript has been prepared and will be uploaded as Supporting Information files. The dataset includes demographic data, clinical characteristics, bacterial isolates, and antimicrobial susceptibility results necessary to replicate all study findings. | Data Availability Statement section; Supporting Information files |
| **JR-4** | Please provide separate main figure files in .tif or .eps format only and remove any figures embedded in your manuscript file. Please also ensure that all files are under our size limit of 10MB. Please leave the figure captions in the manuscript. | All figures have been extracted from the manuscript and saved as separate .tif files. Figure captions remain in the manuscript text. All files are under 10MB. | Separate figure files submitted; Figure captions remain in manuscript |
| **JR-5** | Please ensure that you refer to your main Tables in your text as, if accepted, production will need these references to link the reader to the tables. | All tables are now properly referenced in the manuscript text with appropriate callouts (e.g., “as shown in Table 1,” “Table 2 presents,” etc.). | Throughout Results section |
| **JR-6** | Please upload copies of “Supplementary File 1” and “Supplementary file 2”, which you refer to in your text, and ensure that the Supporting Information files have legends listed in the manuscript after the References list. Or if the supplementary materials are no longer to be included as part of the submission, please remove all reference to it within the text. | Supplementary File 1 (detailed antimicrobial susceptibility data) and Supplementary File 2 (raw dataset) have been uploaded with appropriate legends added after the References section. | After References section; Separate supplementary files uploaded |
| **JR-7** | If the reviewer comments include a recommendation to cite specific previously published works, please review and evaluate these publications to determine whether they are relevant and should be cited. There is no requirement to cite these works unless the editor has indicated otherwise. | We have carefully reviewed all suggested citations from reviewers and have incorporated those that are directly relevant to our study findings and discussion. Citations have been added where they strengthen the manuscript’s scientific rigor and contextualization. | Throughout manuscript, particularly in Introduction and Discussion sections |

## Reviewer #1 Comments

| Comment Number | Original Comment | Response/Action Taken | Location in Revised Manuscript |
| --- | --- | --- | --- |
| **R1-1** | Although it was a well conducted and timely cross-sectional study that addresses a significant public health challenge of the rise of AMR in CAUTI in a resource-limited setting, the study does not add any new knowledge or findings that are not known among the scientific realms. | We appreciate the reviewer’s acknowledgment of the study’s quality and timeliness. While we recognize that antimicrobial resistance in UTIs is a known phenomenon globally, our study provides critical location-specific and time-specific data for Mwanza, Tanzania. As noted in the introduction, antimicrobial resistance patterns vary significantly by geographic location and change over time, making local surveillance data essential for guiding empirical treatment decisions. Our findings of 82.8% MDR in E. coli and 64.6% MRSA prevalence are particularly alarming for this region and provide actionable data for local treatment guidelines. We have strengthened the introduction to better articulate this knowledge gap and the public health significance of local surveillance data. | Introduction and discussion sections |
| **R1-2** | Lower-level health facilities – could be more precise maybe primary healthcare facilities which is what the chosen facilities are but additionally PHC is a public health focus for achieving UHC which is hindered by the AMR rise. | We agree with this suggestion. We have changed “lower-level health facilities” to “primary healthcare facilities” throughout the manuscript. We have also added text linking primary healthcare to Universal Health Coverage (UHC) goals and how AMR threatens these objectives. | Title; Abstract; Throughout manuscript. |
| **R1-3** | Abstract: It is informative and methodologically sound. | We thank the reviewer for this positive feedback. | N/A |
| **R1-4** | Although overly dense it lacks the public health implications and clinical correlates to the findings. | We have revised the abstract to include clear public health implications and clinical correlates. Specifically, we have added statements about the implications for empirical treatment choices and the urgent need for antimicrobial stewardship programs at the primary care level. | Lines 35-40 (Abstract - Conclusion section) |
| **R1-5** | Hospital based is slightly misleading as these are health centres which are primary care. | We agree and have changed “hospital-based” to “health facility-based” or “primary healthcare facility-based” throughout the manuscript to accurately reflect the study setting. | Abstract (Line 15); Methods section (Line 95) |
| **R1-6** | No mention of low rate of classic UTI symptoms which is a major clinical finding which affects the diagnosis. | This is an important observation. We have added this finding to the abstract, highlighting that a significant proportion of patients presented with atypical symptoms, which has important implications for clinical diagnosis and management. | Lines 28-30 (Abstract - Results); Lines 180-185 (Results); Lines 310-315 (Discussion) |
| **R1-7** | Key results are buried within the list: (i) 82.8% MDR in E. coli is not in the abstract – should be featured; (ii) >50% resistant to ciprofloxacin, amoxicillin clavulanic acid – is hard to digest. | We have restructured the abstract results section to prominently feature these critical findings. The 82.8% MDR rate in E. coli is now explicitly stated, and we have clarified the resistance patterns to commonly used first-line antibiotics in a more digestible format. | Lines 30-34 (Abstract - Results) |
| **R1-8** | Make it more palatable to the audience but also at the bottom line clinicians want to know which first-line drugs are failing. | We have added a clear statement in the abstract and discussion identifying which first-line antibiotics are failing (ciprofloxacin, trimethoprim-sulfamethoxazole, amoxicillin-clavulanic acid) and which remain effective options (nitrofurantoin, gentamicin). | Lines 32-34 (Abstract); Lines 289-301 (Discussion) |
| **R1-9** | Vague recommendations: (i) Improved diagnostic capacity – can be further explored as there were atypical symptoms for the CAUTI in this study – maybe the authors can suggest further. | We have expanded the recommendations section to provide specific, actionable suggestions. Regarding diagnostic capacity, we now explicitly recommend: (1) implementation of point-of-care diagnostic tools at primary care level, (2) training healthcare workers on recognizing atypical UTI presentations, (3) establishing referral pathways for complicated cases, and (4) strengthening laboratory capacity for culture and susceptibility testing. | Lines 335-345 (Conclusion and Recommendations) |
| **R1-10** | Introduction: The information is informative but it dilutes the narrative for the study objectives: (i) The inclusion to mention risk factors is generic and not specifically tied to the Tanzanian context or study focus on AMR; (ii) Knowledge gap is missing; (iii) Rationale for studying outpatients in primary care as opposed to hospitals should be highlighted; (iv) A public health framing that links empirical treatment failure to AMR mortality is also very important. | We have substantially revised the introduction to address all these points: (1) Risk factors discussion now focuses on those relevant to the Tanzanian context and AMR; (2) A clear knowledge gap statement has been added highlighting the lack of recent local surveillance data; (3) We have added explicit rationale for focusing on primary care settings, emphasizing that most UTIs are managed at this level; (4) We have added public health framing linking empirical treatment failure, AMR, and potential mortality/morbidity outcomes. | Lines 79-92 (Introduction - final two paragraphs) |
| **R1-11** | The introduction can be shortened to be to the point. | We have streamlined the introduction by removing generic information and focusing on content directly relevant to our study objectives and context. The introduction is now more concise while retaining essential background information. | Lines 45-92 (Introduction section) |
| **R1-12** | Methodology: (a) Good and appropriate; (b) Good lab methodologies. | We thank the reviewer for this positive feedback on our methodology. | N/A |
| **R1-13** | The massive oversampling needs to have a rationale. | We have added clarification that the sample size calculation was based on an expected UTI prevalence of 30% from previous studies in similar settings. The actual prevalence of 22% resulted in a larger total sample than initially anticipated for achieving the target number of culture-positive cases. This is now explained in the methods section. | Lines 100-105 (Methods - Study population and sampling) |
| **R1-14** | Clinical definition: Cite a guideline where at least two of the following UTI symptoms. | We have added appropriate citations to clinical guidelines (WHO guidelines and Standard Treatment Guidelines for Tanzania) that define UTI based on the presence of at least two urinary symptoms. | Line 108 (Methods - Case definition) |
| **R1-15** | The broad case definition like dysuria may have low specificity which should be later noted as a potential limitation. | This is a valid point. We have added this as a limitation in the discussion, noting that the broad case definition may have resulted in inclusion of some patients without true UTI, potentially affecting the overall prevalence estimate. | Lines 325-328 (Discussion - Study limitations) |
| **R1-16** | Data analysis plan: (i) Descriptive data analysis – descriptive data does not have analysis but rather as presented as medians and proportions. | We have corrected this terminology. The section now reads “Descriptive statistics were used to summarize data, with continuous variables presented as medians with interquartile ranges and categorical variables as frequencies and proportions.” | Lines 145-148 (Methods - Data analysis) |
| **R1-17** | Results: The results are rich but poorly presented – narrative and clinical translation is imperative for the presentation of the results. | We have substantially revised the results section to include more narrative context and clinical translation. Each major finding is now presented with its clinical significance, and we have added interpretive statements to help readers understand the practical implications of the data. | Lines 150-265 (Results section) |
| **R1-18** | Multiple tables which can be reduced: (i) Table 1 and 2 are cohort description - These can be merged where a consolidated table comparing 221 confirmed UTI patients with 784 non-confirmed patients on key variables. | We have merged Tables 1 and 2 into a single comprehensive table (new Table 1) that compares demographic and clinical characteristics between culture-positive and culture-negative patients. This provides better clinical insight into factors associated with confirmed UTI. | Table 1 (Lines 155-165) |
| **R1-19** | Table 4 & 5 can be presented in a more clinically friendly manner. Suggested table – utility of common antibiotics for empirical treatment of CAUTI in observed cohort: pathogen \| recommended (susceptibility >80%) \| use with caution (susceptibility 50-79%) \| not recommended (susceptibility <50%). | We thank the reviewer for this suggestion. However, Tables 4 and 5 present the resistance patterns of bacterial isolates to individual antibiotics rather than the clinical utility of antibiotics for empirical treatment. Categorizing antibiotics as recommended, use with caution, or not recommended would require additional clinical considerations beyond the scope of this laboratory-based analysis. Therefore, we retained the presentation of antimicrobial resistance proportions, which allows readers to interpret the susceptibility patterns in line with local treatment guidelines. | Table 3 and 4 previously table 4 and 5 |
| **R1-20** | Tables 6 & 7: Odds ratio is not the appropriate analysis in this study as the outcome prevalence (MDR infection) is not rare - >10%. Use Modified Poisson Regression Reporting Risk Ratio. | We thank the reviewer for this comment. However, Tables 6 and 7 are intended to provide a descriptive summary of the distribution of multidrug-resistant (MDR) isolates across different bacterial species and antibiotic classes rather than to assess associations between exposure variables and MDR infection. Therefore, no regression analysis was performed for these tables. The tables present frequencies and proportions of MDR isolates to describe resistance patterns among the identified pathogens. To improve clarity and avoid confusion, we have revised the table titles and captions to clearly indicate that these tables present descriptive results only. | Table 5 and Table 6 (formerly Tables 6 & 7) |
| **R1-21** | Discussion: The discussion is very formulaic: restate results compare to other studies limitations and conclusions but misses the opportunity to: Have a public health narrative about (i) causality and intervention; (ii) Clinical implication from atypical symptom data. | We thank the reviewer for this insightful comment. We have strengthened the Discussion section to better emphasize:   - the public health drivers of antimicrobial resistance, - the clinical implications for empirical treatment of urinary tract infections, and - the importance of antimicrobial stewardship and diagnostic stewardship in primary healthcare settings.   In addition, we corrected the interpretation regarding prior antibiotic use (12.8%) to accurately reflect the findings reported in Table 2. We also expanded the study limitations section to better contextualize the findings. | Results and Discussion section |
| **R1-22** | Selective critiques: (i) Line 268 to 274 – very broad and very generic; (ii) Line 275 to 288 - Most reported prior antibiotic use – but table 2 reported on 12.8%. |  | Lines 268-288 (Discussion) |
| **R1-23** | Passive public health statement – very generic. Failure to link to key drivers. |  | Discussion |
| **R1-24** | Line 289 to 301: (i) It does not have a clinical translation as what should I prescribe tomorrow? (ii) Vague recommendation of updating treatment guidelines to tailor local resistance. | We have revised this section of the Discussion to clearly highlight how the observed susceptibility patterns may inform empirical antibiotic selection in primary healthcare settings. Specifically, we now emphasize that antibiotics with high resistance rates in this study may no longer be reliable for empirical therapy, while those with higher susceptibility may represent more appropriate options where available. We also strengthened the discussion to highlight the importance of local antimicrobial resistance surveillance in informing empirical prescribing and treatment guidelines. | Lines 289-301 (Discussion) |
| **R1-25** | Line 310-320: Antimicrobial stewardship and diagnostic stewardship is a very important element of discussion particularly in the rise of AMR – this should be explored and not mentioned as a solution. |  | Lines 310-320 (Discussion - expanded section) |
| **R1-26** | Study limitation should be evaluated. | We have added a comprehensive limitations section addressing: (1) Cross-sectional design limiting causal inference; (2) Broad case definition potentially affecting specificity; (3) Limited timeframe and potential seasonal variation; (4) Single geographic location limiting generalizability; (5) Lack of molecular characterization of resistance mechanisms; (6) Potential selection bias from facility-based recruitment. | Lines 321-332 (Discussion - Study Limitations subsection) |

## Reviewer #2 Comments

| Comment Number | Original Comment | Response/Action Taken | Location in Revised Manuscript |
| --- | --- | --- | --- |
| **R2-1** | Rename title as “Multi-drug resistant bacteria…” OR “Multidrug resistance in bacteria..” and change at all places in the manuscript. Make the organisms’ names italicized in the document. | We have changed the title to “Multidrug resistance in bacteria causing community acquired urinary tract infections among adult outpatients attending primary healthcare facilities in Mwanza, Tanzania.” All organism names have been italicized throughout the manuscript. | Title; Throughout manuscript |
| **R2-2** | Line 81: Please provide references for the statements 79-81 and 82-86 with citations in place. | Appropriate references have been added for these statements. | Lines 79-86 (Introduction) |
| **R2-3** | Line 110: Please mention reference of method by which HCG was tested in urine (please cite). | We have added a citation to the standard urine pregnancy test methodology used (immunochromatographic assay). | Line 110 (Methods) |
| **R2-4** | Line 120: Please provide reference of methodology of MALDI-TOF. | We have added the appropriate reference for MALDI-TOF methodology, and the database version used for bacterial identification. | Line 120 (Methods) |
| **R2-5** | Line 171: Please clarify how are there only 194 bacteria when 221 were bacteriologically confirmed? | We thank the reviewer for this important observation. We would like to clarify that the term “bacteriologically confirmed” was incorrectly used. The correct term is “microbiologically confirmed”, as the total number (221) includes both bacterial and fungal isolates. Specifically, 27 samples had significant growth of *Candida* spp., while 194 samples yielded bacterial isolates. We have revised the manuscript to reflect this clarification. | Lines 185-186 (Results) |
| **R2-6** | Table 3: How is *S. pyogenes* reported in UTI? | We thank the reviewer for this important observation. Although *Streptococcus pyogenes* is not a common cause of urinary tract infections, it has been occasionally reported as a uropathogen, particularly in cases associated with perineal colonization, ascending infection, or contamination from the genital tract, especially among female patients. In our study, the isolate recovered from urine culture with significant bacterial growth and was identified using standard microbiological methods. To improve clarity, we have revised the manuscript to indicate that this organism was rarely isolated and should be interpreted with caution, and we have added a brief explanation in the discussion. | Discussion section |
| **R2-7** | Lines 215 & 248: Please remove the antibiotics amoxicillin-clavulanic acid, trimethoprim-sulfamethoxazole, ceftriaxone, and ampicillin from the non-fermenter organism’s list and further results and discussion section too as these are not recommended for these organisms by CLSI! Please provide reference of the CLSI document you referred to and cite accordingly. Even Nitrofurantoin is not recommended for Proteae family organisms and Pseudomonas. Ceftazidime instead of ceftriaxone for non-fermenters. | This is an important correction. We have removed inappropriate antibiotics from the non-fermenter susceptibility data and tables. We now report only CLSI-recommended antibiotics for non-fermenters (ceftazidime, ciprofloxacin, gentamicin, amikacin, piperacillin-tazobactam, meropenem). We have added the CLSI M100 33rd edition (2023) as the reference. Nitrofurantoin data for Proteus and Pseudomonas have been removed. | Lines 215-248 (Results); Tables 4-6; CLSI reference added (Reference 21) |
| **R2-8** | Line 223: Erythromycin is not tested for urine S. aureus isolates. Please remove at all places. | Erythromycin has been removed from all S. aureus susceptibility data and tables as it is not a recommended agent for urinary S. aureus isolates per CLSI guidelines. | Lines 223-226; Tables 5 and 6 |
| **R2-9** | Lines 225&226: Other gram-positive (e.g. Enterococcus) have different antibiotics requirements so can’t say cumulative resistance to clindamycin if it is not used. Change values in Table 7 accordingly. | We have separated the susceptibility data for different gram-positive organisms and removed inappropriate antibiotic-organism combinations. Enterococcus susceptibility is now reported separately with only appropriate antibiotics (ampicillin, vancomycin, high-level gentamicin, linezolid). | Lines 225-226; Table 6 (formerly Table 7) - revised |
| **R2-10** | Table 6: Please change the MDR values after removing the NOT-required antibiotics! | MDR calculations have been recalculated using only appropriate antibiotic classes for each organism type according to CLSI recommendations. The revised MDR prevalence values are now reported in the corrected Table 6. | Table 6; Lines 240-245 (Results) |
| **R2-11** | Line 255: Please change numbers after removing Erythromycin. | Numbers have been updated to reflect the removal of erythromycin from the analysis. | Line 255 (Results/Discussion) |
| **R2-12** | Lines 299-301: The numbers of C. freundii were not very high so this statement should have some statistical evidence attached to it. | We have either removed this statement or qualified it appropriately, noting the small sample size and that this observation requires confirmation in larger studies. | Lines 299-301 (Discussion) |
| **R2-13** | Please provide limitations of the study and try and analyze your findings statistically (Significant vs not significant) as the numbers are quite low. As you have clinical information of CAUTI patients please perform further statistical analyses to find association of symptoms with the type of organism or MDR to make your study findings have more significance. | We have added a comprehensive limitations section. We have also performed additional statistical analyses examining associations between clinical symptoms and organism type, as well as symptoms and MDR status, using chi-square tests and reporting p-values. These analyses are now included in the results section with a new table. | Lines 321-332 (Limitations); Lines 250-260 (Results - new analysis); New Table 7 |

## Reviewer #3 Comments

| Comment Number | Original Comment | Response/Action Taken | Location in Revised Manuscript |
| --- | --- | --- | --- |
| **R3-1** | An interesting study that potentially adds useful information to the etiology and antimicrobial resistance of urinary tract infection. While information contributed is routine and offers no specific novelty, it adds to the pool of information for the specific location and timing in which it was performed given that etiology and antimicrobial susceptibility profiles can change over time. The authors ought to do more in demonstrating the gap that the study aims at addressing. | We appreciate the reviewer’s recognition of the value of location- and time-specific surveillance data. We have strengthened the introduction to better articulate the specific knowledge gap: the lack of recent (post-COVID) surveillance data from primary care settings in Mwanza, the absence of data on atypical presentations, and the need for local data to inform treatment guidelines. We have also emphasized how resistance patterns have evolved since previous studies in this region. | Lines 79-92 (Introduction - final paragraphs) |
| **R3-2** | Abstract: Generally well written, a few comments have been added to the PDF version of the manuscript (attached). | We have reviewed all comments in the annotated PDF and addressed them accordingly in the revised manuscript also include in here | Entire manuscript |
| **R3-3** | Background: Needs to do more in demonstrating the study gap. | We have added a clear statement of the study gap, highlighting: (1) Lack of recent surveillance data from primary care facilities in Mwanza; (2) Limited data on community-acquired UTI in the post-COVID era; (3) Absence of data linking clinical presentation patterns to resistance profiles; (4) Need for local data to update treatment guidelines. | Lines 82-92 (Introduction) |
| **R3-4** | Methods: Could the very limited timeframe have affected the results? Are there seasonal variations? | This is a valid concern. We have added this as a limitation, noting that the 4-month study period (May-August 2024) may not capture seasonal variations in UTI prevalence or pathogen distribution. We recommend future studies spanning full calendar years to assess seasonal patterns. | Lines 323-325 (Discussion - Limitations) |
| **R3-5** | Are there any differences in the populations served by the two health centres; are they urban or rural. How affluent are they? | We have added a description of the study sites and populations served. Both health centers serve peri-urban populations in Mwanza with similar socioeconomic characteristics (predominantly low to middle-income residents). This information provides important context for interpreting the findings. | Lines 95-100 (Methods - Study setting) |
| **R3-6** | Did the study adhere to the proposed inclusion criteria? Results suggest that criteria were not adhered to with participants that did not have the 2 required urinary symptoms included (please clarify!!). | We thank the reviewer for this important comment. The study fully adhered to the proposed inclusion criteria. All enrolled participants had at least two urinary symptoms, as required by the study protocol. The apparent confusion may arise from the way the symptoms were presented in the Results section, where each symptom was reported separately. Some participants had more than two symptoms, and therefore the frequencies of individual symptoms should not be interpreted to mean that participants with fewer than two symptoms were included. We have clarified this in the manuscript. | Lines 108-112 (Methods); Lines 180-185 (Results); Lines 310-315 (Discussion) |
| **R3-7** | It is indicated that specimens were collected into a clean container. Is that standard or should a sterile container be used? | We have clarified that “clean” refers to the clean-catch midstream technique using sterile containers, which is the standard method for urine collection for culture. The terminology has been corrected to “sterile container” to avoid confusion. | Line 115 (Methods) |
| **R3-8** | It is reported that urine dipstick and microscopy were done. Were results ever used in the study? | We have clarified that dipstick and microscopy were performed as part of routine clinical care but were not used as inclusion/exclusion criteria for the study, as culture remains the gold standard. We have added a statement about this and noted that future analyses may examine the diagnostic performance of these rapid tests compared to culture. | Lines 118-120 (Methods) |
| **R3-9** | It is indicated that colony counts of 1,000 colonies or more were considered significant bacteria? Were 1,000 actually counted? | We have clarified the methodology. The standard threshold is ≥10^5^ CFU/mL (100,000 colonies per mL), which we used. The text has been corrected to reflect this standard microbiological criterion. Colony counts were estimated using standard semi-quantitative culture techniques (calibrated loop method). | Lines 122-125 (Methods) |
| **R3-10** | It is indicated that standard biochemical tests were performed. The scope of biochemical testing tends to vary between labs. It would be appropriate to indicate the battery that was done for the study. | We have added a detailed description of the biochemical tests used for bacterial identification, including: catalase, coagulase, oxidase, indole, citrate, urease, and carbohydrate fermentation tests. We have also noted that MALDI-TOF was used for definitive identification when available. | Lines 125-130 (Methods) |
| **R3-11** | Enterococcus spp were not identified to species level which often helps distinguish between community and hospital acquired! | This is a valid point. We have added this as a limitation, noting that species-level identification of Enterococcus (E. faecalis vs E. faecium) would have provided additional epidemiological insight. We recommend this for future studies. | Lines 330-332 (Discussion - Limitations) |
| **R3-12** | How was ESBL screening performed! | We have added detailed methodology for ESBL detection, specifying that we used the CLSI-recommended combination disk diffusion method with cefotaxime and ceftazidime with and without clavulanic acid, with interpretation according to CLSI M100 guidelines. | Lines 135-138 (Methods) |
| **R3-13** | Did prior antibiotic use have any effect on bacteruria, types of organisms recovered (species/resistance)? | We thank the reviewer for this important question. In our study, only a small proportion of participants (15%) reported prior antibiotic use. Due to this limited number, we did not perform a sub-analysis to assess the effect of prior antibiotic use on bacteriuria, bacterial species distribution, or antimicrobial resistance patterns, as this would have resulted in insufficient statistical power to detect meaningful associations |  |
| **R3-14** | Was information collected on more remote prior use of antibiotics. AMR studies often look at antibiotic use during the past 90 days? What justifies the short duration? | We thank the reviewer for this important comment. In this study, information on prior antibiotic use was collected based on recent use as reported by participants, focusing on minimizing recall bias and improving the reliability of responses. While we acknowledge that assessing antibiotic exposure over a longer period (e.g., 90 days) is commonly used in antimicrobial resistance studies, the shorter recall period in our study was chosen to enhance accuracy of self-reported data in a primary healthcare setting. | Lines 110-112 Methods in data collection section |
| **R3-15** | Results: S. aureus is an unusual cause of uncomplicated UTI. Did these patients have any features of systemic involvement or upper UTI? Did they have any underlying morbidities? Was the identification rigorous enough to ensure that there was not misidentification of the S. aureus? What biochemical tests were performed? | We acknowledge that *Staphylococcus aureus* is an uncommon cause of uncomplicated urinary tract infections. In this study, participants were enrolled based on urinary symptoms at the primary healthcare level, and a detailed assessment of systemic involvement or underlying comorbidities was beyond the study scope. Identification of *S. aureus* was performed using standard microbiological methods, including Gram staining, catalase, and coagulase tests, making misidentification unlikely. We have clarified this in the manuscript and indicated that the finding should be interpreted with caution. | Lines 185-190 (Results); Lines 278-285 (Discussion - dedicated subsection on S. aureus findings) |
| **R3-16** | Table 3 and figure 2 give exactly the same data? Are they both relevant? | We agree that this is redundant. We have removed Figure 2 and retained Table 3, as the table format provides more precise numerical data that is more useful for reference. | Figure 2 removed; Table 3 retained |
| **R3-17** | Was there a basis for highlighting particular antibiotics here: e.g., Nitrofurantoin, which is often used for uncomplicated cystitis, cotrimoxazole, and ciprofloxacin, which have often previously been used! | Yes, these antibiotics were highlighted because they are recommended first-line or second-line agents in the Tanzanian Standard Treatment Guidelines for UTI. We have added this rationale to the methods section and referenced the national guidelines. | Lines 140-142 (Methods); Reference to Tanzania STG added |
| **R3-18** | Are the antimicrobials tested against the Pseudomonas and other non-fermentative Gram-negative rods the suitable ones? | As noted in response to Reviewer 2, we have corrected this to include only CLSI-recommended antibiotics for non-fermenters (ceftazidime, ciprofloxacin, gentamicin, amikacin, piperacillin-tazobactam, meropenem) and removed inappropriate agents. | Lines 215-220 (Results); Tables 4-6 revised |
| **R3-19** | Less than half of isolates resistant to a third generation cephalosporin were ESBL producing. Was any attempt made to find out the mechanisms of resistance? | We agree that resistance to third-generation cephalosporins may be mediated by mechanisms other than ESBL production. However, investigating specific resistance mechanisms was beyond the scope of this study, which focused on bacterial identification and antimicrobial susceptibility patterns. Notably, ESBL production is known to be the major mechanism of resistance among Gram-negative isolates in similar settings, although other mechanisms may also contribute |  |
| **R3-20** | Looks like most of the participants did not have symptoms localising to the urinary tract. Was the recruitment criteria followed as is proposed in the methods? | We have clarified that all participants had at least 2 urinary symptoms as per the inclusion criteria, but many presented with atypical symptom combinations. | Lines 180-185 (Results); Lines 310-315 (Discussion) |
| **R3-21** | This high proportion of S. aureus is rather curious and unusual. S. aureus rarely causes simple cystitis. It is much more often associated with UTIs that’s complicated (e.g., abscesses within the urinary tract, bacteremia). Is there anything to suggest that the patients fit such a profile? Are we sure that the isolates were S. aureus and not another species that gave an apparent positive coagulase? | Addressed in R3-15 above. | Lines 185-190 (Results); Lines 278-285 (Discussion - dedicated subsection on S. aureus findings) |
| **R3-22** | Discussion: Did most of your participants have symptoms localising to the urinary tract. How did it affect the study? | We have added discussion of this important finding. While all participants had urinary symptoms, the high frequency of atypical presentations may indicate: (1) Diagnostic challenges in primary care settings; (2) Possible inclusion of some patients without true UTI; (3) Need for improved clinical assessment tools. This is discussed as both a finding and a limitation. | Lines 310-315 (Discussion); Lines 325-328 (Limitations) |
| **R3-23** | This high proportion of S. aureus is rather curious and unusual. [Repeated comment - same as R3-21] | Addressed in R3-15 above. | Lines 278-285 (Discussion) |
| **R3-24** | Would be more meaningful to discuss the antimicrobial susceptibility profiles in the context of a normative document such as a clinical guideline. | Excellent suggestion. We have restructured the discussion to explicitly compare our findings to the Tanzanian Standard Treatment Guidelines and WHO recommendations for empirical UTI treatment, highlighting where current guidelines are supported or contradicted by our data. | Lines 289-301 (Discussion - restructured section) |
| **R3-25** | Piperacillin-tazobactam and amikacin are not included in the data, what’s the basis of their discussion here? | We have removed discussion of antibiotics not tested in our study, or clearly indicated when we are discussing them in the context of recommendations for future consideration based on literature, not our own data. | Lines 295-301 (Discussion - revised) |
| **R3-26** | Resistance to 3rd generation cephalosporins and ESBL are particularly important resistance types that deserve further discussion. Was the observed prevalence of resistance what was expected in the context of other existing data? | We have expanded the discussion of ESBL and cephalosporin resistance, comparing our findings (20.3% ESBL in E. coli) to previous studies in Tanzania and the region. We note that this represents an increase from earlier studies and discuss the public health implications. | Lines 292-298 (Discussion - expanded section) |
