# Supplementary material for: Multidrug resistance in bacteria causing community-acquired urinary tract infections among adult outpatients attending primary healthcare facilities in Mwanza, Tanzania
Source: PLOS Glob Public Health. 2026 Oct 8;6(10):e0005638. doi: 10.1371/journal.pgph.0005638 (PMC13649018; doi:10.1371/journal.pgph.0005638)
Supplement: Supplementary file 6 [file pgph.0005638.s009.docx]

**Multidrug resistance in bacteria causing community acquired urinary tract infections among adult outpatients attending primary healthcare facilities in Mwanza, Tanzania**

Eunice G. Emmanuel^1,2^, Ashura Khamis^1^, Elikana Michael^1^, Dorina Muhizi^1^, James Thomas^1^, Bernard Okamo^3^, Farida I. Mkassy^4^, Eveline T. Konje^4^, Vitus Silago^5^, Martha F. Mushi^5*^, Stephen E. Mshana^5^

1. Weil Bugando School of Medicine, Catholic University of Health and Allied Sciences, P.O Box 1464, Mwanza, Tanzania
2. Pharmacy Department, Igoma Health Centre, Ilemela, Mwanza Tanzania
3. Department of Biochemistry and Molecular Biology Catholic University of Health and Allied Sciences, P.O Box 1464, Mwanza, Tanzania
4. Department of Epidemiology, Biostatistics and Behavioural science, School of Public Health, Catholic University of Health and Allied Sciences, P.O Box 1464, Mwanza, Tanzania
5. Department of Microbiology and immunology, Weil Bugando School of Medicine, Catholic University of Health and Allied Sciences, P.O Box 1464, Mwanza, Tanzania

**Correspondence*:** Martha F. Mushi, Email [mushimartha@gmail.com](mailto:mushimartha@gmail.com)

**Abstract**

**Background:** Urinary tract infections (UTIs) remain a common clinical condition requiring antibiotic prescription among adult outpatients in low- and middle-income countries (LMICs). Data regarding the prevalence, patterns of bacteria, and their antibacterial susceptibility profile for community-acquired UTI at primary healthcare facilities are limited, particularly in resource-constrained settings where empirical treatment contributes to increased antimicrobial resistance (AMR) and associated morbidity.

**Methods:** A primary healthcare facility-based cross-sectional study was conducted from May to August 2024 at Igoma and Buzuruga Health Centres in Mwanza. Symptomatic adult patients, without recent hospitalization (within the past 30 days) or urinary catheterization, were enrolled. Clinical and demographic data were collected, followed by standard mid-stream urine for culture and disc diffusion susceptibility testing. Descriptive data analysis was performed using STATA version 15.

**Results:** A total of 1,005 adult patients with a median age of 32 [IQR: 23–49] years were recruited, of whom 727 (72.3%) were female. The majority (64.3%) reported a previous history of UTI (within six months), and the median symptom duration before presentation was 8 [5–15] days. Microbiological confirmation of UTI was found in 221 patients (22.0%, 95% CI 19.5%-24.7%). The most frequently isolated uropathogens were *Escherichia coli* (32.9%) and *Staphylococcus aureus* (24.7%). Notably, only 35.7% of culture-confirmed UTI patients presented with classic urinary symptoms (dysuria, frequency, urgency), highlighting clinical diagnostic challenges in this setting. *E. coli* isolates demonstrated alarmingly high resistance rates: ciprofloxacin (62.3%), trimethoprim-sulfamethoxazole (60.7%), amoxicillin-clavulanic acid (57.4%), tetracycline (55.7%), and ampicillin (93.4%). Critically, 50% of *E. coli* isolates exhibited multidrug resistance (MDR), and 20.3% showed extended-spectrum beta-lactamase (ESBL) phenotypes. Among *S. aureus* isolates, 64.6% were resistant to cefoxitin, indicating methicillin-resistant *S. aureus* (MRSA) strains.

**Conclusion and recommendation:** This study revealed a significant burden of community-acquired urinary tract infections (CA-UTIs) among adult outpatients at primary healthcare facilities, with an overall prevalence of 22%. *E. coli* and *S. aureus* were the most frequently isolated pathogens, with alarmingly high levels of antimicrobial resistance, particularly multidrug resistance in *E. coli*. The high prevalence of atypical clinical presentations and the failure of commonly used first-line antibiotics underscore the urgent need for improved diagnostic capacity, strengthened antibiotic stewardship programs, and updated treatment guidelines tailored to local resistance patterns to guide effective management of community-acquired UTIs and control AMR development. The high resistance to ciprofloxacin, trimethoprim-sulfamethoxazole, and amoxicillin-clavulanic acid indicates reduced effectiveness of commonly prescribed empirical therapies in primary healthcare settings.

**Key words:** Community-acquired UTI; Antimicrobial resistance; Escherichia coli; Multidrug resistance; Uropathogens; Outpatients; Tanzania

**Highlight:**

- A total of 1,005 symptomatic adult patients at primary healthcare facilities were assessed for community-acquired urinary tract infections (CA-UTIs).
- CA-UTIs were identified in 22% of cases, with *Escherichia coli* and *Staphylococcus aureus* as the most common pathogens.
- *E. coli* demonstrated a notably high rate of multidrug resistance, with about 50% of isolates resistant to multiple antibiotic classes.
- Over 70% of Gram-negative bacterial isolates were resistant to at least three classes of antibiotics, presenting major treatment difficulties.
- These results highlight the urgent need for ongoing surveillance and updated empirical treatment policies for UTIs in outpatient clinics, especially in resource-limited settings.

**Background**

Urinary tract infections (UTIs) are a prevalent health concern among patients visiting outpatient clinics in low- and middle-income countries (LMICs). In these regions, UTIs significantly contribute to morbidity, elevated treatment expenses, and mortality, especially among those seeking care at primary health care facilities [1, 2]. Limited diagnostic resources coupled with insufficient microbiological evidence often compromise effective patient management. However, UTI remains one of the most common reasons for antibiotic prescription and the most frequent reason for hospital attendance, surpassed only by malaria [3, 4]. In Tanzania, the prevalence of UTI is reported to be different among populations, ranging from 11-27.4% [5-9].

Enterobacterales such as *Escherichia coli* and *Klebsiella pneumoniae* are the leading causes of urinary tract infections (UTIs) in both community and healthcare settings[1, 9]. Many of these Gram-negative isolates produce extended-spectrum beta-lactamases (ESBLs), a major mechanism of antimicrobial resistance[10, 11]. Among Gram-positive bacteria, *Staphylococcus aureus* is commonly reported as a leading uropathogens[12]. In Tanzania, previous studies have shown high rates of multidrug resistance among UTI pathogens, ranging from 22.4%–60.3% in Gram-positive bacteria and 45.4%–52.2% in Gram-negative bacteria[4, 9, 13, 14].

Several factors increase the risk of community-acquired urinary tract infections (CA-UTIs). Women, older adults, and pregnant women are particularly vulnerable due to anatomical, physiological, and hormonal factors[15]. Behavioural factors such as poor hygiene, frequent sexual activity, and the use of spermicides or diaphragms also increase susceptibility. Medical conditions including diabetes mellitus, urinary tract obstruction, kidney stones, congenital abnormalities, and enlarged prostate further predispose individuals to infection by promoting bacterial growth and impairing urine flow. In addition, antibiotic use and catheterization can disrupt normal flora and facilitate bacterial colonization [4]. Since antimicrobial resistance patterns vary across regions and over time, local surveillance is important for guiding appropriate empirical treatment.

Previous studies in Tanzania have mainly focused on tertiary hospitals or specific groups, such as pregnant women, children, and people living with HIV [4-7, 9, 13, 14], leaving limited evidence on community-acquired UTIs among general adult outpatients attending primary healthcare facilities. In addition, Tanzania’s standard treatment guidelines recommend empirical treatment with ciprofloxacin, amoxicillin-clavulanic acid, and trimethoprim-sulfamethoxazole in lower health facility[16], which are also readily available over the counter. Understanding the prevalence, causative organisms, and resistance patterns in this setting is essential for informing targeted interventions, updating treatment guidelines, and strengthening antimicrobial stewardship programs.

This study aimed to determine the prevalence of CA-UTIs, identify the causative bacterial pathogens, and characterize their antimicrobial susceptibility profiles among adult outpatients attending primary healthcare facilities in Mwanza, Tanzania. By focusing on the primary care setting where most patients first present, this research provides critical insights into the current state of AMR in community settings and informs public health strategies to combat the rising threat of multidrug-resistant infections.

**Methodology**

**Study design and settings:** This prospective cross-sectional study was conducted at two primary healthcare facilities, Igoma and Buzuruga Health Centres in Mwanza, Tanzania, from 15th May to 30th August 2024. The study was implemented using predefined objectives, standardized enrolment criteria, and systematic microbiological procedures. Buzuruga Health Centre serves approximately 2,266 patients per month, while Igoma Health Centre attends about 1,500 outpatients monthly.

**Sample size and study population:** The study population included adult outpatients (≥18 years) presenting with symptoms suggestive of urinary tract infection (UTI). In accordance with established clinical guidelines [17] and the Tanzanian Standard Treatment Guidelines, participants were enrolled after clinical assessment by outpatient clinicians and were required to present with at least two suggestive symptoms, including dysuria, urinary frequency, urinary urgency, suprapubic pain, or lower abdominal pain.

The minimum sample size was obtained by the Kish-Leslie formula [18] using a 24.7% prevalence of CA-UTIs previously reported by Silago et *al*.[9]. To account for potential non-response and ensure adequate power for subgroup analyses, the sample was increased, resulting in a target enrollment of 1,005 participants. Pregnant women, patients with a history of admission within 30 days on the day of sample collection, and those with an indwelling urinary catheter at the time of study were excluded.

**Data and sample collections:** Demographic and clinical data were collected using Epicollect5, a mobile data-gathering platform developed by the CGPS Team of Oxford BDI and publicly available at <https://five.epicollect.net>. Information on prior antibiotic use was collected based on recent self-reported use to minimize recall bias and improve data reliability. About 3-5 ml of clean catch midstream urine was collected by the patients after receiving instructions from the research assistant, using a sterile, wide-mouthed, labelled container. The urine sample was transported to the microbiology research laboratory of CUHAS for processing within 2 hours of collection. For women, urine samples were tested for HCG to exclude pregnancy[19]. Urine was divided to two portions for microscopy/dipstick analysis and culture. All laboratory data were documented on the laboratory logbook.

**Urine Culture:** Urine samples were inoculated onto agar media (Blood Agar, MacConkey Agar and CLED) and incubated at 37^o^c for 18-24 hours. Growth estimate of ≥1000 colonies from 10µl inoculating loop for both men and women was considered as significant bacteriuria and thus UTI [20]. In-house-prepared conventional biochemical identification tests including: catalase, coagulase, oxidase, indole, citrate, urease, and sugar fermentation tests were used for the preliminary identification of bacterial isolates to their possible species levels as previously described [21]. Gram-positive and Gram-negative bacteria were identified using morphological and biochemical tests as previously described [22]. Unidentified isolates were further analysed using Matrix-Assisted Laser Desorption Ionization–Time of Flight (MALDI-TOF) mass spectrometry at the National Public Health Laboratory (NPHL)[23]. Antimicrobial susceptibility testing was performed as per CLSI guidelines [24].

Screening for extended-spectrum beta-lactamase (ESBL) production among Enterobacterales isolates was initially performed using ceftriaxone (30 µg) and ceftazidime (30 µg) discs during routine antimicrobial susceptibility testing. Isolates showing reduced susceptibility to either antibiotic were subjected to ESBL confirmation using the combination disc method according to CLSI guidelines [24].Confirmation of ESBL production was defined by an increase of ≥5 mm in the inhibition zone diameter for ceftazidime-clavulanic acid compared to ceftazidime alone.

For Gram-positive isolates, erythromycin and clindamycin discs were additionally used to screen for inducible macrolide-lincosamide-streptogramin B (iMLSB) resistance using the D-test according to CLSI guidelines [24]. Isolates showing resistance to erythromycin and susceptibility to clindamycin were subjected to the D-test by placing erythromycin and clindamycin discs 15–20 mm apart on Mueller-Hinton agar. Flattening of the clindamycin inhibition zone adjacent to the erythromycin disc (D-shaped zone) was interpreted as inducible clindamycin resistance. Erythromycin results were used only for D-test screening purposes and were not included in routine urinary antimicrobial susceptibility interpretation or multidrug resistance analysis.

**Quality Control:** Quality assurance measures encompassed monitoring reagent storage, equipment functionality, media sterility, and preparation of standardized bacterial suspensions. All stains and reagents were systematically labelled, dated, and stored under appropriate conditions. Refrigerator and incubator temperatures were recorded and tracked daily to ensure compliance with operational standards. Culture media were prepared in strict accordance with manufacturer instructions and evaluated for both performance and sterility. To maintain consistent inoculum density for susceptibility testing, a 0.5 McFarland standard was utilized; standard reference strains like *S. aureus* (ATCC 25923), *MRSA* (ATCC 29213), *E. coli* (ATCC 25922), ESBL-producing *Klebsiella pneumoniae* (ATCC 700603), and *Pseudomonas aeruginosa* (ATCC 27853) were employed as controls. Data cleaning and coding were conducted using STATA version 15, with records containing missing values excluded from analysis.

**Data Analysis:** Data were analysed using STATA software version 15 in line with the study's objectives. Descriptive statistics were used to summarize data, Continuous variables were summarized as medians with interquartile ranges (IQR), while categorical variables were summarized as frequencies and proportions. Comparisons between categorical variables were performed using Chi-square test or Fisher’s exact test where appropriate. A p-value of <0.05 was considered statistically significant.

**Ethical Considerations**: Ethical clearance was obtained from the Joint CUHAS/BMC Research Ethics and Review Committee (CREC) with certificate number CREC No 763/2024. All participants were requested to consent by signing written informed consent form. Privacy and confidentiality were maintained throughout the study. The urine culture results were timely shared to the manage clinicians to guide the management.

**Results**

The study recruited a total of 1005 adult, patients with a clinical diagnosis of UTI. In general, majority of recruited patients were non-pregnant women 727(72.3%), about two third were married 610 (60.7%) and their median age was 32[23-49] years, as detailed in table1.

Regarding clinical history, 646 participants (64.3%) reported a previous episode of UTI within the past six months, and 129 (12.8%) had used antibiotics within the 14 days preceding enrollment. The median duration of symptoms before seeking care was 8 days (IQR: 5-15 days). Notably, only 359 participants (35.7%) presented with classic urinary tract symptoms (dysuria, frequency, and urgency), while the majority presented with non-specific symptoms such as lower abdominal pain, fever, or lower back pain, highlighting the clinical diagnostic challenges in this setting. Patients recruited from Igoma health centre reported late to the health facility than those from Buzuruga health centre (14[7-28] vs. 7[3-14] days), P=0.001. as indicated in table 1.

**Table 1: Demographic and clinical characteristics of the studied population**

| **Variable** | | **Buzuruga HC (566)**  **n(%)** | **Igoma HC (439)**  **n(%)** | **Total (1005)** |
| --- | --- | --- | --- | --- |
| Gender | Male | 138(24.4) | 140(31.9) | 278(27.7) |
|  | Female | 428(75.6) | 299(68.1) | 727(72.3) |
| Age | Median [IQR] | 32[23-50] | 30[22-49] | 32[23-49] |
| Education | No formal education | 37(6.5) | 65(14.8) | 102(10.2) |
|  | Primary | 243(42.9) | 216(49.2) | 459(45.7) |
|  | Secondary | 220(38.9) | 123(28.0) | 343(34.1) |
|  | University | 66(11.7) | 35(7.9) | 101(10.1) |
| Marital status | Married | 367(64.8) | 243(55.4) | 610(60.7) |
|  | Single | 199(35.2) | 196(44.6) | 395(39.3) |
| History of UTI | Yes | 382(67.5) | 264(60.1) | 646(64.3) |
|  | No | 184(32.5) | 175(39.9) | 359(35.7) |
| Dysuria | Yes | 137(24.2) | 117(26.6) | 254(25.3) |
|  | No | 429(75.8) | 322(73.4) | 751(74.7) |
| Urgency | Yes | 54(9.5) | 16(3.6) | 70(6.9) |
|  | No | 512(90.5) | 423(96.4) | 935(93.0) |
| Frequency | Yes | 66(11.7) | 40(9.1) | 106(10.6) |
|  | No | 500(88.3) | 399(90.9) | 899(89.4) |
| Nocturia | Yes | 20(3.5) | 22(5.0) | 42(4.2) |
|  | No | 546(96.5) | 417(94.9) | 963(95.8) |
| Cloudy urine | Yes | 58(10.3) | 31(7.1) | 89(8.9) |
|  | No | 508(89.7) | 408(92.9) | 916(91.1) |
| Smelling urine | Yes | 40(7.1) | 14(3.2) | 54(5.4) |
|  | No | 526(92.9) | 425(96.8) | 951(94.6) |
| Lower abdominal pain | Yes | 275(48.6) | 237(53.9) | 512(50.9) |
|  | No | 291(51.4) | 202(46.0) | 493(49.1) |
| Lower back pain | Yes | 161(28.5) | 212(48.3) | 373(37.1) |
|  | No | 405(71.5) | 227(51.7) | 632(62.9) |
| Fever | Yes | 377(66.6) | 211(48.1) | 588(58.5) |
|  | No | 189(33.4) | 228(51.9) | 417(41.5) |
| Duration of symptoms (days) | | 7[3-14] | 14[7-28] | 8[5-15] |
| Presence of co-morbidity | Yes | 96(16.9) | 26(5.9) | 122(12.1) |
|  | No | 470(83.0) | 413(94.1) | 883(87.9) |
| Current antibiotic use | Yes | 89(15.7) | 40(9.1) | 129(12.8) |
|  | No | 477(84.3) | 399(90.9) | 876(87.2) |
| Co-morbidity | High blood Pressure | 26(27.1%) | 12(46.2%) | 38(31.1%) |
|  | Diabetic Mellitus | 19(19.8%) | 6(23.1%) | 25(20.5%) |
|  | Chronic Back Pain | 19(19.8%) | 4(15.4%) | 23(18.9%) |
|  | Peptic Ulcer Diseases | 23(23.9%) | 0(0.0%) | 23(18.9%) |
|  | Others | 9(9.4%) | 4(15.4%) | 13(10.7%) |

Others included Benign prostatic hyperplasia 2; living with HIV 6; chronic leg pain 1; ovarian cyst 4

Among the recruited patients with other comorbidities (n=122), high blood pressure was the commonest, 38 (31.1%). A total of seven patients had more than one chronic condition (co-morbidity) such as diabetic mellitus and hypertension (six patients), and one patient had both hypertension and peptic ulcer disease making a total count of 122.

**Prevalence of community acquired UTI**

Of the 1005 recruited patients with clinical presentation of urinary tract infections, 221(22%), 95% CI 19.4%-24.6% were microbiologically confirmed. Igoma health centre had 22.8%, (n=100/439), 95% CI 18.9%-27% patients with microbiologically confirmed UTI while Buzuruga health centre had 21.4%, (n=121/566), 95% CI 18%-25% patients.

**Proportions of bacterial species causing CA-UTIs**

Of the 221 microbiologically confirmed cases, 194 were due to bacterial isolates, while 27 showed significant growth of Candida spp. The majority of bacteria isolated were Gram-negative (59.8%; n = 116). Overall, the most frequently detected bacterial species were E. coli (32.9%; n = 64) and S. aureus (24.7%; n = 48). A similar pattern of predominant bacterial pathogens was observed at both Buzuruga Health Centre and Igoma Health Centre, however, the third and fourth most common isolates differed slightly between the two sites. At Buzuruga HC, Enterococcus spp. (10.5%; n = 11) and Acinetobacter spp. (9.5%; n = 10) were the commonest third and fourth pathogen, respectively while at Igoma Acinetobacter spp. (10.1%; n = 9) and K. pneumoniae complex (8.9%; n = 8) were common. Notably, all C. freundii strains were exclusively isolated at Buzuruga HC (Table 2).

**Table 2: The proportions of bacterial species causing community-acquired urinary tract infections**

| **Isolate** | **All (n=194)** | **Buzuruga HC (N=105)** | **Igoma HC (N=89)** |
| --- | --- | --- | --- |
|  | **n(%)** | **n(%)** | **n(%)** |
| *E. coli* | 64 (32.9) | 33 (31.4) | 31 (34.8) |
| *S. aureus* | 48 (24.7) | 25 (23.8) | 23 (25.8) |
| *Acinetobacter* spp. | 19 (9.8) | 10 (9.5) | 9 (10.1) |
| *Enterococcus* spp. | 16 (8.2) | 11 (10.5) | 5 (5.6) |
| *K. pneumoniae* complex | 12 (6.2) | 4 (3.8) | 8 (8.9) |
| *S. pyogenes* | 10 (5.2) | 5 (4.8) | 5(5.6) |
| *P. aeruginosa* | 7 (3.6) | 2 (1.9) | 5 (5.6) |
| *C. freundii* | 5 (2.6) | 5 (4.8) | 0 (0.0) |
| Others* | 13 (6.7) | 10 (9.5) | 3 (3.4) |

***Others: n=13:** *Morganella morganii* (n=3), *Enterobacter cloacae* complex (n=2), *Citrobacter koseri* (n=1), *Klebsiella oxytoca* (n=1), *Pseudomonas fluorescens* (n=1), *Proteus mirabilis* (n=1), *Pseudoglutamicibacter cumminsii* (n=1), *Streptococcus agalactiae* (n=1), *Staphylococcus epidermidis* (n=1), and *Staphylococcus haemolyticus* (n=1).

**Buzuruga HC (n=10):** *Morganella morganii* (n=3), *Enterobacter cloacae* complex (n=1), *Citrobacter koseri* (n=1), *Klebsiella oxytoca* (n=1), *Pseudomonas fluorescens* (n=1), *Pseudoglutamicibacter cumminsii* (n=1), *Staphylococcus epidermidis* (n=1), and *Staphylococcus haemolyticus* (n=1).

**Igoma HC (n=3):** *Enterobacter cloacae* complex (n=1), *Proteus mirabilis* (n=1), and *Streptococcus agalactiae* (n=1).

**Percentage resistance of bacterial pathogens causing CA-UTIs**

*Escherichia coli* demonstrated the highest levels of antimicrobial resistance (≥50%) to multiple antibiotics, including ciprofloxacin, amoxicillin-clavulanic acid, trimethoprim-sulfamethoxazole, tetracycline, and ampicillin. Among *E. coli* isolates, 45.3% were resistant to ceftriaxone, while 20.3% demonstrated positive phenotypes of extended-spectrum beta-lactamase (ESBL) production. Other members of the Enterobacterales group showed similarly high resistance rates to amoxicillin-clavulanic acid and ampicillin, with 12.0% detected with ESBL phenotypes. The non-fermentative Gram-negative bacteria, specifically *Acinetobacter* spp. and *Pseudomonas* spp., exhibited the highest resistance to amoxicillin-clavulanic acid, trimethoprim-sulfamethoxazole, ceftriaxone, and ampicillin (Table 3).

On the other hand, *S. aureus* demonstrated markedly high resistance rates (≥ 50%) to ciprofloxacin, tetracycline, trimethoprim-sulfamethoxazole, ampicillin, and erythromycin. Moreover, 64.6% of *S. aureus* strains were resistant to cefoxitin, hence methicillin-resistant *S. aureus* (MRSA). Similarly, other Gram-negative bacteria exhibited peak resistance (≥ 50%) to ampicillin, tetracycline, trimethoprim-sulfamethoxazole, ciprofloxacin, and erythromycin (Table 3).

**Table 3: Percentage resistance of bacteria causing community-acquired urinary tract infection**

|  | **Gram negative bacteria** | | | | | |
| --- | --- | --- | --- | --- | --- | --- |
| **Antibiotic agents tested** | ***E. coli* (N=64)** | | **Other Enterobacterales (N=25)** | | **Non-fermentative Gram-negative bacteria (N=27)** | |
|  | **% Resistance** | **% Sensitive** | **% Resistance** | **% Sensitive** | **% Resistance** | **% Sensitive** |
| AMP | 58 (90.6) | 6 (9.4) | 25 (100) | 0 (0.0) | 5 (100) | 0 (0.0) |
| TET | 47 (73.4) | 17 (26.6) | 10 (40.0) | 15 (60.0) | 11 (47.8) | 12 (52.2) |
| SXT | 46 (73.0) | 17 (27.0) | 10 (40.0) | 15 (60.0) | NA | |
| CIP | 34 (53.1) | 30 (46.9) | 8 (32.0) | 17 (68.0) | 9 (33.3) | 18 (66.7) |
| NIT | 26 (40.6) | 38 (59.4) | 14 (56.0) | 11 (44.0) | 25 (92.6) | 2 (7.4) |
| AMC | 41 (64.1) | 23 (35.9) | 13 (52.0) | 12 (48.0) | NA | |
| CRO | 29 (45.3) | 35 (54.7) | 11 (44.0) | 14 (56.00) | NA | |
| GEN | 29 (45.3) | 35 (54.7) | 6 (25.0) | 18 (75.0) | 4 (15.4) | 22 (86.4) |
| MEM | 5 (7.9) | 58 (92.1) | 4 (16.0) | 21 (84.0) | 0 (0.0) | 26 (100) |
| ESBL strains | 13 (20.3) | | 3 (12.0) | | NA | |
| ***Antibiotic agents tested*** | ***Gram positive bacteria*** | | | | | |
|  | ***S. aureus* (N=48)** | | **Other Gram-positive bacteria (N=29)** | | | |
|  | **% Resistance** | **% Sensitive** | **% Resistance** | | **% Sensitive** | |
| ER | NA | | 28 (96.6) | | 1 (3.4) | |
| CIP | 24 (50.0) | 24 (50.0) | 25 (86.2) | | 4 (13.8) | |
| SXT | 31 (64.6) | 17 (35.4) | 24 (82.8) | | 5 (17.2) | |
| TET | 28 (58.4) | 20 (41.6) | 22 (75.9) | | 7 (24.1) | |
| PEN | 38 (79.2) | 10 (20.8) | 15 (51.7) | | 14 (48.3) | |
| CD | 29 (31.3) | 33 (68.7) | 14 (48.3) | | 15 (51.7) | |
| GEN | 11 (22.9) | 37 (77.1) | 11 (37.9) | | 18 (62.1) | |
| NIT | 12 (25.0) | 36 (75.0) | 11 (37.9) | | 18 (62.1) | |
| FOX | 31 (64.6) | 17 (35.4) | NT | | NT | |

Key: AMP=ampicillin, TET=tetracycline, SXT=trimethoprim-sulfamethoxazole, CIP=ciprofloxacin, NIT=nitrofurantoin, AMC=amoxicillin-clavulanic acid, CRO=ceftriaxone, GEN=gentamicin, NA=not applicable, and MEM=meropenem; ER=erythromycin; CIP=ciprofloxacin; SXT=trimethoprim-sulfamethoxazole; TET=tetracycline; PEN=penicillin; CD=clindamycin; GEN=gentamicin; NIT-nitrofurantoin; and FOX=cefoxitin.

**Proportions of multidrug resistance and resistance patterns of Gram-negative bacteria causing CA-UTIs**

Overall, the majority of Gram-negative bacteria (73.3%; 85/116) exhibited resistance to at least three classes of antimicrobials, with the number of classes ranging from three to six. By healthcare facility, the difference in proportions of MDR in Gram-negative bacteria isolated from patients attending at Buzuruga HC (70.5%; 43/61) and Igoma HC (76.4%; 42/55) was not statistically significant (p=0.475). *E. coli* showed the highest proportion of multidrug resistance (MDR), with 82.8% (53/64) of isolates classified as MDR, including 15.1% (n = 8) that were resistant to six antimicrobial classes. The common resistance patterns in *E. coli* were AMP-TET-SXT-CIP-NIT-AMC-CRO-CAZ-FEP-GEN-AK-TZP (n=3), AMP-TET-SXT-CIP-NIT-AMC-CRO-CAZ-FEP-GEN-TZP (n=2), and AMP-TET-SXT-CIP-AMC-CRO-CAZ-FEP-AK-TZP (n=2). Among other Enterobacterales, 44.0% (11/25) were MDR, with 27.3% (n = 3) also resistant to six classes. Similarly, 77.8% (22/27) of non-fermentative Gram-negative bacteria displayed MDR, with 9.1% (n = 2) resistant to six antimicrobial classes. The common resistance patterns in non-fermentative Gram-negative bacteria were SXT-NIT-CRO-CAZ-FEP (n=2) and TET-SXT-NIT-CRO-FEP-TZP (n=2) (Table 3). Detailed antimicrobial resistance patterns are provided in Supplementary File 1.

Among GPB, a substantial proportion of *S. aureus* isolates (83.3%; 40/48) were classified as MDR. Notably, 15.0% (6/40) of these MDR *S. aureus* strains exhibited resistance to as many as seven antibiotics. MDR phenotypes were significantly more prevalent among *S. aureus* isolates from patients at Igoma Health Centre compared to those from Buzuruga HC (95.6% vs. 72.0%; p = 0.019). The most frequently observed resistance patterns in *S. aureus* included CD-ER-AMP-SXT-TET-CIP-FOX (n=3), -AMP-SXT-TET-CIP-FOX (n=3), and -AMP-SXT-TET-NIT-CIP-FOX-GEN (n=3). For other GPB, all isolates (100%; 29/29) were MDR. The most common resistance profiles among these included CD--AMP-SXT-TET-CIP (n=2), CD--NIT-CIP (n=2), and -AMP-SXT-TET-NIT-CIP (n=2). Moreover, 24.1% (7/29) of these MDR GPB strains demonstrated resistance to six antimicrobial agents (Table 4). Detailed antimicrobial resistance patterns are shown on Supplementary file 2.

**Table 4: Distribution of multidrug resistance and number of resistant antibiotic classes among bacterial isolates causing urinary tract infections in primary healthcare facilities**

| **Variable** | **Category** | ***E. coli* (N=64)** | **Other Enterobacterales (N=25)** | **Non-fermentative GNB (N=27)** | |  |
| --- | --- | --- | --- | --- | --- | --- |
|  |  | **n(%)** | **n(%)** | **n(%)** | |  |
| Multidrug-resistant (MDR) strain | Yes | 53 (82.8) | 11 (44.0) | 21 (77.8) | |  |
|  | No | 11 (17.2) | 14 (56.0) | 6 (22.2) | |  |
| The number of antibiotic classes to which the bacteria resist | 3 | 10 (18.9) | 2 (18.2) | 10 (45.4) | |  |
|  | 4 | 13 (24.5) | 4 (36.4) | 6 (27.3) | |  |
|  | 5 | 22 (41.5) | 2 (18.2) | 3 (13.6) | |  |
|  | 6 | 8 (15.1) | 3 (27.3) | 2 (9.1) | |  |
| Health Centre for isolation of MDR strains | Buzuruga HC (N=43) | 28 (65.1) | 9 (20.9) | 6 (13.9) | |  |
|  | Igoma HC (N=42) | 25 (59.5) | 12 (28.6) | 5 (11.9) | |  |
| **Variable** | **Category** | ***S. aureus* (N=48)** | | | **Other Gram-positive bacteria (N=29)** | |
|  |  | **n(%)** | | | **n(%)** | |
| Multidrug-resistant (MDR) strain | Yes | 40 (83.3) | | | 29 (100) | |
|  | No | 8 (16.7) | | | 0 (0.0) | |
| The number of antibiotic classes to which the bacteria resist | 3 | 9 (22.5) | | | 6 (20.7) | |
|  | 4 | 11 (27.5) | | | 8 (27.6) | |
|  | 5 | 12 (30.0) | | | 8 (27.6) | |
|  | 6 | 2 (5.0) | | | 7 (24.1) | |
|  | 7 | 6 (15.0) | | | 0 (0.0) | |
| Health centre for isolation of MDR strains | Buzuruga HC | 18/25 (72.0) | | | 18/18 (100) | |
|  | Igoma HC | 22/23 (95.6) | | | 11/11 (100) | |

**Discussion**

This study provides important insights into the bacterial causes and antimicrobial resistance patterns of community-acquired urinary tract infections among patients attending primary healthcare facilities in Mwanza, Tanzania. The findings demonstrate that *Escherichia coli* remains the predominant uropathogen, with a substantial proportion of isolates exhibiting multidrug resistance. Notably, high resistance was observed to several commonly used antibiotics, raising concerns about the effectiveness of current empirical treatment practices in primary healthcare settings. Although only a small proportion of participants reported prior antibiotic use, the observed resistance patterns suggest ongoing antimicrobial pressure within the community. Together, these findings highlight the growing public health challenge of antimicrobial resistance in community-acquired infections and underscore the need for strengthened surveillance, improved diagnostic capacity, and evidence-based antimicrobial stewardship to guide appropriate treatment.

The high prevalence of multidrug-resistant uropathogens observed in this study likely reflects multiple drivers operating at the community and healthcare levels. In many low- and middle-income countries, including Tanzania, antibiotics are frequently used empirically for urinary tract infections due to limited access to microbiological diagnostic services at primary healthcare facilities [25, 26]. In addition, inappropriate antibiotic use, including self-medication, incomplete treatment courses, and over-the-counter access to antimicrobials, has been reported as a major contributor to the emergence and spread of antimicrobial resistance in community-acquired infections[27, 28]. These factors create selective pressure that favours the survival and transmission of resistant bacterial strains. Addressing these drivers requires strengthening antimicrobial stewardship, improving access to diagnostic services to support targeted therapy, and enhancing surveillance of antimicrobial resistance to inform local treatment guidelines and public health interventions[29].

The antimicrobial resistance patterns observed in this study have important implications for empirical treatment of community-acquired urinary tract infections (CAUTIs) in primary healthcare settings. In our cohort, *Escherichia coli* was the predominant uropathogen, and a large proportion of isolates exhibited multidrug resistance, with high resistance observed to commonly prescribed antibiotics including amoxicillin-clavulanic acid and ciprofloxacin. These findings suggest that some antibiotics frequently used for empirical management of urinary tract infections may have reduced effectiveness in this setting. Similar resistance patterns have been reported in Tanzania and other parts of East Africa, where increasing resistance among uropathogens has been associated with widespread empirical antibiotic use and limited diagnostic capacity[9, 25, 26]. Clinicians managing suspected urinary tract infections at primary healthcare facilities should therefore consider local antimicrobial susceptibility patterns when selecting empirical therapy. Strengthening routine surveillance of antimicrobial resistance and integrating these data into national and facility-level treatment guidelines are essential to ensure that empirical treatment remains effective in the context of rising antimicrobial resistance [30-32].

The prevalence of community-acquired, microbiologically confirmed UTI in the present study was 22%, which falls within the previously reported national range of 11–23% from diverse populations in Tanzania [5-7]. However, this prevalence is slightly lower than the 27% reported by Silago et al. in the same setting three years earlier [9]. This discrepancy may be explained by differences in study populations; the earlier study included pregnant women a group with a physiologically higher susceptibility to UTI while the current study excluded them.

From a public health perspective, a 22% prevalence indicates that UTIs remain a substantial community health burden, contributing to frequent outpatient visits and antimicrobial prescriptions. This underscores the need for strengthened community-level infection prevention strategies, including improved personal hygiene practices, safe water access, and health education aimed at early care seeking and avoidance of self-medication[33]. The high proportion of atypical presentations highlights the need for improved diagnostic capacity at primary care facilities. Point-of-care urine culture systems, dipstick testing, and clinical decision support tools could improve diagnostic accuracy and reduce inappropriate antibiotic use. Importantly, most participants reported prior antibiotic use, a finding with significant implications. Such widespread community antibiotic exposure, often unregulated, can suppress bacterial growth and potentially lead to underestimation of true UTI prevalence while simultaneously exerting selection pressure that drives antimicrobial resistance (AMR).

In the current study, just like other studies, Gram-negative bacteria were predominant pathogens causing community-acquired UTI. The predominance of Gram-negative bacteria is partly contributed to the fact that these bacteria are normal microbiota of the gastrointestinal tract, and the proximity of the urinary and gastrointestinal tract in females makes it easy for the organisms to ascend and cause a urinary tract infection.

The predominance of Gram-negative bacteria, especially *E. coli*, is consistent with global and regional trends in community-acquired urinary tract infections (CA-UTIs), reflecting its well-established role as the primary uropathogen [9]. The notable presence of *S. aureus*, a Gram-positive organism, suggests potential shifts or local variations in CA-UTI causes, warranting further exploration. *Staphylococcus aureus* is an uncommon cause of uncomplicated urinary tract infections and is more frequently associated with complicated infections or hematogenous spread. In this study, participants were enrolled based on symptoms suggestive of urinary tract infection at primary healthcare level, and detailed evaluation for systemic involvement or underlying comorbidities was not performed. However, identification of *S. aureus* was conducted using standard microbiological methods, making misidentification unlikely. The clinical significance of *S. aureus* isolated from urine in this outpatient setting should therefore be interpreted cautiously,

Differences in less common isolates between the two health centres may be linked to local antimicrobial use, patient demographics, or environmental factors influencing bacterial populations. The exclusive detection of *C. freundii* at one site highlights potential micro-epidemiological differences, which could inform more targeted infection control and surveillance efforts. Although *Streptococcus pyogenes* is not a typical uropathogens, occasional cases have been reported, particularly in association with perineal colonization or ascending infection from the genital tract[34]. In this study, the organism was rarely isolated and its clinical significance should therefore be interpreted cautiously.

The high resistance observed, particularly among *E. coli* and non-fermentative Gram-negative bacteria, emphasizes the increasing challenge of treating CA-UTIs with commonly prescribed antibiotics [35]. These findings underscore the urgent need for ongoing surveillance, rational antimicrobial use, and updated treatment guidelines tailored to local resistance patterns to manage infections effectively and limit the spread of multidrug-resistant pathogens. The widespread multidrug resistance (MDR) observed in Gram-negative bacteria, with *E. coli* showing the highest levels, is particularly concerning, given its dominance as a uropathogen. Resistance to multiple antibiotic classes, including critical agents like piperacillin-tazobactam and amikacin, limits treatment options and reinforces the critical need for antimicrobial stewardship to address this growing threat.

The observed prevalence of ESBL-producing *Enterobacterales*, together with the high resistance to ceftriaxone among *E. coli* isolates, suggests reduced effectiveness of third-generation cephalosporins for empirical treatment of community-acquired urinary tract infections in this setting. These findings raise concern regarding the continued reliance on commonly prescribed beta-lactam antibiotics and highlight the importance of routine antimicrobial susceptibility surveillance to guide empirical therapy and support appropriate antibiotic selection at primary healthcare facilities.

The high MDR prevalence among *S. aureus* and other GPB in this study is consistent with findings by Silago et al. in Mwanza and Dar es Salaam, where similar resistance patterns to ciprofloxacin, tetracycline, and trimethoprim-sulfamethoxazole were observed [9]. Notably, the resistance profiles align with post-COVID-19 trends reported by the same group, showing persistent and widespread MDR uropathogens in Tanzania [36]. The significantly higher MDR burden at Igoma HC may reflect differences in local antimicrobial practices. These findings underscore the ongoing threat of resistant GPB pathogens and the urgent need for context-specific antimicrobial stewardship in outpatient settings. The resistance patterns observed in this study may therefore reflect ongoing community misuse or overuse of antibiotics previously reported [25, 26], reinforcing the urgency of antimicrobial stewardship interventions, regulation of non-prescription antibiotic sales, and public education on the risks of inappropriate antibiotic use.

**Study limitations**

The study was conducted over a relatively short period (4 months) during the dry season, and seasonal variations in UTI prevalence and etiology may exist. The study was limited to two primary healthcare facilities in an urban/peri-urban setting, and findings may not be generalizable to rural areas or other regions of Tanzania. The broad case definition for UTI symptoms may have included some patients without true UTI, though this reflects real-world primary care practice, where laboratory confirmation is often unavailable. Finally, information on antibiotic use was limited to the 14 days preceding enrollment, while longer-term antibiotic exposure (e.g., 90 days) may be more relevant for AMR risk assessment.

**Conclusion, public health implication and Recommendation**

**Conclusion:** This study revealed a significant burden of community-acquired urinary tract infections (CA-UTIs) among adult outpatients in Mwanza, Tanzania, with an overall prevalence of 22%. *Escherichia coli* and *Staphylococcus aureus* were the most frequently isolated pathogens, and alarmingly high levels of resistance, particularly multidrug resistance in *E. coli*, were observed. The late presentation of patients, limited recognition of classical UTI symptoms, and high recurrence rates further complicate clinical management.

**Public health implications and recommendations**

Following the findings of this study, strengthening routine local antimicrobial susceptibility surveillance is important to guide empirical treatment decisions for community-acquired urinary tract infections at primary healthcare facilities. The current empirical treatment guidelines may also require revision due to the high resistance observed to ciprofloxacin, trimethoprim-sulfamethoxazole, and amoxicillin-clavulanic acid among community uropathogens. In addition, improving access to urine dipstick testing, urine culture, and antimicrobial susceptibility testing could enhance diagnostic accuracy and reduce inappropriate antibiotic use in outpatient settings. Strengthening antimicrobial stewardship programs at primary healthcare facilities is also essential to promote rational antibiotic prescribing practices and support efforts to control antimicrobial resistance.

**References**

1.	Donkor ES, Horlortu PZ, Dayie NT, Obeng-Nkrumah N, Labi A-K. Community acquired urinary tract infections among adults in Accra, Ghana. Infection and Drug Resistance. 2019:2059-67.

2.	Murray CJ, Ikuta KS, Sharara F, Swetschinski L, Aguilar GR, Gray A, et al. Global burden of bacterial antimicrobial resistance in 2019: a systematic analysis. The lancet. 2022;399(10325):629-55.

3.	Phillip S, Mushi MF, Decano AG, Seni J, Mmbaga BT, Kumburu H, et al. Molecular characterizations of the coagulase-negative Staphylococci species causing urinary tract infection in Tanzania: a Laboratory-Based Cross-Sectional Study. Pathogens. 2023;12(2):180.

4.	Maldonado-Barragán A, Mshana SE, Keenan K, Ke X, Gillespie SH, Stelling J, et al. Predominance of multidrug-resistant bacteria causing urinary tract infections among symptomatic patients in East Africa: a call for action. JAC-antimicrobial resistance. 2024;6(1):dlae019.

5.	Msaki BP, Mshana SE, Hokororo A, Mazigo HD, Morona D. Prevalence and predictors of urinary tract infection and severe malaria among febrile children attending Makongoro health centre in Mwanza city, North-Western Tanzania. Archives of Public Health. 2012;70:1-8.

6.	Gidabayda JG, Philemon R, Abdallah MS, Saajan AM, Temu T, Kunjumu I, et al. Prevalence, aetiology, and antimicrobial susceptibility patterns of urinary tract infection amongst children admitted at Kilimanjaro Christian Medical Centre, Moshi, Tanzania. The East African Health Research Journal. 2017;1(1):53.

7.	Chaula T, Seni J, Ng’walida N, Kajura A, Mirambo MM, DeVinney R, et al. Urinary Tract Infections among HIV‐Positive Pregnant Women in Mwanza City, Tanzania, Are High and Predicted by Low CD4+ Count. International journal of microbiology. 2017;2017(1):4042686.

8.	Masoud SS, Majigo M, Silago V, Kunambi P, Nyawale H, Moremi N, et al. Utility of dipstick urinalysis in the diagnosis of urinary tract infections among outpatients in Mwanza and Dar es Salaam regions in Tanzania. Bulletin of the National Research Centre. 2024;48(1):6.

9.	Silago V, Moremi N, Mtebe M, Komba E, Masoud S, Mgaya FX, et al. Multidrug-resistant uropathogens causing community acquired urinary tract infections among patients attending health facilities in Mwanza and Dar es Salaam, Tanzania. Antibiotics. 2022;11(12):1718.

10.	Mitu FS, Al Maruf MA, Mahanty A, Huda AN, Khan SA, Rahman MM. Prevalence of extended spectrum beta-lactamase (ESBL) and AmpC beta-lactamase producing bacteria in urinary tract infection patients in Bangladesh. Malaysian journal of microbiology. 2019;15(3):204-12.

11.	Bedenić B, Meštrović T. Mechanisms of resistance in gram-negative urinary pathogens: From country-specific molecular insights to global clinical relevance. Diagnostics. 2021;11(5):800.

12.	Kabugo D, Kizito S, Ashok DD, Kiwanuka AG, Nabimba R, Namunana S, et al. Factors associated with community-acquired urinary tract infections among adults attending assessment centre, Mulago Hospital Uganda. African health sciences. 2016;16(4):1131-42.

13.	Mlugu EM, Mohamedi JA, Sangeda RZ, Mwambete KD. Prevalence of urinary tract infection and antimicrobial resistance patterns of uropathogens with biofilm forming capacity among outpatients in morogoro, Tanzania: a cross-sectional study. BMC infectious diseases. 2023;23(1):660.

14.	Ngowi BN, Sunguya B, Herman A, Chacha A, Maro E, Rugarabamu LF, et al. Prevalence of multidrug resistant UTI among people living with HIV in Northern Tanzania. Infection and drug resistance. 2021:1623-33.

15.	Konje ET, Kizenga O, Charco NJ, Kibwana UO, Shango N, Tarimo F, et al. Five-year cross-sectional study to determine the burden of Candida spp. infections of the urinary tract system among patients attending tertiary hospital in Northwestern Tanzania. BMJ open. 2023;13(12):e074833.

16.	MOH. Standard Treatment Guidelines and National Essential Medicines List. 2021;Tanzania Main Land Sixth Edition:234.

17.	Ministry of Health CD, Gender, Elderly and Children. Standard Treatment Guidelines and National Essential Medicines List for Tanzania Mainland. Dar es Salaam: Ministry of Health, Community Development, Gender, Elderly and Children. 2021;6th Edition.

18.	Kish L. Sampling organizations and groups of unequal sizes. American sociological review. 1965:564-72.

19.	Chard T. Pregnancy tests: a review. Human reproduction. 1992;7(5):701-10.

20.	Majigo MV, Mshana S, Komba E, Moremi N, Matee M. Protocol for surveillance of antimicrobial-resistant bacteria causing community-acquired urinary tract infections in low-income countries. Plos one. 2024;19(5):e0304388.

21.	D.A. P, W. G, L.C. D, S.H. G, M.J. W, E.W. K. Koneman’s Color Atlas and Textbook of Diagnostic Microbiology, . Philadelphia, PA, USA: Wolters Kluwer Health; 2017.

22.	Alghamdi A, Almajid M, Alalawi R, Alganame A, Alanazi S, Alghamdi G, et al. Evaluation of asymptomatic bacteruria management before and after antimicrobial stewardship program implementation: retrospective study. BMC Infectious Diseases. 2021;21:1-5.

23.	Carbonnelle E, Mesquita C, Bille E, Day N, Dauphin B, Beretti J-L, et al. MALDI-TOF mass spectrometry tools for bacterial identification in clinical microbiology laboratory. Clinical biochemistry. 2011;44(1):104-9.

24.	CLSI. CLSI. *Perfomance standards for Antimicrobial Susceptibility Testing. 33^rd^ ed.*CLSI Supplement M100. Wayne, PA: Clinical and Laboratory Standard Institute; 2023; 2023.

25.	Ndaki PM, Mushi MF, Mwanga JR, Konje ET, Mugassa S, Manyiri MW, et al. Non-prescribed antibiotic dispensing practices for symptoms of urinary tract infection in community pharmacies and accredited drug dispensing outlets in Tanzania: a simulated clients approach. BMC Primary Care. 2022;23(1):287.

26.	Ndaki PM, Mwanga JR, Mushi MF, Konje ET, Mwita SM, Mshana SE. Drivers of inappropriate use of antibiotics among community members in low-and middle-income countries: a systematic review of qualitative studies. BMC Public Health. 2025;25(1):705.

27.	Mendelson M, Matsoso MP. The World Health Organization global action plan for antimicrobial resistance. SAMJ: South African Medical Journal. 2015;105(5):325-.

28.	Organization WH. Antimicrobial stewardship programmes in health-care facilities in low-and middle-income countries: a WHO practical toolkit. 2019.

29.	Organization WH. Antimicrobial resistance: global report on surveillance: World Health Organization; 2014.

30.	Organization WH. Kenya national action plan on antimicrobial resistance: review of progress in the human health sector. 2022.

31.	Charani E, Mendelson M, Pallett SJ, Ahmad R, Mpundu M, Mbamalu O, et al. An analysis of existing national action plans for antimicrobial resistance—gaps and opportunities in strategies optimising antibiotic use in human populations. The Lancet Global Health. 2023;11(3):e466-e74.

32.	Organization WH. WHO AWaRe Classification of Antibiotics for Evaluation and Monitoring of Use. 2019. WHO; 2019.

33.	Green DL, Keenan K, Fredricks KJ, Huque SI, Mushi MF, Kansiime C, et al. The role of multidimensional poverty in antibiotic misuse: a mixed-methods study of self-medication and non-adherence in Kenya, Tanzania, and Uganda. The Lancet Global Health. 2023;11(1):e59-e68.

34.	Flores-Mireles AL, Walker JN, Caparon M, Hultgren SJ. Urinary tract infections: epidemiology, mechanisms of infection and treatment options. Nature reviews microbiology. 2015;13(5):269-84.

35.	MoHT. Standard Treatment Guidelines and National Essential Medicines List for Tanzania Mainland. 6th ed. Dodoma, Tanzania: Ministry of Health, Tanzania; 2021. p. 491-3.

36.	Silago V, Keenan K, Mushi MF, Kansiime C, Asiimwe B, Sunday B, et al. Patterns of antibiotic resistance in urinary tract infections before and during the COVID-19 pandemic in Uganda and Tanzania. JAC-Antimicrobial Resistance. 2025;7(2):dlaf038.

**Supplementary files**

S1: Detailed antimicrobial susceptibility data

S2: Raw dataset
