# Supplementary material for: Multidrug resistance in bacteria causing community-acquired urinary tract infections among adult outpatients attending primary healthcare facilities in Mwanza, Tanzania
Source: PLOS Glob Public Health. 2026 Oct 8;6(10):e0005638. doi: 10.1371/journal.pgph.0005638 (PMC13649018; doi:10.1371/journal.pgph.0005638)
Supplement: Supplementary file 7 [file pgph.0005638.s010.docx]

Response to reviewers comments

Comments sent separately/ not included in the manuscript with track changes

| S/no | Reviewer comment | Response to reviewer |
| --- | --- | --- |
|  | Reviewer one |  |
| 1. | General comment: The manuscript’s strength is local, recent data from primary care and not tertiary with a large sample size. This directly informs empirical treatment choices at primary care level which is valuable. | We thank the reviewer for the positive assessment and for recognizing the clinical relevance of the findings. |
|  | Title a. It is clear, descriptive including location and population |  |
| 2. | Abstract a. The abstract is concise, representing numbers such as 22% prevalence, 32.9% E.coli, 24.7% S. aureus. It also mentions atypical presentations – which is clinically relevant  b. However: i. Resistance rates are listed but without clinical interpretations | We thank the reviewer for this important comment. We have revised the abstract and discussion to explicitly interpret the resistance findings in the context of empirical treatment failure and antimicrobial stewardship. Abstract Results and Conclusion sections (Pages 2–3) |
|  | ii. Public health implications are vague – improved diagnostic capacity is very vague and mentioned there as an over generalization of the findings | We appreciate this observation. We revised the conclusion and recommendation section to include specific recommendations directly linked to study findings. |
| 3. | Introduction a. Correctly identifies the knowledge gap: most prior studies from tertiary hospital or specific population. Acknowledges that AMR patterns vary by geography and time b. However: i. It is still very long and over detailed | We thank the reviewer. The introduction has been shortened and focused on the local burden of CA-UTIs and AMR in Tanzania. |
|  | ii. There is no statement of what empirical guidelines currently recommend in Tanzania and why those may be failing – a clinician needs to know the current standard of care and why is it challenged | We appreciate this important suggestion. We have now incorporated Tanzanian Standard Treatment Guidelines and linked them to the observed resistance patterns. |
| 4. | Methods a. Clear sample size calculation with Kish-Lesile formula b. Appropriate exclusion criteria have been listed c. Quality control described and ethical approval and consent properly documented d. However; i. CSLI violation: • Erythromycin against urine S.aureus – not a CLSI recommendation • Amoxiclav, cotrimoxazole, ceftriaxone, ampicillin – tested against non-fermenters – not recommendation • Nitrofurantoin tested against Proteus and Pseudomonas – these are intrinsically resistant | We thank the reviewers for identifying these important methodological issues. The susceptibility analysis has now been revised according to CLSI recommendations, see table 3 and Results section (Pages 11–12). |
|  | ii. The prior antibiotic use recall period is too short – only 14 days? AMR studies typically use 30-90 days? | We thank the reviewer for this important methodological observation. We acknowledge that longer recall periods are commonly used in antimicrobial resistance studies. However, in the current study, a 14-day recall period was intentionally selected to minimize recall bias and improve the reliability of self-reported antibiotic exposure. In addition, recent antibiotic exposure within this shorter timeframe was considered more clinically relevant to urine culture results, as recent antibiotic use may suppress bacterial growth and potentially reduce culture positivity. |
|  | iii. No statistical analysis plan described | We thank the reviewer for identifying this omission. The statistical analysis section has now been expanded. |
| 5. | Results a. Explanation for: i. Table 3 and figure 2 relay identical data | We thank the reviewer for this important observation. We agree that the information presented in Figure 2 substantially overlapped with Table 3. To improve clarity and avoid redundancy, Figure 2 has now been removed from the revised manuscript, and the antimicrobial resistance data are presented only in Table 3. |
|  | ii. S-aureus finding over interpreted – 24.7% of isolates S-aureus is highly unusual for CA-UTI - addressing contamination vs true infection – without this, many of these may be skin flora contamination | We appreciate this important observation. The discussion has been revised to cautiously interpret S. aureus isolation and acknowledge possible contamination or colonization. |
|  | iii. Candida in 27 patients – mentioned but not explored. Candida UTI is rare and most likely colonization or contamination | We agree that candiduria may represent colonization, contamination, or true infection depending on the clinical context. In the current study, Candida isolates were reported as part of microbiologically confirmed urinary findings; however, detailed characterization of Candida isolates was beyond the scope of the present manuscript. Further laboratory work involving species-level identification and antifungal susceptibility testing of the Candida isolates is still ongoing and will be presented separately in a dedicated manuscript focusing on fungal urinary tract infections. |
|  | iv. No analysis of association between symptoms and microbiology results which would yield high impact | We thank the reviewer for this valuable suggestion. We agree that analysis of associations between clinical symptoms and microbiologically confirmed urinary tract infections would provide important clinical insights. However, this study was designed as a descriptive cross-sectional and was not specifically powered or structured to comprehensively evaluate predictors or factors associated with microbiologically confirmed UTI. |
| 6. | Discussion a. The discussion is appropriate. A few recommendations: i. The over interpretation of S.aureus | We thank the reviewer for this important observation. We have revised the discussion to avoid overinterpretation of the findings and to provide a more cautious interpretation while acknowledging that standard microbiological identification procedures were followed |
|  | ii. No discussion on the candida species | We appreciate this important comment. Candida isolates were reported as part of microbiologically confirmed urinary findings; however, detailed characterization of Candida isolates was beyond the scope of the current manuscript, which primarily focused on bacterial etiologies and antibacterial resistance patterns. |
|  | iii. Drivers of AMR are discussed generically – self-medication, OTC access without linking to this study’s data | We thank the reviewer for this important observation. We agree that some discussion on AMR drivers was overly generalized and not directly supported by our data. We have therefore revised the discussion to focus mainly on findings from this study, particularly the high prevalence of multidrug-resistant organisms and recent antibiotic exposure among participants. |
|  | iv. Comparison to prior studies is superficial | We appreciate the reviewer’s constructive suggestion. The discussion has now been expanded to provide more detailed comparison with previous studies from Tanzania and East Africa regarding prevalence of CA-UTIs, dominant uropathogens, ESBL production, and multidrug resistance patterns. Additional comparisons with studies from Mwanza, Dar es Salaam, Uganda, Kenya, and Ghana have been incorporated to better contextualize the findings within the regional antimicrobial resistance landscape. |
| 7. | Limitations need to be discussed more extensively | We thank the reviewer for this valuable recommendation. In response, we have substantially expanded the study limitations section to provide a more balanced interpretation of the findings. |
| 8. | Conclusions a. Public health recommendations are generic. | We thank the reviewer for this important observation. In response, we have revised the public health implications and recommendations section to make the recommendations more specific, actionable, and directly linked to findings from this study. |
| 9. | References a. Referencing format needs to be consistent  b. Keep the references with the past 5 years | We thank the reviewer for these important observations. The reference list and in-text citations have been carefully reviewed and revised to ensure consistency with the journal referencing style. In addition, older references were updated where more recent and relevant literature was available. However, a few older references were retained because they represent standard methodological references, foundational epidemiological literature, or internationally accepted guidelines relevant to the study methodology and interpretation. |
|  | Reviewer #2 |  |
|  | General feedback: The study looks more like it was conducted retrospectively with whatever data was available rather than prospectively with definite aims and objectives and does have some technical knowledge gaps. Moreover, the public health implications mentioned are general and not solely based on this study. | We thank the reviewer for these important observations. We would like to clarify that the study was prospectively conducted using a predefined study protocol, clearly stated objectives, eligibility criteria, standardized data collection tools, and laboratory procedures. Participants were consecutively enrolled during the study period, and microbiological analyses were performed systematically according to standard operating procedures and CLSI guidelines. To improve clarity, we have revised the methodology section to explicitly state the prospective nature of the study and further clarify the study objectives. |
|  | The study participants were included with probable UTI but later very few show symptoms, so how were these patients identified in the OPD? Please elaborate. | Participants were enrolled after initial clinical assessment by outpatient clinicians based on symptoms suggestive of urinary tract infection according to Tanzanian Standard Treatment Guidelines. Eligibility required the presence of at least two symptoms suggestive of UTI, including dysuria, urinary frequency, urgency, suprapubic pain, or lower abdominal pain. However, many participants presented with non-classical symptoms such as lower abdominal pain, fever, or lower back pain, which are commonly encountered in routine primary healthcare practice and often prompt empirical treatment for suspected UTI. We have clarified this process in the methodology and discussion sections to improve understanding of patient recruitment and the observed low frequency of classical urinary symptoms. |
|  | Please add the methodology of ESBL detection. Do follow CLSI guidelines (as cited) for antibiotic testing for various microorganisms. | We thank the reviewer for this important methodological observation. In response, we have expanded the methodology section to include the procedure used for ESBL screening and confirmation according to CLSI guidelines. We also carefully reviewed the antimicrobial susceptibility testing and interpretation to ensure compliance with CLSI recommendations for organism-specific antibiotic testing. Inappropriate organism-antibiotic combinations and interpretations not recommended by CLSI were revised or removed accordingly. |
|  | Abstract: Please keep all scientific name italicized e.g., Escherichia coli | We thank the reviewer for this observation, the entire document have been revised an scientific naming edited accordingly |
|  | Some of the suggestions previously made were still not addressed! Line 195, Table 2:  Streptococcus pyogenes is not commonly reported in urine. Please check and confirm the identity or do not include. | We thank the reviewer for this important observation. We acknowledge that *Streptococcus pyogenes* is an uncommon urinary isolate. However, all microbiological procedures were performed according to standard operating procedures and quality control measures, including culture, colony quantification, Gram staining, biochemical identification, and confirmation of unidentified isolates using MALDI-TOF where necessary. The isolates identified as *S. pyogenes* demonstrated significant bacteriuria based on the predefined study criteria and were therefore retained in the analysis. Nevertheless, because of the uncommon nature of this organism in urinary tract infections, we have revised the discussion to emphasize cautious interpretation of its clinical significance. |
|  | Lines 207-209: You mention 20% ESBL but no mention of third-generation cephalosporin resistance. | We thank the reviewer for this important observation. We agree that reporting ESBL prevalence without corresponding third-generation cephalosporin resistance data may limit interpretation of the findings. In response, we have revised the results and discussion sections to explicitly report resistance to ceftriaxone among Enterobacterales isolates alongside ESBL prevalence. |
|  | Line 212: How were ESBLs detected? Please elaborate in methodology. | We thank the reviewer for this important methodological observation. In response, we have expanded the methodology section to include the procedure used for ESBL screening and confirmation according to CLSI guidelines. |
|  | Lines 213-214: You mention resistance of Pseudomonas to Ampicillin, amoxy-clav and ceftriaxone but these are NOT the recommended antibiotics for Pseudomonas as the organism has intrinsic resistance towards these antibiotics. | We thank the reviewer for this important methodological observation. We agree that these antibiotics are not recommended for susceptibility interpretation in *Pseudomonas* spp. due to intrinsic resistance. The antimicrobial susceptibility results and corresponding interpretations have been carefully revised according to CLSI recommendations, and only organism-antibiotic combinations corresponding to recommended and clinically relevant testing are currently presented in the revised manuscript. |
|  | Line 216 and 219: Erythromycin is not to be reported for urinary isolates of Gram negative organisms or S.aureus. You can use Erythromycin to screen for Macrolide-Lincosamide resistance using the D-test and mention the details in methodology but exclude it from resistance and MDR analyses. | We thank the reviewer for this important methodological observation. We agree that erythromycin should not be included in routine susceptibility reporting or multidrug resistance analysis for urinary isolates in this context. In response, the antimicrobial susceptibility results and MDR analyses have been revised according to CLSI recommendations, and erythromycin has been excluded from resistance reporting and MDR calculations. The methodology section has also been updated to clarify that erythromycin was used only for screening inducible macrolide-lincosamide resistance using the D-test where applicable. |
|  | Line 223, Table 3: Nitrofurantoin is not applicable for Pseudomonas. | We thank the reviewer for this important methodological observation. We agree that nitrofurantoin is not recommended for susceptibility interpretation in *Pseudomonas* spp. In response, the antimicrobial susceptibility results and corresponding interpretations have been revised according to CLSI recommendations, and nitrofurantoin susceptibility reporting for *Pseudomonas* spp. and other non-fermentative Gram-negative bacteria has been removed from the revised manuscript. |
|  | Lines 322-323: As you have collected the co-morbidities data, you should analyze the correlation. | We thank the reviewer for this valuable suggestion. We agree that analysis of the relationship between comorbidities and microbiologically confirmed urinary tract infection would provide additional clinical insights. However, the current study was primarily designed as a descriptive cross-sectional study focusing on the prevalence, bacterial etiologies, and antimicrobial resistance patterns of community-acquired urinary tract infections among adult outpatients. Detailed analytical assessment of associations between comorbidities and microbiological outcomes was therefore beyond the primary scope of this manuscript. |
|  | Lines 370-374: For the public health implications to be true, the information regarding current status has to be provided. Whenever providing conclusions or recommendations, please limit it to your study findings. If your study had provided local treatment guideline details and then compared with the empiric treatment in these canters, then only can you conclude that this is the public health implication. | We thank the reviewer for this important observation. We agree that conclusions and recommendations should be directly supported by findings generated from the current study. In response, the public health implications and recommendations section has been substantially revised to focus specifically on the observed antimicrobial resistance patterns, multidrug resistance burden, and diagnostic challenges identified in this study. Generalized statements and unsupported conclusions have been minimized or removed. We also incorporated reference to the Tanzanian Standard Treatment Guidelines in the introduction and discussion sections to contextualize the observed resistance patterns in relation to commonly recommended empirical therapies. |
|  | Lines 384-388: You can’t generalize the comments, they have to be observed in your study and then only discussed. | We thank the reviewer for this important observation. We agree that conclusions and recommendations should be restricted to findings directly generated from the current study. In response, the generalized statements previously included in the public health implications section have been revised and minimized. The revised section now focuses specifically on findings observed in this study, including the high prevalence of multidrug-resistant uropathogens, resistance to commonly prescribed antibiotics, and the need for improved diagnostic and antimicrobial stewardship practices at primary healthcare facilities. |

**Comments sent incorporated in the manuscript draft within track changes**

| **S/No.** | **Reviewer Comment** | **Response to the comment** |
| --- | --- | --- |
|  | **Reviewer I Comments included in the document** | |
| R1-1 | Clarify burden and implications of antimicrobial resistance in community-acquired UTI. | We thank the reviewer for this important comment. We have strengthened the abstract and discussion to better emphasize the public health implications of antimicrobial resistance (AMR), particularly the impact of multidrug resistance on empirical treatment failure and outpatient management in primary healthcare settings. |
| R1-2 | Clarify clinical relevance of atypical presentations. | We thank the reviewer for this important observation. We have clarified the clinical relevance of atypical presentations in the results and discussion sections. The findings likely reflect the true picture of patients managed with UTI diagnosis at primary healthcare facilities, where many patients present with non-specific symptoms and are treated empirically in the absence of microbiological confirmation. These results emphasize the need to strengthen the diagnosis and management of UTIs at lower health facilities, including improved diagnostic approaches and antimicrobial stewardship, as part of ongoing efforts to reduce inappropriate antibiotic use and combat antimicrobial resistance. |
| R1-3 | Explain implications of resistance findings for empirical therapy. | We thank the reviewer for this important comment. We have revised the discussion and conclusion sections to clarify that the high resistance observed to commonly prescribed antibiotics suggests reduced effectiveness of current empirical treatment practices for community-acquired UTIs at primary healthcare facilities. These findings highlight the importance of reviewing empirical treatment approaches based on local antimicrobial susceptibility patterns to improve patient management and reduce inappropriate antibiotic use. |
| R1-4 | Strengthen public health recommendations. | We thank the reviewer for this constructive recommendation. We expanded the recommendations section to include actionable public health interventions focusing on antimicrobial stewardship, surveillance, diagnostic strengthening, and community education. |
| R1-5 | Improve rationale for studying primary healthcare facilities instead of tertiary hospitals. | We have revised the introduction to clarify that most uncomplicated UTIs are initially managed at primary healthcare level, where empirical treatment is common and microbiological diagnostic capacity is limited. |
| R1-6 | Clarify knowledge gap. | We thank the reviewer for this important point. A clear knowledge gap statement has now been added emphasizing the limited recent surveillance data on community-acquired UTI and AMR patterns among adult outpatients at primary healthcare facilities in Tanzania. |
| R1-7 | Link empirical treatment failure to AMR and public health outcomes. | We have strengthened the introduction and discussion to connect inappropriate empirical treatment, antimicrobial resistance, treatment failure, and broader public health consequences including morbidity and increased healthcare burden. |
| R1-8 | Discussion should interpret antibiotics that are failing and those still useful. | We appreciate this important comment. We revised the discussion and recommendations to clearly distinguish antibiotics with high resistance rates from those that may still be considered for empirical therapy. |
| R1-9 | Recommendations on improved diagnostic capacity are vague. | We have expanded this section substantially by recommending point-of-care urine testing, urine culture access, clinical decision support tools, healthcare worker training, and referral systems for complicated infections. |
| R1-10 | Introduction is too broad and not sufficiently focused on Tanzanian AMR context. | We thank the reviewer for this valuable guidance. The introduction has been substantially revised and streamlined to focus on the Tanzanian context, AMR burden, outpatient management, and study rationale. |
| R1-11 | Introduction can be shortened. | We agree with the reviewer. The introduction has been condensed and repetitive/general statements removed while preserving essential context. |
| R1-12 | Methodology is appropriate. | We thank the reviewer for the positive assessment of our methodology and laboratory procedures. |
|  | **Reviewer II Comments included in the document** | |
| R2-1 | Rename title and italicize organisms throughout manuscript. | We thank the reviewer for this observation. The title has been revised, and all bacterial species names have been italicized throughout the manuscript. |
| R2-2 | Provide references for statements regarding MDR prevalence and risk factors. | Appropriate references have been added to support statements regarding MDR prevalence and factors associated with CA-UTI and AMR. |
| R2-3 | Provide citation for urine HCG testing methodology. | We thank the reviewer. A citation describing urine pregnancy testing methodology has been added. |
| R2-4 | Provide citation for MALDI-TOF methodology. | We appreciate this comment. The MALDI-TOF identification methodology and relevant reference have now been included. |
| R2-5 | Clarify discrepancy between 221 confirmed cases and 194 bacterial isolates. | We thank the reviewer for identifying this inconsistency. We clarified that the 221 microbiologically confirmed cases included both bacterial isolates and Candida spp. Specifically, 194 cases yielded bacterial isolates while 27 had significant Candida growth. |
| R2-6 | How is *Streptococcus pyogenes* reported in UTI? | We thank the reviewer for this important observation. Although *Streptococcus pyogenes* is an uncommon uropathogen, rare cases have been documented, particularly in association with perineal colonization or ascending genital tract infection. We have clarified this in the discussion and advised cautious interpretation of its clinical significance. |
| R2-7 | Clarify unusual finding of *S. aureus* in uncomplicated UTI. | We appreciate the reviewer’s concern. We clarified that *S. aureus* is an uncommon cause of uncomplicated UTI and may indicate complicated infection or hematogenous spread. We further explained that detailed evaluation for systemic involvement was beyond the scope of this study, but identification procedures followed standard microbiological methods, minimizing risk of misidentification. |
| R2-8 | Improve grammatical consistency and scientific wording. | We thank the reviewer for this important observation. The manuscript has been carefully edited for grammar, sentence structure, consistency of terminology, and scientific clarity throughout. |
| R2-9 | Clarify multidrug resistance percentages and resistance patterns. | We carefully reviewed the MDR results and harmonized the values reported across the abstract, results, tables, and discussion to ensure consistency. |
| R2-10 | Clarify clinical implications of resistance findings. | We revised the discussion and conclusion to better explain how the observed resistance patterns may affect empirical prescribing practices in outpatient settings. |
