# Supplementary material for: Multidrug resistance in bacteria causing community-acquired urinary tract infections among adult outpatients attending primary healthcare facilities in Mwanza, Tanzania
Source: PLOS Glob Public Health. 2026 Oct 8;6(10):e0005638. doi: 10.1371/journal.pgph.0005638 (PMC13649018; doi:10.1371/journal.pgph.0005638)
Supplement: Supplementary file 8 [file pgph.0005638.s011.docx]

| **No.** | **Reviewer 1** | |
| --- | --- | --- |
|  | **Reviewer comment** | **Author response** |
| 1. | The manuscript’s strength is the provision of recent local data from primary care rather than tertiary facilities, using a large sample size. The findings directly inform empirical treatment choices at primary-care level. | We thank the reviewer for recognizing the relevance and strength of the study. We agree that data generated directly from primary healthcare facilities are important because these facilities are often the first point of care and empirical antibiotic prescribing commonly occurs before microbiological confirmation. |
| 2. | The title is clear and descriptive and includes the location and study population. | We thank the reviewer for this positive assessment. |
| 3. | The abstract is concise and appropriately presents key findings, including the prevalence, predominant organisms and atypical clinical presentations. | We thank the reviewer for this positive observation. |
| 4. | Resistance rates are listed without clinical interpretation. | We thank the reviewer for this important comment. We have revised the abstract to clarify that the high resistance observed to commonly prescribed antibiotics indicates reduced likely effectiveness of these agents for empirical treatment of community-acquired UTI in this setting. |
| 5. | Public-health implications are vague; “improved diagnostic capacity” is overly general and not sufficiently supported by the findings. | We agree that the previous wording was broad. The abstract has been revised to specify the relevant actions supported by the study: strengthening local antimicrobial susceptibility surveillance, reviewing empirical treatment recommendations, improving access to basic UTI diagnostics and promoting antimicrobial stewardship at primary healthcare facilities. |
| 6. | The introduction correctly identifies the knowledge gap, particularly the limited evidence from general adult outpatients attending primary healthcare facilities. | We thank the reviewer for recognizing the relevance of the identified knowledge gap. |
| 7. | The introduction appropriately acknowledges that AMR patterns vary geographically and over time. | We thank the reviewer for this positive observation. |
| 8. | The introduction is still long and overly detailed. | We agree. The introduction has been shortened by removing general information that was not essential to the study rationale and by consolidating overlapping statements on UTI burden, risk factors and antimicrobial resistance. |
| 9. | The manuscript does not state what Tanzanian empirical-treatment guidelines currently recommend or explain why those recommendations may be challenged. | We thank the reviewer for this clinically important comment. The introduction now states the antibiotics recommended in the Tanzanian Standard Treatment Guidelines for empirical UTI management and explains that high local resistance may compromise their effectiveness, particularly where culture and susceptibility testing are not routinely available. |
|  | **Methods – positive observations** | |
| 10. | The sample-size calculation using the Kish–Leslie formula is clear. | We thank the reviewer for this positive assessment. |
| 11. | Appropriate exclusion criteria have been listed. | We thank the reviewer for this observation. |
| 12. | Quality control, ethical approval and informed consent are appropriately documented. | We thank the reviewer for recognizing these methodological strengths. |
|  | **Methods – CLSI and susceptibility testing** | |
| 13. | Erythromycin testing against urinary *S. aureus* is not recommended for routine urinary susceptibility reporting by CLSI. | We thank the reviewer for identifying this issue. Erythromycin had been used as part of screening for inducible clindamycin resistance using the D-test rather than as a routine therapeutic option for UTI. We have clarified this purpose and removed erythromycin from routine urinary susceptibility reporting and from MDR classification. |
| 14. | Amoxicillin-clavulanic acid, trimethoprim-sulfamethoxazole, ceftriaxone and ampicillin were reported for non-fermentative Gram-negative bacteria despite not being appropriate organism–antibiotic combinations. | We agree. The susceptibility database and tables have been rechecked against CLSI recommendations and intrinsic-resistance principles. Results for non-recommended organism–antibiotic combinations have been removed and were not used in MDR classification. |
| 15. | Nitrofurantoin was tested against *Proteus* and *Pseudomonas*, which are intrinsically resistant. | We agree with the reviewer. Intrinsic resistance should not be interpreted as acquired resistance or included in routine susceptibility summaries. Nitrofurantoin results for intrinsically resistant organisms have therefore been excluded. |
| 16. | Prior antibiotic-use recall was limited to 14 days, whereas AMR studies often use 30–90 days. | We thank the reviewer for this methodological observation. The 14-day period was selected to improve the reliability of self-reported exposure and to capture recent antibiotic use that might suppress bacterial growth and affect culture positivity. However, we acknowledge that this period may not capture earlier exposures relevant to selection of resistant organisms. |
| 17. | No statistical-analysis plan was described. | We agree. The Data Analysis subsection has been expanded to state how continuous and categorical variables were summarized, which comparative tests were applied and the statistical significance threshold. |
|  | **Results** | |
| 18. | Table 3 and Figure 2 present the same data. | We agree that presenting identical information in both formats was unnecessary. Therefore, figure two have been deleted. |
| 19. | The finding that 24.7% of isolates were *S. aureus* is unusual for community-acquired UTI and may have been overinterpreted without adequately considering contamination. | We thank the reviewer for this important comment. We agree that *S. aureus* is an uncommon cause of uncomplicated community-acquired UTI and that contamination or colonization cannot be completely excluded from a single midstream urine specimen. We have therefore revised the manuscript to provide a more cautious interpretation. However, all isolates met the study definition of significant bacteriuria and were identified using standard microbiological methods. Given the high prevalence of multidrug-resistant (83.3%) and methicillin-resistant (64.6%) *S. aureus*, we believe reporting these isolates remains important for community antimicrobial resistance surveillance. |
| 20. | Candida was detected in 27 patients but was not explored; candiduria commonly represents colonization or contamination rather than true Candida UTI. | We thank the reviewer for this valuable comment. We agree that candiduria may represent colonization, contamination, or true infection depending on the clinical context. However, characterization of *Candida* isolates, including species identification, antifungal susceptibility testing, and assessment of their clinical significance, was beyond the scope of the present study, which focused on bacterial pathogens causing community-acquired UTIs and their antimicrobial resistance patterns. |
| 21. | No analysis was performed to examine the association between symptoms and microbiological confirmation, although this could increase the study’s impact. | We thank the reviewer for this valuable suggestion. We agree that evaluating the relationship between clinical presentation and microbiological confirmation would provide additional clinical insights. However, this analysis was beyond the scope of the present study, which was designed primarily to determine the prevalence of community-acquired UTIs, characterize bacterial pathogens, and describe their antimicrobial resistance patterns. We have acknowledged this as a limitation and recommend that future studies investigate the diagnostic value of clinical symptoms in predicting microbiologically confirmed UTIs. |
|  | **Discussion** | |
| 22. | The discussion is generally appropriate. | We thank the reviewer for this positive assessment.  The overall structure has been retained while the specific concerns below have been addressed. |
| 23. | The interpretation of *S. aureus* is excessive. | We agree. The discussion has been revised to provide a more cautious interpretation, acknowledging that *S. aureus* is an uncommon cause of uncomplicated community-acquired UTI and that its isolation from midstream urine may represent contamination, colonization, or true infection. We also note that the study design did not permit differentiation between these possibilities. |
| 24. | There is no discussion of *Candida* species. | We thank the reviewer for this valuable comment. We agree that species-level characterization of *Candida* would provide additional clinical insights. However, this was beyond the objectives of the present study, which focused on bacterial pathogens causing community-acquired urinary tract infections and their antimicrobial resistance patterns. |
| 25. | Drivers of AMR are discussed generically, including self-medication and over-the-counter access, without linking them to the study data. | We thank the reviewer for this important comment. We agree that the previous discussion included potential drivers of antimicrobial resistance that were not directly assessed in this study. The discussion has been revised to distinguish findings supported by our data from contextual evidence reported in the literature. Statements regarding self-medication, over-the-counter antibiotic access, and other behavioral factors are now presented as possible explanations rather than conclusions drawn from this study. |
|  | Reviewer 2 |  |
| 1. | Some of the comments I had given earlier were still not addressed. You have given the reference of CLSI document for antibiotic susceptibility testing but the results still include NOT recommended and intrinsically resistant antibiotics in your analysis. Some organisms that are contaminants or colonizers have been reported as pathogens. You have made conclusions on very small numbers of organisms isolated (e.g. Citrobacter). Please revise and conduct a grammar check too. | We thank the reviewer for carefully re-evaluating our revised manuscript and for highlighting these remaining concerns. We have comprehensively reviewed the manuscript and addressed each point raised. Specifically, the antimicrobial susceptibility results have been re-evaluated in accordance with the latest CLSI recommendations, and all inappropriate organism–antibiotic combinations and intrinsically resistant organism–antibiotic combinations have been removed from the analyses. We have revised the interpretation of atypical isolates to acknowledge the possibility of contamination or colonization, avoided drawing conclusions based on organisms with very small numbers of isolates, and thoroughly edited the manuscript to improve grammar, clarity, consistency, and scientific presentation. Detailed responses to each concern are provided below. |
