# Supplementary material for: Multidrug resistance in bacteria causing community-acquired urinary tract infections among adult outpatients attending primary healthcare facilities in Mwanza, Tanzania
Source: PLOS Glob Public Health. 2026 Oct 8;6(10):e0005638. doi: 10.1371/journal.pgph.0005638 (PMC13649018; doi:10.1371/journal.pgph.0005638)
Supplement: Peer review history

# Peer review: Decision Letter 0

**DOI:** 10.1371/journal.pgph.0005638.r001

**Authors:** Emmanuel Olamijuwon

30 Jan 2026

PGPH-D-25-03665

Multidrug resistance bacteria causing community acquired urinary tract infections among adult outpatients attending lower-level health facilities in Mwanza, Tanzania

PLOS Global Public Health

Dear Dr. mushi,

Thank you for submitting your manuscript to PLOS Global Public Health. After careful consideration, we feel that it has merit but does not fully meet PLOS Global Public Health’s publication criteria as it currently stands. Therefore, we invite you to submit a revised version of the manuscript that addresses the points raised during the review process.

Please submit your revised manuscript by Mar 16 2026 11:59PM. If you will need more time than this to complete your revisions, please reply to this message or contact the journal office at globalpubhealth@plos.org. When you're ready to submit your revision, log on to https://www.editorialmanager.com/pgph/ and select the 'Submissions Needing Revision' folder to locate your manuscript file.

Please include the following items when submitting your revised manuscript:

- A letter that responds to each point raised by the editor and reviewer(s). You should upload this letter as a separate file labeled 'Response to Reviewers'.
- A marked-up copy of your manuscript that highlights changes made to the original version. You should upload this as a separate file labeled 'Revised Manuscript with Track Changes'.
- An unmarked version of your revised paper without tracked changes. You should upload this as a separate file labeled 'Manuscript'.

Guidelines for resubmitting your figure files are available below the reviewer comments at the end of this letter.

We look forward to receiving your revised manuscript.

Kind regards,

Emmanuel Olamijuwon, Ph.D

Academic Editor

PLOS Global Public Health

Journal Requirements:

1. Please amend your online Financial Disclosure statement. If you did not receive any funding for this study, please simply state: "The authors received no specific funding for this work."

2. Please update your online Competing Interests statement. If you have no competing interests to declare, please state: "The authors have declared that no competing interests exist."

3. We note that your Data Availability Statement is currently as follows: "All data are included in the manuscript"

Please confirm at this time whether or not your submission contains all raw data required to replicate the results of your study. Authors must share the “minimal data set” for their submission. PLOS defines the minimal data set to consist of the data required to replicate all study findings reported in the article, as well as related metadata and methods (https://journals.plos.org/globalpublichealth/s/data-availability#loc-minimal-data-set-definition).

For example, authors should submit the following data:

- The values behind the means, standard deviations and other measures reported;

- The values used to build graphs;

- The points extracted from images for analysis.

Authors do not need to submit their entire data set if only a portion of the data was used in the reported study.

If your submission does not contain these data, please either upload them as Supporting Information files or deposit them to a stable, public repository and provide us with the relevant URLs, DOIs, or accession numbers. For a list of recommended repositories, please see https://journals.plos.org/globalpublichealth/s/recommended-repositories.

If there are ethical or legal restrictions on sharing a de-identified data set, please explain them in detail (e.g., data contain potentially sensitive information, data are owned by a third-party organization, etc.) and who has imposed them (e.g., an ethics committee). Please also provide contact information for a data access committee, ethics committee, or other institutional body to which data requests may be sent. If data are owned by a third party, please indicate how others may request data access.

4. Please provide separate main figure files in .tif or .eps format only and remove any figures embedded in your manuscript file. Please also ensure that all files are under our size limit of 10MB. Please leave the figure captions in the manuscript.

For more information about how to convert your figure files please see our guidelines: https://journals.plos.org/globalpublichealth/s/figures

5. Please ensure that you refer to your main Tables in your text as, if accepted, production will need these references to link the reader to the tables.

6. Please upload copies of "Supplementary File 1" and "Supplementary file 2", which you refer to in your text, and ensure that the Supporting Information files have legends listed in the manuscript after the References list. Or if the supplementary materials are no longer to be included as part of the submission, please remove all reference to it within the text.

7. If the reviewer comments include a recommendation to cite specific previously published works, please review and evaluate these publications to determine whether they are relevant and should be cited. There is no requirement to cite these works unless the editor has indicated otherwise.

Additional Editor Comments (if provided):

[Note: HTML markup is below. Please do not edit.]

Reviewers' comments:

Reviewer's Responses to Questions

**Comments to the Author**

1. Does this manuscript meet PLOS Global Public Health’s publication criteria?

Reviewer #1: Yes

Reviewer #2: Partly

Reviewer #3: Yes

Reviewer #4: Yes

**********

2. Has the statistical analysis been performed appropriately and rigorously?

Reviewer #1: No

Reviewer #2: No

Reviewer #3: Yes

Reviewer #4: Yes

**********

3. Have the authors made all data underlying the findings in their manuscript fully available (please refer to the Data Availability Statement at the start of the manuscript PDF file)?

The PLOS Data policy

Reviewer #1: Yes

Reviewer #2: Yes

Reviewer #3: Yes

Reviewer #4: No

**********

4. Is the manuscript presented in an intelligible fashion and written in standard English?

Reviewer #1: Yes

Reviewer #2: No

Reviewer #3: Yes

Reviewer #4: Yes

**********

Reviewer #1: General comments: Although it was a well conducted and timely cross-sectional study that address

a significant public health challenges of the rise of AMR in CAUTI in a resource-limited setting. The

study does not add any new knowledge or findings that are not known among the scientific realm.

1. Title

a. Lower-level health facilities – could be more precise maybe primary healthcare facilities

which is what the chosen facilities are but additionally PHC is a public health focus for

achieving UHC which is hindered by the AMR rise

2. Abstract

a. It is informative and methodologically sound

b. Although overly dense it lacks the public health implications and clinical correlates to the

findings

i. Hospital based is slightly misleading as these as health centres which are

primary care

ii. No mention of low rate of classic UTI symptoms which is a major clinical finding

which affects the diagnosis

c. Key results are buried within the list

i. 82.8% MDR in E.coli is not in the abstract – should be featured

ii. >50% resistant to ciprofloxacin, amoxicillin clavulanic acid – is hard to digest

� Make it more palatable to the audience but also at the bottom line

clinicians want to know which first-line drugs are failing

d. Vague recommendations

i. Improved diagnostic capacity – can be further explored as there were atypical

symptoms for the CAUTI in this study – maybe the authors can suggest further

3. Introduction

a. The information is informative but it dilutes the narrative for the study objectives

i. The inclusion to mention risk factors is generic and not specifically tied to the

Tanzanian context or study focus on AMR

ii. Knowledge gap is missing

iii. Rational for studying outpatients in primary care as opposed to hospitals

should be highlighted

iv. A public health framing that links empirical treatment failure to AMR

mortality is also very important

b. The introduction can shorten to be to the point

4. Methodology

a. Good and appropriate

b. Good lab methodologies

c. Some improvements:

i. The massive oversampling needs to have a rationale

ii. Clinical definition

� Cite a guideline where at least two of the following UTI symptoms

� The broad case definition like dysuria may have low specificity – which

should be later noted as a potential limitation

d. Data analysis plan

i. Descriptive data analysis – descriptive data does not have analysis but rather as

presented as medians and proportions

5. Results

a. The results are rich but poorly presented – narrative and clinical translation is imperative

for the presentation of the results

b. Multiple tables which can be reduced

i. Table 1 and 2 are cohort description

� These can be merged where a consolidated table comparing 221

confirmed UTI patients with 784 non-confirmed patients on key

variables

ii. Table 4 & 5 can be presented in a more clinically friendly manner

� Suggested table – utility of common antibiotics for empirical treatment

of CAUTI in observed cohort: pathogen | recommended (susceptibility

>80%) | use with caution (susceptibility 50-79%) | not recommended

(susceptibility <50%)

iii. Tables 6 & 7

� Odds ratio is not the appropriate analysis in this study as the outcome

prevalence (MDR infection) is not rare - >10%

� Use Modified Poisson regression reporting risk ratio

6. Discussion

a. The discussion is very formulaic: restate results � compare to other studies �

limitations and conclusions but misses the opportunity to:

i. Have a public health narrative about causality and intervention

ii. Clinical implication from atypical symptom data

b. Selective critiques:

i. Line 268 to 274 – very broad and very generic

ii. Line 275 to 288

� Most reported prior antibiotic use – but table 2 reported on 12.8%

� Passive public health statement – very generic

� Failure to link to key drivers

iii. Line 289 to 301

� It does not have a clinical translation as what should I prescribe

tomorrow?

� Vague recommendation of updating treatment guidelines to tailor local

resistance?

iv. Line 310-320

� Antimicrobial stewardship and diagnostic stewardship is a very

important element of discussion particularly in the rise of AMR – this

should be explored and not mentioned as a solution

c. Study limitation should be evaluated

Reviewer #2: General comment: Rename title as “Multi-drug resistant bacteria…” OR “Multidrug resistance in bacteria..” and change at all places in the manuscript. Make the organisms’ names italicized in the document.

Line 81: Please provide references for the statements 79-81 and 82-86 with citations in place.

Line 110: Please mention reference of method by which HCG was tested in urine (please cite).

Line 120: Please provide reference of methodology of MALDI-TOF.

Line 171: Please clarify how are there only 194 bacteria when 221 were bacteriologically confirmed?

Table 3: How is S. pyogenes reported in UTI?

Lines 215 & 248: Please remove the antibiotics amoxicillin-clavulanic acid, trimethoprim-sulfamethoxazole, ceftriaxone, and ampicillin from the non-fermenter organism’s list and further results and discussion section too as these are not recommended for these organisms by CLSI! Please provide reference of the CLSI document you referred to and cite accordingly. Even Nitrofurantoin is not recommended for Proteae family organisms and Pseudomonas. Ceftazidime instead of ceftriaxone for non-fermenters.

Line 223: Erythromycin is not tested for urine S. aureus isolates. Please remove at all places.

Lines 225&226: Other gram-positive (e.g. Enterococcus) have different antibiotics requirement so can’t say cumulative resistance to clindamycin if it is not used. Change values in Table 7 accordingly.

Table 6: Please change the MDR values after removing the NOT-required antibiotics!

Line 255: Please change numbers after removing Erythromycin.

Lines 299-301: The numbers of C. freundii were not very high so this statement should have some statistical evidence attached to it.

Please provide limitations of the study and try and analyze your findings statistically (Significant vs not-significant) as the numbers are quite low.

As you have clinical information of CAUTI patients please perform further statistical analyses to find association of symptoms with the type of organism or MDR to make your study findings have more significance.

Reviewer #3: • An interesting study that potentially adds useful information to the etiology and antimicrobial resistance of urinary tract infection

o While information contributed is routine and offers no specific novelty. It adds to the pool of information for the specific location and timing in which it was performed given that etiology and antimicrobial susceptibility profiles can change over time.

o The authors ought to do more in demonstrating the gap that the study aims at addressing

More detailed review in the attached file

Reviewer #4: The study presents relevant data in a simplistic and reproducible manner. I recommend the work should be published. The authors however need to thoroughly proof-read the document to address many typos in the text, this can be done in the proofing stage. They also need to upload the collected data on a repository or explain why the data is not publicly shared.

**********

what does this mean?). If published, this will include your full peer review and any attached files.

**Do you want your identity to be public for this peer review?**  If you choose “no”, your identity will remain anonymous but your review may still be made public.

For information about this choice, including consent withdrawal, please see our Privacy Policy

Reviewer #1: No

Reviewer #2: **Yes:**  Dr. Jyoti Acharya

Reviewer #3: **Yes:**  Henry Kajumbula

Reviewer #4: **Yes:**  Ronald Chitatanga

**********

[NOTE: If reviewer comments were submitted as an attachment file, they will be attached to this email and accessible via the submission site. Please log into your account, locate the manuscript record, and check for the action link "View Attachments". If this link does not appear, there are no attachment files.]

Attachment Submitted filename: Manuscript Review_UTI_TZ.docx

Attachment Submitted filename: TZ_UTI_Manuscript_Review_HKM.pdf

# Author comment: Author response to Decision Letter 1

**DOI:** 10.1371/journal.pgph.0005638.r002

9 Apr 2026

Attachment Submitted filename: rebuttal_table.docx

# Peer review: Decision Letter 1

**DOI:** 10.1371/journal.pgph.0005638.r003

**Authors:** Emmanuel Olamijuwon

4 May 2026

PGPH-D-25-03665R1

Multidrug resistance bacteria causing community acquired urinary tract infections among adult outpatients attending lower-level health facilities in Mwanza, Tanzania

PLOS Global Public Health

Dear Dr. mushi,

Thank you for submitting your manuscript to PLOS Global Public Health. After careful consideration, we feel that it has merit but does not fully meet PLOS Global Public Health’s publication criteria as it currently stands. Therefore, we invite you to submit a revised version of the manuscript that addresses the points raised during the review process.

Please submit your revised manuscript by Jul 03 2026 11:59PM. If you will need more time than this to complete your revisions, please reply to this message or contact the journal office at globalpubhealth@plos.org. When you're ready to submit your revision, log on to https://www.editorialmanager.com/pgph/ and select the 'Submissions Needing Revision' folder to locate your manuscript file.

Please include the following items when submitting your revised manuscript:

- A letter that responds to each point raised by the editor and reviewer(s). You should upload this letter as a separate file labeled 'Response to Reviewers'.
- A marked-up copy of your manuscript that highlights changes made to the original version. You should upload this as a separate file labeled 'Revised Manuscript with Track Changes'.
- An unmarked version of your revised paper without tracked changes. You should upload this as a separate file labeled 'Manuscript'.

Guidelines for resubmitting your figure files are available below the reviewer comments at the end of this letter.

As the corresponding author, your ORCID iD is verified in the submission system and will appear in the published article. PLOS supports the use of ORCID, and we encourage all coauthors to register for an ORCID iD and use it as well. Please encourage your coauthors to verify their ORCID iD within the submission system before final acceptance, as unverified ORCID iDs will not appear in the published article. *Only*  the individual author can complete the verification step; PLOS staff *cannot*  verify ORCID iDs on behalf of authors.

We look forward to receiving your revised manuscript.

Kind regards,

Emmanuel Olamijuwon, Ph.D

Academic Editor

PLOS Global Public Health

Journal Requirements:

If the reviewer comments include a recommendation to cite specific previously published works, please review and evaluate these publications to determine whether they are relevant and should be cited. There is no requirement to cite these works unless the editor has indicated otherwise.

Additional Editor Comments (if provided):

[Note: HTML markup is below. Please do not edit.]

Reviewers' comments:

Reviewer's Responses to Questions

**Comments to the Author**

Reviewer #1: (No Response)

Reviewer #2: All comments have been addressed

**********

publication criteria?

Reviewer #1: Yes

Reviewer #2: Partly

**********

3. Has the statistical analysis been performed appropriately and rigorously?

Reviewer #1: Yes

Reviewer #2: Yes

**********

4. Have the authors made all data underlying the findings in their manuscript fully available (please refer to the Data Availability Statement at the start of the manuscript PDF file)?

The PLOS Data policy

Reviewer #1: Yes

Reviewer #2: Yes

**********

5. Is the manuscript presented in an intelligible fashion and written in standard English?

Reviewer #1: Yes

Reviewer #2: Yes

**********

Reviewer #1: Title: Multidrug resistance bacteria causing community acquired urinary tract infections among adult outpatients attending lower-level health facilities in Mwanza, Tanzania

General comment: The manuscript’s strength is local, recent data from primary care and not tertiary with a large sample size. This directly informs empirical treatment choices at primary care level which is valuable.

1. Title

a. It is clear, descriptive including location and population

2. Abstract

a. The abstract is concise, representing numbers such as 22% prevalence, 32.9% E.coli, 24.7% S. aureus. It also mentions atypical presentations – which is clinically relevant

b. However:

i. Resistance rates are listed but without clinical interpretations

ii. Public health implications are vague – improved diagnostic capacity is very vague and mentioned there as an over generalization of the findings

3. Introduction

a. Correctly identifies the knowledge gap: most prior studies from tertiary hospital or specific population. Acknowledges that AMR patterns vary by geography and time

b. However:

i. It is still very long and over detailed

ii. There is no statement of what empirical guidelines currently recommend in Tanzania and why those may be failing – a clinician needs to know the current standard of care and why is it challenged

4. Methods

a. Clear sample size calculation with Kish-Lesile formula

b. Appropriate exclusion criteria have been listed

c. Quality control described and ethical approval and consent properly documented

d. However;

i. CSLI violation:

• Erythromycin against urine S.aureus – not a CLSI recommendation

• Amoxiclav, cotrimoxazole, ceftriaxone, ampicillin – tested against non-fermenters – not recommendation

• Nitrofurantoin tested against Proteus and Pseudomonas – these are intrinsically resistant

ii. The prior antibiotic use recall period is too short – only 14 days? AMR studies typically use 30-90 days?

iii. No statistical analysis plan described

5. Results

a. Explanation for:

i. Table 3 and figure 2 relay identical data

ii. S-aureus finding over interpreted – 24.7% of isolates S-aureus is highly unusual for CA-UTI - addressing contamination vs true infection – without this, many of these may be skin flora contamination

iii. Candida in 27 patients – mentioned but not explored. Candida UTI is rare and most likely colonization or contamination

iv. No analysis of association between symptoms and microbiology results which would yield high impact

6. Discussion

a. The discussion is appropriate. A few recommendations:

i. The over interpretation of S.aureus

ii. No discussion on the candida species

iii. Drivers of AMR are discussed generically – self-medication, OTC access without linking to this study’s data

iv. Comparison to prior studies is superficial

7. Limitations need to be discussed more extensively

8. Conclusions

a. Public health recommendations are generic.

9. References

a. Referencing format needs to be consistent

b. Keep the references with the past 5 years

Reviewer #2: General feedback:

The study looks more like it was conducted retrospectively with whatever data was available rather than prospectively with definite aims and objectives and does have some technical knowledge gaps. Moreover, the public health implications mentioned are general and not solely based on this study.

The study participants were included with probable UTI but later very few show symptoms, so how were these patients identified in the OPD? Please elaborate. Please add the methodology of ESBL detection. Do follow CLSI guidelines (as cited) for antibiotic testing for various microorganisms.

Abstract: Please keep all scientific name italicized e.g., Escherichia coli

Some of the suggestions previously made were still not addressed!

Line 195, Table 2: Streptococcus pyogenes is not commonly reported in urine. Please check and confirm the identity or do not include.

Lines 207-209: You mention 20% ESBL but no mention of third-generation cephalosporin resistance.

Line 212: How were ESBLs detected? Please elaborate in methodology.

Lines 213-214: You mention resistance of Pseudomonas to Ampicillin, amoxy-clav and ceftriaxone but these are NOT the recommended antibiotics for Pseudomonas as the organism has intrinsic resistance towards these antibiotics.

Line 216 and 219: Erythromycin is not to be reported for urinary isolates of Gram negative organisms or S.aureus. You can use Erythromycin to screen for Macrolide-Lincosamide resistance using the D-test and mention the details in methodology but exclude it from resistance and MDR analyses.

Line 223, Table 3: Nitrofurantoin is not applicable for Pseudomonas.

Lines 322-323: As you have collected the co-morbidities data, you should analyze the correlation.

Lines 370-374: For the public health implications to be true, the information regarding current status has to be provided. Whenever providing conclusions or recommendations, please limit it to your study findings. If your study had provided local treatment guideline details and then compared with the empiric treatment in these canters, then only can you conclude that this is the public health implication.

Lines 384-388: You can’t generalize the comments, they have to be observed in your study and then only discussed.

**********

what does this mean?). If published, this will include your full peer review and any attached files.

**Do you want your identity to be public for this peer review?**  If you choose “no”, your identity will remain anonymous but your review may still be made public.

For information about this choice, including consent withdrawal, please see our Privacy Policy

Reviewer #1: No

Reviewer #2: No

**********

[NOTE: If reviewer comments were submitted as an attachment file, they will be attached to this email and accessible via the submission site. Please log into your account, locate the manuscript record, and check for the action link "View Attachments". If this link does not appear, there are no attachment files.]

Attachment Submitted filename: PGPH-D-25-03665_R1_reviewerfeedback2026.pdf

Attachment Submitted filename: Review MDR Tanzania.docx

# Author comment: Author response to Decision Letter 2

**DOI:** 10.1371/journal.pgph.0005638.r004

20 May 2026

Attachment Submitted filename: response to reviewer round two.docx

# Peer review: Decision Letter 2

**DOI:** 10.1371/journal.pgph.0005638.r005

**Authors:** Ilse Bloom

3 Jun 2026

PGPH-D-25-03665R2

Multidrug resistance bacteria causing community acquired urinary tract infections among adult outpatients attending lower-level health facilities in Mwanza, Tanzania

PLOS Global Public Health

Dear Dr. mushi,

Thank you for submitting your manuscript to PLOS Global Public Health. After careful consideration, we feel that it has merit but does not fully meet PLOS Global Public Health’s publication criteria as it currently stands. Therefore, we invite you to submit a revised version of the manuscript that addresses the points raised during the review process.

The manuscript has been further evaluated by two reviewers, and their comments are available in the attached file/below.

Could you please carefully revise the manuscript to address all comments raised, including those raised by reviewers in previous rounds of review that have not yet been addressed?

Please submit your revised manuscript by Aug 02 2026 11:59PM. If you will need more time than this to complete your revisions, please reply to this message or contact the journal office at globalpubhealth@plos.org. When you're ready to submit your revision, log on to https://www.editorialmanager.com/pgph/ and select the 'Submissions Needing Revision' folder to locate your manuscript file.

Please include the following items when submitting your revised manuscript:

- A letter that responds to each point raised by the editor and reviewer(s). You should upload this letter as a separate file labeled 'Response to Reviewers'.
- A marked-up copy of your manuscript that highlights changes made to the original version. You should upload this as a separate file labeled 'Revised Manuscript with Track Changes'.
- An unmarked version of your revised paper without tracked changes. You should upload this as a separate file labeled 'Manuscript'.

Guidelines for resubmitting your figure files are available below the reviewer comments at the end of this letter.

As the corresponding author, your ORCID iD is verified in the submission system and will appear in the published article. PLOS supports the use of ORCID, and we encourage all coauthors to register for an ORCID iD and use it as well. Please encourage your coauthors to verify their ORCID iD within the submission system before final acceptance, as unverified ORCID iDs will not appear in the published article. *Only*  the individual author can complete the verification step; PLOS staff *cannot*  verify ORCID iDs on behalf of authors.

We look forward to receiving your revised manuscript.

Kind regards,

Ilse Bloom

Staff Editor

PLOS Global Public Health

**Journal Requirements:**

If the reviewer comments include a recommendation to cite specific previously published works, please review and evaluate these publications to determine whether they are relevant and should be cited. There is no requirement to cite these works unless the editor has indicated otherwise.

**Additional Editor Comments (if provided):**

[Note: HTML markup is below. Please do not edit.]

Reviewers' comments:

Reviewer's Responses to Questions

**Comments to the Author**

Reviewer #1: All comments have been addressed

Reviewer #2: (No Response)

**********

publication criteria?

Reviewer #1: Yes

Reviewer #2: Partly

**********

3. Has the statistical analysis been performed appropriately and rigorously?

Reviewer #1: Yes

Reviewer #2: No

**********

4. Have the authors made all data underlying the findings in their manuscript fully available (please refer to the Data Availability Statement at the start of the manuscript PDF file)?

The PLOS Data policy

Reviewer #1: Yes

Reviewer #2: Yes

**********

5. Is the manuscript presented in an intelligible fashion and written in standard English?

Reviewer #1: Yes

Reviewer #2: No

**********

**Reviewer #1:**  Title: Multidrug resistance bacteria causing community acquired urinary tract infections among adult outpatients attending lower-level health facilities in Mwanza, Tanzania

General comment: The manuscript’s strength is local, recent data from primary care and not tertiary with a large sample size. This directly informs empirical treatment choices at primary care level which is valuable.

1. Title

a. It is clear, descriptive including location and population

2. Abstract

a. The abstract is concise, representing numbers such as 22% prevalence, 32.9% E.coli, 24.7% S. aureus. It also mentions atypical presentations – which is clinically relevant

b. However:

i. Resistance rates are listed but without clinical interpretations

ii. Public health implications are vague – improved diagnostic capacity is very vague and mentioned there as an over generalization of the findings

3. Introduction

a. Correctly identifies the knowledge gap: most prior studies from tertiary hospital or specific population. Acknowledges that AMR patterns vary by geography and time

b. However:

i. It is still very long and over detailed

ii. There is no statement of what empirical guidelines currently recommend in Tanzania and why those may be failing – a clinician needs to know the current standard of care and why is it challenged

4. Methods

a. Clear sample size calculation with Kish-Lesile formula

b. Appropriate exclusion criteria have been listed

c. Quality control described and ethical approval and consent properly documented

d. However;

i. CSLI violation:

• Erythromycin against urine S.aureus – not a CLSI recommendation

• Amoxiclav, cotrimoxazole, ceftriaxone, ampicillin – tested against non-fermenters – not recommendation

• Nitrofurantoin tested against Proteus and Pseudomonas – these are intrinsically resistant

ii. The prior antibiotic use recall period is too short – only 14 days? AMR studies typically use 30-90 days?

iii. No statistical analysis plan described

5. Results

a. Explanation for:

i. Table 3 and figure 2 relay identical data

ii. S-aureus finding over interpreted – 24.7% of isolates S-aureus is highly unusual for CA-UTI - addressing contamination vs true infection – without this, many of these may be skin flora contamination

iii. Candida in 27 patients – mentioned but not explored. Candida UTI is rare and most likely colonization or contamination

iv. No analysis of association between symptoms and microbiology results which would yield high impact

6. Discussion

a. The discussion is appropriate. A few recommendations:

i. The over interpretation of S.aureus

ii. No discussion on the candida species

iii. Drivers of AMR are discussed generically – self-medication, OTC access without linking to this study’s data

iv. Comparison to prior studies is superficial

7. Limitations need to be discussed more extensively

8. Conclusions

a. Public health recommendations are generic.

9. References

a. Referencing format needs to be consistent

b. Keep the references with the past 5 years

**Reviewer #2:**  Some of the comments I had given earlier were still not addressed. You have given the reference of CLSI document for antibiotic susceptibility testing but the results still include NOT recommended and intrinsically resistant antibiotics in your analysis. Some organisms that are contaminants or colonizers have been reported as pathogens. You have made conclusions on very small numbers of organisms isolated (e.g. Citrobacter). Please revise and conduct a grammar check too.

**********

what does this mean?). If published, this will include your full peer review and any attached files.

**Do you want your identity to be public for this peer review?**  If you choose “no”, your identity will remain anonymous but your review may still be made public.

For information about this choice, including consent withdrawal, please see our Privacy Policy

Reviewer #1: No

Reviewer #2: No

**********

[NOTE: If reviewer comments were submitted as an attachment file, they will be attached to this email and accessible via the submission site. Please log into your account, locate the manuscript record, and check for the action link "View Attachments". If this link does not appear, there are no attachment files.]

**Figure Resubmissions:**

Attachment Submitted filename: revise manuscript round two clear_feedback.docx

# Author comment: Author response to Decision Letter 3

**DOI:** 10.1371/journal.pgph.0005638.r006

28 Jul 2026

Attachment Submitted filename: response to reviewers comment round three.docx

# Peer review: Decision Letter 3

**DOI:** 10.1371/journal.pgph.0005638.r007

**Authors:** Emmanuel Olamijuwon

8 Sep 2026

Multidrug resistance bacteria causing community acquired urinary tract infections among adult outpatients attending lower-level health facilities in Mwanza, Tanzania

PGPH-D-25-03665R3

Dear Dr mushi,

We are pleased to inform you that your manuscript 'Multidrug resistance bacteria causing community acquired urinary tract infections among adult outpatients attending lower-level health facilities in Mwanza, Tanzania' has been provisionally accepted for publication in PLOS Global Public Health.

Before your manuscript can be formally accepted you will need to complete some formatting changes, which you will receive in a follow up email. A member of our team will be in touch with a set of requests.

Please note that your manuscript will not be scheduled for publication until you have made the required changes, so a swift response is appreciated.

IMPORTANT: The editorial review process is now complete. PLOS will only permit corrections to spelling, formatting or significant scientific errors from this point onwards. Requests for major changes, or any which affect the scientific understanding of your work, will cause delays to the publication date of your manuscript.

If your institution or institutions have a press office, please notify them about your upcoming paper to help maximize its impact. If they'll be preparing press materials, please inform our press team as soon as possible -- no later than 48 hours after receiving the formal acceptance. Your manuscript will remain under strict press embargo until 2 pm Eastern Time on the date of publication. For more information, please contact globalpubhealth@plos.org.

Thank you again for supporting Open Access publishing; we are looking forward to publishing your work in PLOS Global Public Health.

Best regards,

Emmanuel Olamijuwon, Ph.D

Academic Editor

PLOS Global Public Health

***********************************************************

Reviewer Comments (if any, and for reference):

Reviewer's Responses to Questions

**Comments to the Author**

Reviewer #1: All comments have been addressed

**********

publication criteria?

Reviewer #1: Yes

**********

3. Has the statistical analysis been performed appropriately and rigorously?

Reviewer #1: Yes

**********

4. Have the authors made all data underlying the findings in their manuscript fully available (please refer to the Data Availability Statement at the start of the manuscript PDF file)?

The PLOS Data policy

Reviewer #1: Yes

**********

5. Is the manuscript presented in an intelligible fashion and written in standard English?

Reviewer #1: Yes

**********

Reviewer #1: all comments were addressed

**********

what does this mean?). If published, this will include your full peer review and any attached files.

**Do you want your identity to be public for this peer review?**  If you choose “no”, your identity will remain anonymous but your review may still be made public.

For information about this choice, including consent withdrawal, please see our Privacy Policy

Reviewer #1: No

**********
